# Bicarbonate transporters in corals point towards a key step in the evolution of cnidarian calcification

**DOI:** 10.1038/srep09983

**Published:** 2015-06-04

**Authors:** Didier Zoccola, Philippe Ganot, Anthony Bertucci, Natacha Caminiti-Segonds, Nathalie Techer, Christian R Voolstra, Manuel Aranda, Eric Tambutté, Denis Allemand, Joseph R Casey, Sylvie Tambutté

**Affiliations:** 1 Centre Scientifique de Monaco, 8 quai Antoine Ier, Monaco, 98000, Monaco; 2 ARC Centre of Excellence for Coral Reef Studies, James Cook University, Townsville, QLD 4811, Australia; 3 Red Sea Research Center, King Abdullah University of Science and Technology (KAUST), Thuwal, Saudi Arabia; 4 Department of Biochemistry, University of Alberta Edmonton, Alberta T6G 2H7, Canada

## Abstract

The bicarbonate ion (HCO_3_^−^) is involved in two major physiological processes in corals, biomineralization and photosynthesis, yet no molecular data on bicarbonate transporters are available. Here, we characterized plasma membrane-type HCO_3_^−^ transporters in the scleractinian coral *Stylophora pistillata*. Eight solute carrier (SLC) genes were found in the genome: five homologs of mammalian-type SLC4 family members, and three of mammalian-type SLC26 family members. Using relative expression analysis and immunostaining, we analyzed the cellular distribution of these transporters and conducted phylogenetic analyses to determine the extent of conservation among cnidarian model organisms. Our data suggest that the SLC4γ isoform is specific to scleractinian corals and responsible for supplying HCO_3_^−^ to the site of calcification. Taken together, SLC4γ appears to be one of the key genes for skeleton building in corals, which bears profound implications for our understanding of coral biomineralization and the evolution of scleractinian corals within cnidarians.

Symbiotic cnidarians from the order Scleractinia form the foundation of coral reefs that constitute one of the most important biogenic structures worldwide. Coral reefs provide habitat and trophic support for myriad of marine species, the richness of which rivals the biological diversity of tropical rainforests^1^. Despite their environmental significance, key elements of coral physiology, such as the symbiotic interactions between the animal host and its intracellular photosynthetic dinoflagellates of the genus *Symbiodinium,* or the biomineralization process underlying the formation of the coral skeleton, are poorly understood. This is largely due to knowledge gaps in fundamental aspects of cnidarian cell biology^2^,^3^.

Photosynthesis by the symbionts and the biomineralization process both involve the use of dissolved inorganic carbon (DIC). In seawater, DIC exists in the form of chemically inter-convertible molecules that exist in a pH dependent equilibrium: the non-ionic form, CO_2_, with a concentration on the order of 10 μM at normal seawater pH of 8.1, and two ionic forms, HCO_3_^−^ and CO_3_^2−^, with concentrations of up to 200 times higher (i.e. ~2.4 mM). Photosynthesis and calcification, however, occur in compartments that are not in direct contact with seawater and thus need to be actively supplied with DIC. For instance, the intracellular symbionts are located in the endodermal tissue layer, separated from seawater by the ectoderm tissue layer. Further, symbionts are separated from the host cytoplasm by the perisymbiotic membrane^4^. In order to secure continuous provision of DIC despite these constraints, the coral has developed CO_2_-concentrating mechanisms to absorb and transfer DIC from the seawater to its symbionts for photosynthesis^5^. DIC uptake by the host involves an H^+^-ATPase that acidifies the ectodermal boundary layer where bicarbonate (HCO_3_^−^) is converted to CO_2_ by a membrane-bound isoform of carbonic anhydrase (CA). The uncharged CO_2_ molecule then diffuses into the epidermal cells. Once in the animal cytoplasm, another CA isoform is involved in the equilibration between CO_2_ and HCO_3_^−^ according to the intracellular pH^6^, which prevents CO_2_ back-diffusion (for review see^7^). The mechanism of DIC transport through the remaining membranes to the symbionts is currently debated, but it is accepted that bicarbonate has to exit the ectodermal cells to subsequently enter the endodermal cells by a bicarbonate anion transporter (BAT) (for reviews, see^5^,^8^). Physiological experiments with radioactive tracers together with pharmacological experiments using classic bicarbonate transport inhibitors, such as 4,4’-Diisothiocyano-2,2’-stilbenedisulfonic acid (DIDS)^9^, support the contribution of BATs in the supply of DIC required for photosynthesis by the symbiont (see Fig. 1,^5^,^8^). However, no molecular data on BATs in cnidarians are currently available.

The calcification process itself occurs at a site that is separated from seawater by four tissue layers: the coral oral ectoderm and endoderm, as well as the coral aboral endoderm and ectoderm. The major portion of the DIC used for CaCO_3_ formation comes from metabolic CO_2_^5^,^10^, which is produced by the calcifying cells forming the aboral ectoderm (also called the calicoblastic ectoderm). Part of this metabolic CO_2_ may diffuse across the plasma membrane, whereas another part is hydrated into HCO_3_^−^ due to the alkaline intracellular pH^5^,^6^. The proportion of CO_2_ diffusion *versus* CO_2_ hydration is currently unknown. However, inhibition of anion transport with DIDS also inhibits calcification^5^,^11^ indicating that BATs are likely involved in the transport of HCO_3_^−^ across the calcifying cells to the site of calcification, but further data on the molecular characteristics of the transporter(s) involved is lacking (Fig. 1).

In Mammals, two distinct families of membrane BATs are differentiated: solute carrier 4 (SLC4) (for review,^12^,^13^) and solute carrier 26 (SLC26)^14^ transporters. The SLC4 family represents the majority of HCO_3_^−^ transporters, whereas the SLC26 family consists of members that can transport diverse ions besides HCO_3_^−^. Mammalian BATs consist of 14 genes, nine SLC4 members and five from the SLC26 family^15^. SLC4 family members are separated into three functional groups: i) Na^+^-independent Cl^−^/HCO3^−^ exchangers, mediating electroneutral exchange of Cl^−^ for HCO_3_^−^; ii) Na^+^-HCO_3_^−^ co-transporters mediating the co-transport of Na^+^ and HCO_3_^−^ and iii) Na^+^-driven Cl^−^/HCO_3_^−^ exchangers (NDCBE) mediating the electroneutral exchange of Cl^−^ for Na^+^ and HCO_3_^−^. Within these groups, proteins are separated according to their phylogenetic position, tissue distribution, anion selectivity, regulatory properties, and mechanism of action. For example, the first group contains SLC4A1 (also named Band 3 or AE1), SLC4A2 (or AE2), and SLC4A3 (or AE3); the second group contains SLC4A4 (or NBC1), SLC4A5 (or NBC4), SLC4A7 (or NBC3), SLC4A9 (or AE4), and SLC4A10 (or NCBE); NDCBE is only represented by SLC4A8. SLC4A11 (or BTR1) was originally reported as a sodium/borate co-transporter^16^, but more recently found to facilitate water and Na^+^ fluxes^17^,^18^. The human SLC26 family consists of 11 members, where SLC26A10 is likely a pseudogene^19^. Similar to SLC4 family members, SLC26 family members can be grouped into three groups: i) the SO_4_^2^ transporters, ii) the Cl^−^/HCO_3_^−^ exchangers (also called the SLC26 BATs), and iii) the selective Cl^−^ channels (it should be noted that this last group has minimal HCO3^−^ permeability^19^). The group of Cl^−^/HCO_3_^−^ exchangers consists of either electroneutral (SLC26A3, SLC26A4, and SLC26A6) or electrogenic (SLC26A7, and SLC26A9) transporters^15^.

Besides a physiological understanding of the process of calcification, insights into the extent of conservation of gene families within Cnidaria might hold key insights for our understanding of the evolution of this phylum. For instance, an evolutionary analysis of the innate immunity gene repertoire among Scleractinia (i.e. *Acropora millepora*), Actinaria (i.e. *Nematostella*), and Hydrozoa (i.e. *Hydra*) revealed an extended Toll-like receptor (TLR) gene set in corals in line with the symbiotic lifestyle of corals^20^.

To gain insight into the composition and evolution of coral BATs, we performed genome data mining and monitored expression and localization of eight identified putative solute carrier (SLC) proteins in the coral *Stylophora pistillata,* the species from which many of the aforementioned physiological data were obtained. Our present study aimed at defining a comprehensive analysis of the repertoire of these transporters and to discuss their potential role in symbiosis and biomineralization. Further, we were interested in the distribution of these transporters across key cnidarian taxa to contribute to our understanding of the evolution of scleractinian corals within the phylum Cnidaria.

## Results

### Candidate HCO_3_^−^ Transporters in *Stylophora pistillata*

Coral genomes have a remarkably high level of conservation with vertebrates in comparison to their invertebrate counterparts, such as flies and nematodes^21^. We identified eight candidate HCO_3_^−^ anion transporter (BAT) genes (Fig. 2 and File S1) in the genome of *S. pistillata* (genome sequence provided by C.R.V. and M.A, personal communication, in collaboration with D. Z., S. T., and D.A.,^22^. Of these genes, five (referred to as SpiSLC4α, β, γ, δ, and ε), and three (referred to as SpiSLC26α, β, and γ) were similar to members of the mammalian SLC4A and SLC26A family, respectively. With the exception of SpiSLC4α, these genes were expressed and found in *S. pistillata* EST libraries^23^. In addition, we have identified homologs of these genes in the coral *A. digitifera* and the sea anemone *N. vectensis* (Table S2), two anthozoans for which a genome sequence is available^20^,^24^. Comparison between homologs and paralogs of the three species showed that, surprisingly, no homolog of the scleractinian SLC4γ was found in the *N. vectensis* genome (Table S3). Searches in the transcriptome^25^ and genome (which CEGMA pourcentage completeness is 96.72%) of another anemone, *Aiptasia pallida* (Baumgarten, S., Simakov, O., Esherick, L. Y., Liew, Y. J., Lehnert, E. M., Michell, C. T., Li, Y., Hambleton, E. A., Guse, A., Oates, M. E., Gough, J., Weis, V. M., Aranda, M., Pringle, J. R. & Voolstra, C. R. ; personal communication) , confirmed the absence of this SLC4γ homolog in actinarians. Phylogenetic reconstruction using human and cnidarian homologs (Fig. 3) indicated that the cnidarian SLC4 members fall into the categories previously described^13^,^15^. These categories were electroneutral (human SLC4A7, A8, and A10) and electrogenic (human SLC4A4, A5, and A9) Na^+^-coupled Cl^−^/HCO_3_^−^ co-transporters, Na^+^-independent Cl^−^/HCO_3_^−^ exchangers (human SLC4A1, A2, and A3), and Na^+^/borate co-transporters (human SLC4A11). Furthermore, evolutionary relationships indicate that the SLC26 members fall into the two phylogenetic groups described by Dorwart *et al.*^19^ consisting of Cl^−^/HCO3^−^ exchangers and selective Cl^−^ channels.

### Sequence analysis

Structure prediction showed that the *S. pistillata* SLC4 family members (in the following referred to as SpiSLC4s) exhibit the canonical three domain pattern shared in all SLC4 transporters^13^,^26^: a long N-terminal hydrophilic domain of 424, 380, 511, 496, and 362 amino acids for SpiSLC4α, β, γ, δ, and ε respectively, and a short C-terminal hydrophilic domain (Fig. 2). Both termini are known to be intracellular^27^. The central part consisted of 11-13 predicted transmembrane segments, although the exact number of transmembrane segments remains unknown due to possible re-entrant loops in the C-terminal half of some membrane domains^13^. With regard to post-translational modifications, Asn577 and Asn528 were predicted to be N-glycosylated in SpiSLC4α and SpiSLC4ε respectively. Potential N-glycosylation sites in the other proteins were located in the cytoplasmic part of the proteins and, consequently, did not support glycosylation. Furthermore, we predicted 31, 44, 56, 47, and 33 phosphorylation sites for SpiSLC4 α, β, γ, δ, and ε, respectively. Of note, the prominent BAT inhibitors, the stilbene inhibitors DIDS and H_2_DIDS^28^, covalently label Lys^539^ and/or Lys^851^ in human SLC4A1; the first Lys residue was present only in SpiSLC4δ (amino acid 630), whereas the second was conserved in all the isoforms (amino acid 876, 827, 961, and 976 for SpiSLC4α, β, γ, and δ respectively).

Structural analysis of SLC26 proteins showed that *S. pistillata* SLC26 members (in the following referred to as SpiSLC26s) exhibited shorter N-terminal domains than the SLC4 family (112, 121, and 45 amino acids for SpiSLC26α, β, and γ respectively). The C-terminal domains of all the mammalian SLC26 proteins included a sulfate transporter domain and an anti-sigma factor antagonist (STAS) domain^29^. Similarly, the three *S. pistillata* proteins also shared the sulfate transporter domain (amino acids 234–511 for SpiSLC26α, amino acids 228–505 for SpiSLC26β, and 131–433 for SpiSLC26γ) and the STAS domain (amino acids 567–738 for SpiSLC26α, amino acids 561–708 for SpiSLC26β, and 476–569 for SpiSLC26γ), as shown by CDD blast analysis^30^. Potential glycosylation sites were located at the amino acid 294 for SpiSLC26α, amino acid 389 for SpiSLC26β, and amino acid 291 for SpiSLC26γ. Phosphorylation site predictions identified 40, 42, and 20 phosphorylation sites for SpiSLC26α, β, and γ respectively.

### Gene structure

The genomic structure of the different BAT genes was established (Fig. 4 and Table 1) using the SIM4 algorithm^31^. Genomic sequences are given in File S4. It should be noted that SpiSLC4β and SpiSLC4γ are on the same scaffold and separated by 18017 bp, and that SpiSLC26α and SpiSLC26β genes are also on the same scaffold and separated by 2491 bp.

### Tissue distribution and localization of the different isoforms

**Figure 1:**
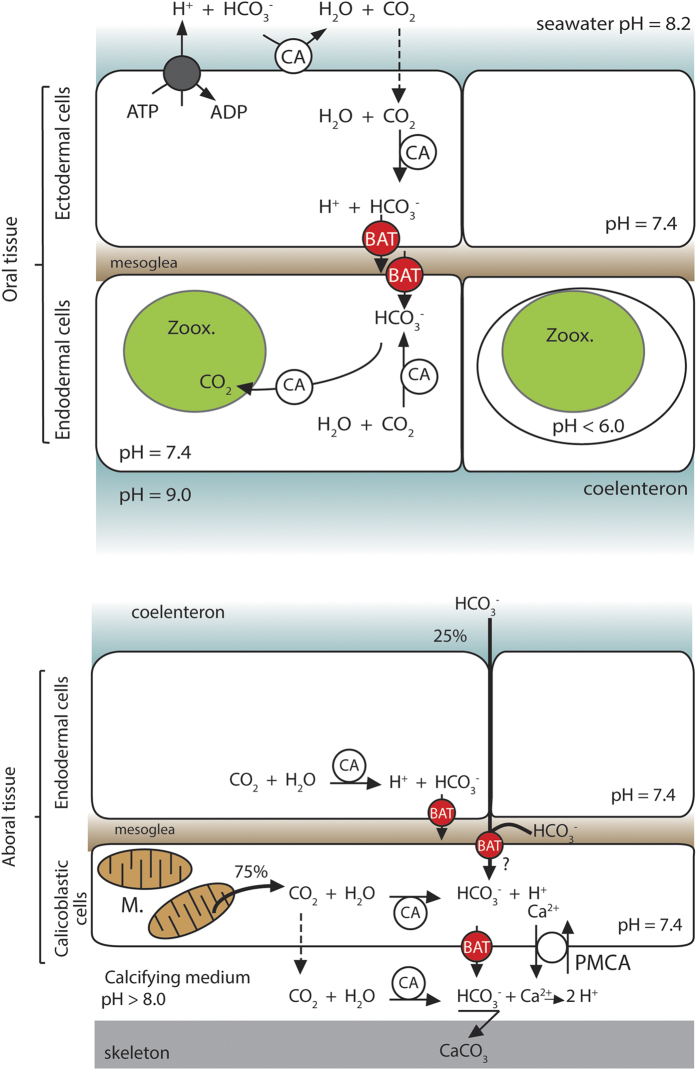
Structural histology and model of DIC transport through the different coral tissue layers (modified from Bertucci *et al*^60^). Dotted arrows represent CO2 diffusion, the projected involvement of BATs is indicated by red circles. Zoox.: zooxanthellae; M.: mitochondrion. CA: Carbonic Anhydrase; PMCA: plasma membrane Calcium ATPase. Unknown mechanisms of DIC transport are indicated with a question mark

Relative expression PCR was performed on colony-wide RNA (total) and oral discs RNA obtained by micro-dissection (32, and Fig. 5A). Experiments were performed on three independent colonies grown in the aquarium facilities of the Centre Scientifique de Monaco. Expression in the two fractions were normalized to acidic ribosomal phosphoprotein P0 expression (36B4), which is not affected by experimental conditions or tissue specificity^33^. SpiSLC26α and γ showed higher expression in oral tissues whereas SpiSLC26β was ubiquitously expressed in the different tissues of *S. pistillata* (Fig. 5B). With regard to the expression of the SLC4 family members, we did not detect expression of SpiSLC4α in coral tissues, as observed for transcriptomic data (see above). SpiSLC4β was expressed almost twice as high in oral tissue, while SpiSLC4δ showed ubiquitous expression. Intriguingly, SLC4γ was either absent or very weakly expressed in the oral fraction, suggesting a specific expression in the aboral tissues. The expression of SpiSLC4ε showed large inter-individual variation, which prevented further interpretation. In the following, we confirmed gene expression results with protein localization using specific antibodies raised against the ubiquitously expressed SpiSLC26β, and the highly specifically expressed SpiSLC4γ. Paraffin-embedded cross-sections of *S. pistillata* encompassing the different tissues (see Fig. 1 and^34^,^35^ for histology details) were used to visualize the proteins using specific antibodies (Fig. 6). Sections of *S. pistillata* tissues labeled with the anti-SpiSLC26β antibody (Fig. 6 B) and magnification thereof (Fig. 6 C) confirmed that its location was ubiquitous, with signals in the ectoderm and endoderm of both oral and aboral tissues. In contrast, immunolocalization of SpiSLC4γ localized this protein to the calicoblastic ectoderm (i.e. the calcifying cell layer), clearly associated with the plasma membrane (Fig. 6 E and F). Negative controls, using preimmune serum showed no labeling (Fig. 6 A and D).

## Discussion

In this study, we provided a molecular characterization of BATs in the coral *Stylophora pistillata* and showed the presence of bicarbonate anion transporters families SCL4 and SCL26 in this coral. Further, we conducted a phylogenetic analysis of these protein families in other cnidarian taxa.

**Figure 2:**
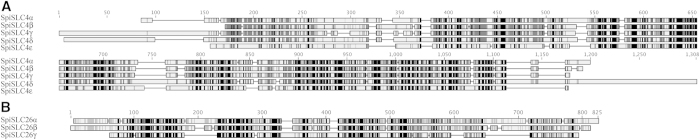
Comparison of *Stylophora pistillata* sequences of (**A**) SLC4 family proteins, and (**B**) SLC26 family proteins using ClustalW alignment using Genious. Legend for colored boxes is 100% similar in black, >80% in dark grey, >60% in medium grey, less than 60% in light grey.

**Figure 3:**
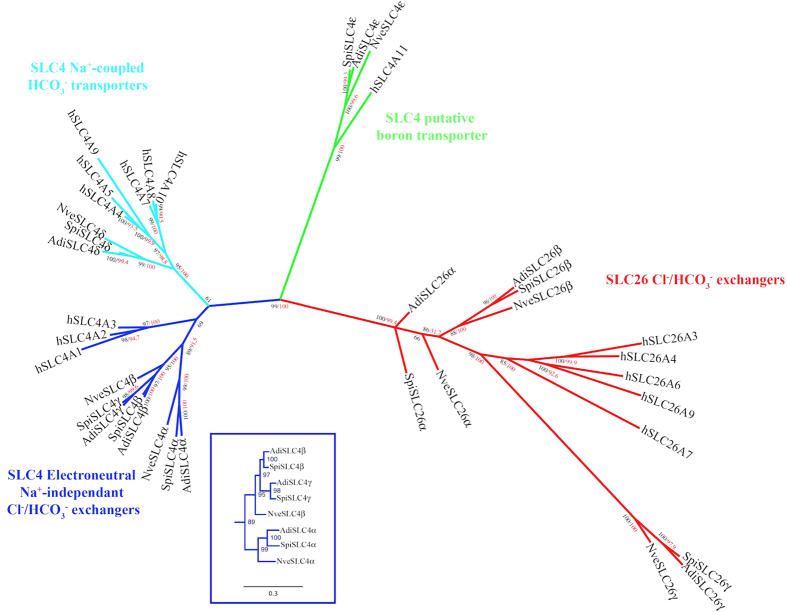
Phylogenetic relationships of human and anthozoan Bicarbonate Anion Transporter protein sequences inferred from Maximum Likelihood (ML) and Bayesian analyses. Bootstrap network of BAT sequences based on ML distances are estimated with a LG+G model (α = 0.749) using PHYML. Bayesian posterior probabilities are indicated in black whereas ML bootstrap values are in red.

**Figure 4:**
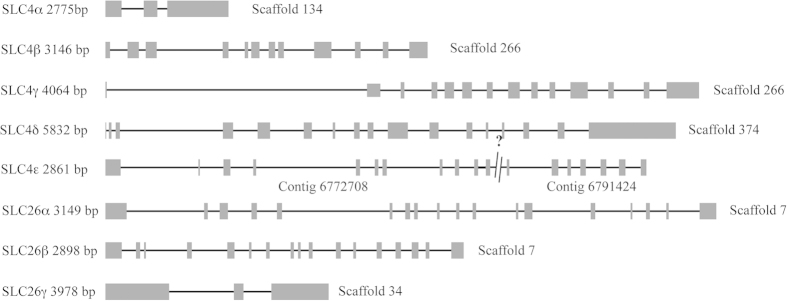
Exon/intron organization of the different BATs in the genome of *Stylophora pistillata*. Exons are represented as boxes whereas introns are depicted as lines.

Analysis of partial sequences of the starlet anemone *Nematostella vectensis,* led to the suggestion that Na^+^-coupled HCO_3_^−^ transporters SLC4 appeared first in Cnidaria^13^. The characterization of the full-length SpiSLC4δ and its *Acropora* homolog shows undoubtedly that these transporters are indeed present in Cnidaria. While the earlier analysis reported four members of SLC4 in the starlet anemone, we could identify five members in the corals *S. pistillata* and *Acropora digitifera* (Fig. 2 and 3). We hypothesize that this is due to a scleractinian specific gene duplication of a Cl^−^/HCO_3_^−^ exchanger as shown in the phylogenic tree (Fig. 3). Interestingly, in *A. digitifera*, AdiSLC4β and AdiSLC4γ were present on the same scaffold separated by 64977 bp in a forward-reverse orientation, whereas in *S. pistillata*, SpiSLC4β and SpiSLC4γ were separated by 18017 bp in a forward-forward orientation. Gene duplication is a main mechanism through which new genetic material is generated during molecular evolution and can lead to evolutionary innovation^36^. Indeed, a SpiSLC4β homolog is present in sea anemones (*N. vectensis* and *A. pallida*) and corals (*A. millepora* and *S. pistillata*), whereas SpiSLC4γ was only present in hard corals. Thus, SpiSLC4β and SpiSLC4γ seem to be paralogs, and should be referred as SpiSLC4β1 and SpiSLC4β2. However, since our findings suggest that SpiSLC4γ has acquired a specific role in skeleton building, we decided to keep distinct names for both genes. The assumed neofunctionalization of SpiSLC4γ is further supported by the highly specific expression and restricted distribution of this gene within coral tissues (see below).

**Figure 5:**
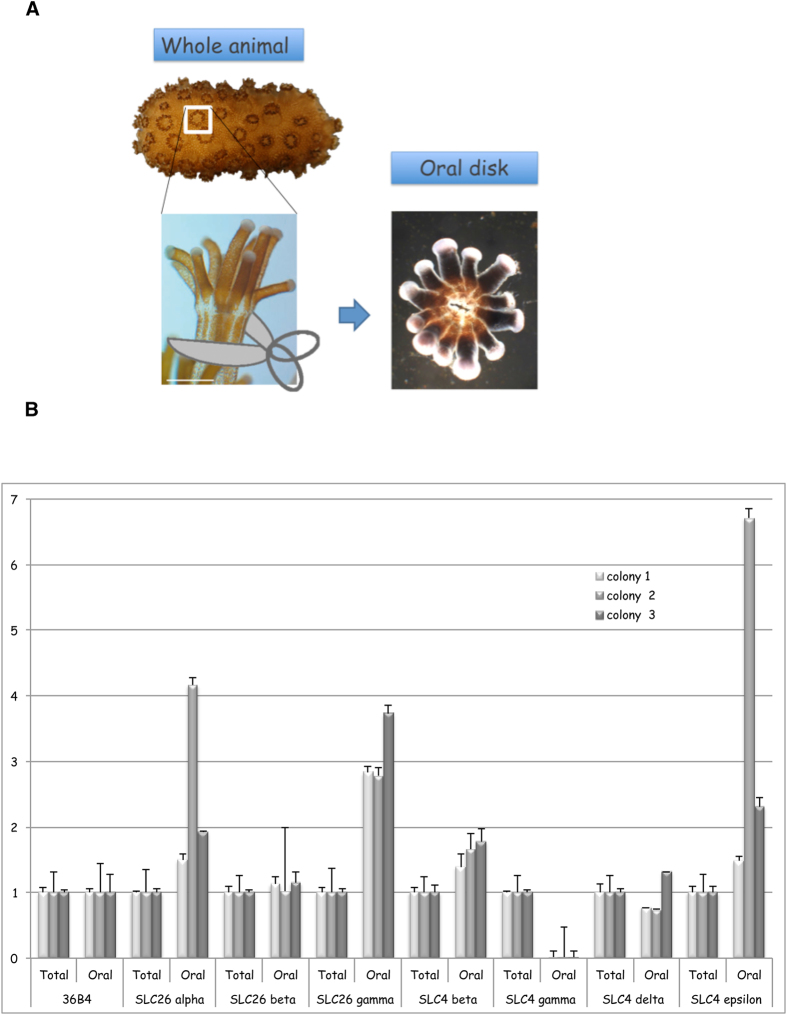
Relative expression of the different BAT proteins in coral tissues based on qPCR. (**A**) cDNA are prepared from total tissues (whole coral fragment), or from oral disc. (**B**) Gene expression normalized to 36B4 expression in total tissues (Total), or in oral disc (Oral). Grey bars represent different colonies. Error bars represent the SD of three technical replicates of each colony.

**Figure 6:**
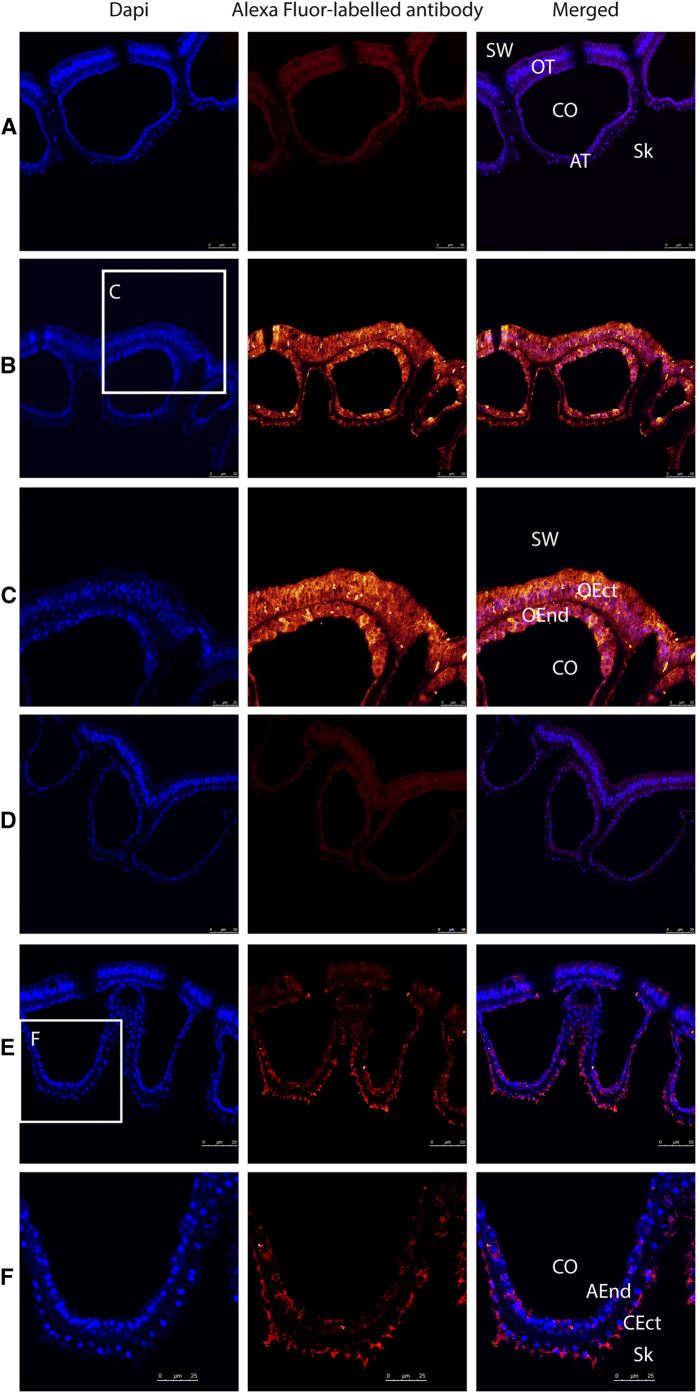
Immunolocalization of SpiSLC26β and SpiSLC4γ. Embedded cross-section of *Stylophora pistillata* tissues labeled by (**A**) preimmune serum for SpiSLC26β, (**B**) and (**C**) anti-SpiSLC26β, (**D**) preimmune serum for SpiSLC4γ, and (**E**) and (**F**) anti- SpiSLC4γ antibody. Rows (**A**), (**B**), (**D**), and (**E**) are views of the four tissues composing the coral. (**C**) are magnifications of oral tissues and (**F**) are magnifications of aboral tissues. Nuclei are labeled in blue in first column (DAPI), streptavidin AlexaFluor 568 fluorescence appears in orange in second column, merged is in the third column. The background red color in cross-section (**A**) and (**D**) with preimmune serum corresponds to autofluorescence of coral tissues. AEnd = Aboral Endoderm; AT = Aboral Tissue; CEct = Calicoblastic Ectoderm; Co = Coelenteron; m = Mesoglea; OEct = Oral Ectoderm; OEnd = Oral Endoderm; OT = Oral Tissue; Sw = Seawater; Sk = Skeleton

Pharmacological experiments with BAT inhibitors are unable to discriminate whether SLC26 or SLC4 BATs are involved in calcification and photosynthesis. Stilbene dilfonates inhibit bicarbonate transport because of their dual hydrophobic/anionic character^9^. Some of these compounds (like DIDS) have covalently-reactive isothiocyanate groups that covalently label specific lysine residues in SLC4 family proteins. Stilbene disulfonates, however, also inhibit anion transporters non-covalently, meaning that that will also target SLC26 proteins lacking the conserved DIDS-reactive lysine residues. Assessment of BAT tissue expression patterns was thus necessary to define the BATs involved in calcification and symbiosis. Relative expression analysis (Fig. 5) of SpiSLC4α shows that this protein is not expressed in adult tissues, suggesting that it could be specific to the embryonic stages. SpiSLC4δ, the putative sodium-coupled transporter, and SLC26β are ubiquitously expressed in all tissues. Therefore they are unlikely to play a specific role in photosynthesis or calcification, but may have a role in homeostatic control of processes, including cell pH, volume, and bicarbonate metabolism. The higher relative expression of SpiSLC26α, SpiSLC26γ, and SpiSLC4β in oral tissues (Fig. 5) suggests that these transporters rather play a role in symbiosis than in calcification. Indeed, as outlined in the introduction, bicarbonate is transported by ectodermal cells, then enters endodermal cells, and finally is delivered to the dinoflagellate symbionts. All these steps of transcellular transport thus involve BATs and we suggest that SpiSLC4β is one of the proteins responsible for this function. Moreover the COOH-terminal tail of SLC4 proteins contain one or more acidic motifs that may serve as a binding site for the cytoplasmic carbonic anhydrase II (CAII)^37^. In SpiSLC4β, this domain is located between residues 861 and 865 (LDNEE). The cytoplasmic binding of CAII and the simultaneous interaction of SLC4 anion exchangers with carbonic anhydrases have been proposed to constitute a bicarbonate transport metabolon^37^,^38^. Carbonic anhydrases play a major role in carbon supply for photosynthesis in corals^5^, and further, a cytoplasmic CA is located in symbiotic endodermal cells^39^. This suggests that the BAT SpiSLC4β protein might be coupled to a cytoplasmic carbonic anhydrase, such as STPCA2, and could serve to accelerate transmembrane bicarbonate transport^40^.

Finally, in our data SpiSLC4γ was completely absent from oral coral tissues, it has not been found in sea anemones, and it localizes to the ectodermal calcifying cells of corals. These data strongly suggest that SpiSLC4γ plays a key role in calcification and might represent one of the evolutionary key innovation genes for calcification in Scleractinia. The calcification process produces protons that need to be removed from the site of calcification in order to favor the precipitation of calcium carbonate^41^,^42^. It has been proposed that removal of protons occurs through a plasma membrane Ca^2+^-ATPase that is located in the membranes of the calcifying cells and which functions as a proton exchanger (Fig. 1)^43^. In addition, the observed increase in pH to values higher than seawater pH^44^ could be performed by supplying bicarbonate at the site of calcification. Such a role might be performed by the here-characterized SpiSLC4γ. Indeed, in mammals BATs are able to neutralize stomach acid entering the intestine by secreting high concentrations of bicarbonate^45^. Sodium dependent- and independent-SLC4, together with SLC26 have been proposed to be involved in this mechanism^46^. Our results suggest that BATs are involved in supplying bicarbonate to the site of calcification and possibly play a role in extracellular pH regulation.

Over recent decades, coral reefs have been impacted by the effects of global environmental change^47^ and are now threatened by ocean acidification^48^. Several experimental studies have shown that a low pH may have detrimental effects on calcification rates and skeletal growth of various coral species^49^,^50^,^51^,^52^. Transcripts corresponding to SLC4 members were up-regulated in response to a CO_2_-driven pH decrease experiment with *Pocillopora damicornis*^53^, suggesting that BATs may play a role in coral resilience when facing environmental acidification conditions. The emergence of the gene SLC4γ via gene duplication appears to be a key adaptation in the evolution of calcification in scleractinian corals. In this context, the role of HCO_3_^−^ in supplying DIC for coral calcification, as well as further elucidation of the function and evolution of BATs and SLC4γ in particular, is fundamental for determining the response of coral reefs to ocean acidification.

## Methods

### Coral culture

Experiments were conducted in the laboratory using the zooxanthellate scleractinian coral *Stylophora pistillata*. Colonies were cultivated as indicated previously^11^.

### Data mining

Sequences homologous to human SLC4 and SLC26 amino acid sequences from NCBI (http://www.ncbi.nlm.nih.gov/protein) were identified amongst *Stylophora pistillata* ESTs obtained with 454 pyrosequencing^23^ or Illumina^22^, using the TblastN algorithm. Furthermore, we used a draft assembly of the *S. pistillata* genome (unpublished data). A CEGMA analysis^54^ reports the conserved proteome to be 94.5% complete, which is similar to the *A. digitifera* genome. *Acropora digitifera* and *Nematostella vectensis* sequences were retrieved from the following web servers: http://marinegenomics.oist.jp/genomes/ and http://www.cnidariangenomes.org/download/nve.gene_ models.vie130208/, respectively.

### Sequence analysis

Putative transmembrane domains were predicted using the Phobius server^55^. Phosphorylation and N-glycosylation prediction analyses were performed with NetPhos and NetNGlyc respectively on the Center for Biological Sequence analysis prediction server (http://www.cbs.dtu.dk/services/).

### Phylogenetic constructions

Phylogenetic trees were constructed with both Maximum Likelihood and Bayesian methods in order to assess result congruencies. ClustalW alignments of all amino acids sequences were performed using MultAlin^56^ with the Blosum62 default parameters. Based upon amino acid alignment, maximum likelihood estimates of the topology and branch length were obtained using PhyML v3.0^57^ with the LG+G model of substitution as recommended by alignment analysis with ProtTest v3.4^58^. Further, phylogenetic relationships were investigated using Bayesian methods as implemented in MrBayes v3.1.2^59^ starting from a random tree, using the LG model of amino acid substitution generating trees for 6,000,000 generations with sampling every 1000 generations, and with four chains in order to obtain the final (consensus) tree and to determine the posterior probabilities at the different nodes.

### Oral disc dissection

Fragments of *Stylophora pistillata* colony were used for RNA extractions. They were set to rest in a glass petri dish filled with sea water until polyps were extended. Tricaine mesylate (MS-222, Sigma) dissolved in sea water to 0.4% was added into the petri dish to a final concentration of 0.04% and incubated under dimmed light for 15 min. Subsequently, viewed under a binocular microscope oral discs (the apparent portion of the polyp, Fig. 5A) were cut from the colony, using micro-dissection scissors with 5 mm blades (Vannas). Batches of 10–15 oral discs were collected and transferred into Trizol^®^. Dissections were stopped after a maximum of 45 min of MS-222 incubation to elude any potential secondary effect of the drug.

### Real-time PCR

Total RNA extraction and cDNA synthesis were performed as described previously^39^. Briefly, cDNAs were synthesized using the Superscript^®^III kit (Invitrogen). qPCR runs were performed, as in^33^, on an ABi 7300 using “EXPRESS SYBR^®^ GreenER^®^ qPCR Supermix with Premixed ROX” for PCR amplification. Primers used are listed in Supplementary Table S5. Relative expressions were calculated using Biogazelle qbase +2.6™.

### Custom made antibodies

Antibodies against SpiSLC26β and SpiSLC4γ were produced in rabbit using synthetic peptides (Eurogentec). Anti-SpiSLC26β was generated against the peptide MESSPGERSIHRQSPE - (amino acids 12-27) and against the peptide DKGNSNRGNPGSKPK (amino acids 624-638). Anti-SpiSLC4γ was raised against the peptide SESNYEGDHSHDDSR - (amino acids 25-39) and against the peptide VTEGFKPTQHDKRGW (amino acids 744-758). For each antibody, ten rabbits were initially screened for non-cross reactivity with S. *pistillata* proteins and two were selected for the Speedy program. Each selected antibody was affinity purified with peptide columns by Eurogentec before use.

### Immunolocalization

Apexes of colonies were prepared for immunolocalization as described previously^34^. Briefly, apexes of *S. pistillata* were fixed in 3% paraformaldehyde in S22 buffer (450 mM NaCl, 10 mM KCl, 58 mM MgCl_2_, 10 mM CaCl_2_, 100 mM Hepes, pH 7.8) at 4 °C overnight and then decalcified, using 0.5 M ethylenediaminetetraacetic acid (EDTA) in Ca-free S22 at 4 °C. They were then dehydrated in an ethanol series and embedded in Paraplast. Cross-sections (6 μm thick) were cut and mounted on silane-coated glass slides. After cutting, deparaffinized sections of tissues were incubated for 1 h in saturating medium (1% BSA, 0.2% teleostean gelatin, 0.05% Tween 20 in Phosphate-buffered saline (PBS) pH 7.4) at 20 °C. Samples were then incubated with the anti-SpiSLC26β or SpiSLC4γ (10 μg/ml) as primary antibody. After rinsing in saturating medium, samples were incubated with biotinylated anti-rabbit antibodies as secondary antibodies. After rinsing with PBS, pH 7.4, samples were finally stained for 15 min with streptavidin AlexaFluor 568 (Molecular probes, 1:50 dilution) and 4’,6-diamidino-2-phenylindole, DAPI (Sigma, 2 μg mL^−1^). Samples were embedded in Pro-Long antifade medium (Molecular Probes) and observed with confocal laser-scanning microscope (Leica, SP5). Immunostaining experiment controls were performed with pre-immune serum for SpiSLC26β or SpiSLC4γ, and then treated with biotinylated anti-rabbit antibodies and streptavidin AlexaFluor 568 as described above.

## Additional Information

**How to cite this article**: Zoccola, D. *et al*. Bicarbonate transporters in corals point towards a key step in the evolution of cnidarian calcification. *Sci. Rep.* **5**, 9983; doi: 10.1038/srep09983 (2015).

## Supplementary Material

Supplementary Information

## Figures and Tables

**Table 1:** Genomic structure of the different BAT genes

**Gene**	**Contig Name**	**mRNA length (pb)**	**Number of exons**	**gene length (pb)**
SpiSLC4 α	Scaffold_134	2775	3	3763
SpiSLC4 β	Scaffold_266	3146	12	9895
SpiSLC4 γ	Scaffold_266	4064	14	18089
SpiSLC4 δ	Scaffold_374	5832	17	17387
SpiSLC4 ε	Contig 6772708/6791424	2861	21	12343
SpiSLC26 α	Scaffold_7	3149	18	18618
SpiSLC26 β	Scaffold_7	2898	17	10918
SpiSLC26 γ	Scaffold_34	3978	3	6818
