# Supplementary material for: Bicarbonate transporters in corals point towards a key step in the evolution of cnidarian calcification
Source: Sci Rep. 2015 Jun 4;5:9983. doi: 10.1038/srep09983 (PMC4650655; doi:10.1038/srep09983)
Supplement: Supplementary Information [file srep09983-s1.pdf]

Bicarbonate transporters in corals point towards a key step in the evolution of cnidarian calcification

Didier Zoccola, Philippe Ganot, Anthony Bertucci, Natacha Caminiti-Segonds, Nathalie Techer, Christian R Voolstra, Manuel Aranda, Eric Tambutté, Denis Allemand, Joseph R Casey, and Sylvie Tambutté

File S1. cDNA sequences of SpiBATs

>Spi_SLC4α (Contig6766636)
ATGACCTCTATCGAAAATATTCCGATTGATCAATCCACTTGTTCTCCGTATGAAGGATTTGTGGAATTGGAGGAACTAGT
CAGTTGTGATGATGAACTGATTTGGAAAGAAAGTGCGCGGTGGATAAAGCTCGAAGAAGACGTCGATGAATGTGCCGATC
GATGGGGGAAACCTCATGTGCCGTGTTTGACATATCGGAGCCTTAGAGAGGTTGAAACGAATCTTCAAAATGGAGTGTGT
TTGTTGGATCTCAATAGAGATACTCTGCCTTCCATTTGGGATGCAATTATTGATGAAATCAAAACGCTGAAAAAGATGGA
AGATAGTGAATGCCTCGTAGTGAAAACTCTTCTAATGACAAGACATGAACATCATCATCAGCATAAAAGTGGAAAACGAG
TTCAAAACTTCGCCAGGCGCCGGTCACGGATCATGCCTAATGGACAGCGCTCCAGGACGAATGTTTCGGAAGATGTCCAC
AATTTAAGAGAAAGACCGCAGCTTAGTCTCGAACAAAACATGTTACCCTCCATTGACAGCAAACAAGATGTCAGCGAGTT
TCCAGCAAACGTAGCAGTGAGTTTCAGCCCTGATCCCGTTGAAGGGTTCGTCGAACAGTTTGACGATCACAACAAAAAAT
CAGATCTGAGAGGGAGACTTCCGGAAAATTCTCAAGCAACCACTGTTTTGGTTGGGGCTCATAATGGCTTGTCTTGCACC
GTCAGTGCTTTTATAAGACTCGCCAAAGGATGCGAACTGAGTAACTTAGCTGAAGTACAGATTCCTATCAGGTTTATTTT
TATCCTGGTGGGACCTAACGATGACAGGATAGACTATCATGAAGTCGGGCGATCTTTTGCAACTCTTATGGCAGATAAAC
TCTTCCTGGAATCTGCTTATCTGGCCAAGCGTCAGCAGGATCTTCTGGATTCTCTATTCAGCTTTATGGGAGACAGCCTC
ATAATTCCTCCAGGAGACTGGGATCGAAATCTACTAAGTCAAACAATACCCATCTTAGCCAGTCAAACCAAGGAATTAGC
CTTAAGGAGACGAAGCGGAGGAATACAAAGCAAGGACAAACCAAATGAAAGATTTCGCGAGGGTTTTCAAGAAAATGAAG
TGTGTGATGACCCTTTGTCTAGAACGGGAAAATTTTGTGGTGGACTTATACGAGACGTTAAAAGGCGATTTCCTTTCTAC
TTGAGTGATTTCAAAGACGCTTTGGACGTTCATTGTCTACCAACAATCATCTTTGTGTATTTCGCTTGCCTCGCTCCTAC
AATCGCCTTCGGCGGTTTGCTGAGCGAGAAAACCAACGCATGGATGGGAGTTTCAGAGATGATATTTGCAACAGCATTAT
CAGGAGTTCTATTTGGCCTCTTTGCAGGACAGCCATTGATCATAATAGGAGCTACGGGTCCATTACTCATTTTCGAGAAG
AGTATATTTCAATTGAGCGAGCGATTTGAGATTGAGTTTCTCCCTTGGCGAGCTTGGGTTGGAATATGGGTGATGGTGAT
ATGTTTTATCATTGTCGCCGTTGAGGGATGTTTTCTCATCAAATATTTTACACGTTTTACAGAAGAAATATTTGCCTTAG
TGATATCTATCATTTTTATTTACTACGCATTTAATTATGTGAGACATATCTTTGATCTGTATCCTTTGAACGCAGACGTC
GCTACTACTCTGTTGGTTCCAAAAAATTCGACCAATCCTACGAAAGGAAAATCTGTAGGGAATCAGTCAACGGCGAACAC
AGCTCTGCTCACCACAGTTTTGATGCTGGGAACCTTCTTTGTCGCTTACACCTTGAGAAAGTTACATCACAGTCATTTCT
TTGGACCCAAAGCTCGTCGCATAGTCAGTGATTTTGGGGTTTTCATCGCTATTGTTTCCATGGTGACAGTTGGTTGGTTG
ACAGAAGGTGTGTACACTCAAAAGTTATCAGTCCCGGATGGTTTTTCTGTTTCCTCACCCAACAGAAATAGCTGGCTTAT
TAACCCCATGGGAGAACAACAGCACATGAGTGCTGTCGCCATTTCTGGAGCTTTCATTCCCGCAGTTCTTGTCAGCATCT
TGATTTTCATGGAAATTGAATTCACCGGGATTATTCTGGACAAAAAAGAACACAAATTGAAGAAAGGCGTGGGTTACAAC
CTAGATCTCTTTGTTCTGGGACTACTAGTTGGCCTGTGCTCAGTTCTGGGCCTTCCGTGGATGTGTGCCACCCCTGTTCA
TACATTGTCTCACTTTCACGCCCTGACAGTGCTTAGTACTAACCATGCACCGGGGGAACATCCTCGTCTGGTACAAGTCA
GAGAACAACGCCTCACGAACATTGTGATCCACCTTCTTATTGGCTTCACTGTTCTTCTATCGCCGGTGATGCGTCTTATT
CCAATCGCTGTTCTGTTTGGAGTGTTCCTTTTCCTCGGGGTTTCATCTTTATCCCACATTCAGCTGGTCAAGCGAATCAA
ATTACTTTTTATACCTGCTTCCCACCATCCCGTTGAAAAATTTGTGACGAATGTGAAAACGAGAAAAATGCACCTGTTTA
CGATTGTGCAAGTGTGTTGTGTATGCGCGCTTGTAGCCTTGAAACTCACTGTTGTTGCGCCAGCATTTCCTTTCCTCATT
ATTTGCATGATCCCGCTTCGTAAACTTCTAGAGCGGTTTTTCACCAGTTTAGAGCTAGAGGCGTTAGATACTGAAGTAGA
GGATGTGTATGACAGTGACTTGGATGAATATGACTCCATACATTTTCCTTTCTAA

>Spi_SLC4β (Comp15849)
CGAGGAGATGGAATGGAAGGAGACGGCCCGGTGGATAAAATTCGAAGAGAATGTTGAAGATTCAAACCGTTGGGGAAAGC
CGCATGTGGCCTCGCTCACCTTCCATAGTCTACTTGAACTGCGCAAAGGTCTAGAGAAAGGTACTGTATTGCTGGACTTG
GAGAAATTTGATCTGCCAAGTATTGCTGAAGCAGTAGTAGAAAACATGGTGATTACAGACCAGATAAGTCAGACAGACAG
TAATAAAGTCCTGGCAGCATTGCTTTTACGGCATAGACATCAGTATCAGATGTCTCCTCCTATTGCCCGACGGCCATCAT
CATATAATCTTTTGGCTTTGGCAAGGAAGGACTCCAAACACCCAATAGAAGATTCTGAAAATAAAGTGTTAGAACCTGAA
GAAGGTGAAACGTTGGGTGATGGAGCTATCCGGCTAAAGATTGATGAAAGTGACAGTGATTTTCCTGGCAAGAAATCAG
TCCGTTATCACTGAGTGGTGATGAAAAACAAGATCAGAAGGCAGAAGTGTCCTTTGCACCAGAACCTGCTGTGGTAGAAG
GAGGTTCTGGAAAACAAGAATCAGATGTCAAGTCAAGAATTCCTGAAGGAGCAGAGGCTACTACTGTCCTTGTAGGAACA
CTCGATGAACTCCAATATCCAGTTGTAGCATTTGTTCGTCTCGCCAAAGGGTGCCCCTTGAATATCACAGAAGTGTCCAT
TCCTGTCAGGTTCATGTTTGTGTTGTTGGGTCCAAGCTCGGAGGAAGAGTCTTACTACGAAATTGGTCGGTCCGTGGCCA
CCCTAATGTCAGATCAGGCATTTCATGACATTGCATATCGCGCAGATTCTCGGGAAGACCTCCTGTCAGCTATCAATTGT
TTCTTGGATGACACGGTTGTATTGCCGCCTGGAGAGTGGGATCGTAACGTGTTGCTTCCAATCCTCATTGCTCAAAGCAG
AGCTAGGGCACGAAGAAGAAAGATGGCTAAAGCTGCTCTCGCTGCAGTTGCTGAGGAACAAGAAATAGACCCATTGGAAA
GAACAGGTTCATTCTGTGGTGGCCTTGTGGAAGACATCAAAAGACGAGCTAAAGTTTACCTCAGTGACTTCAAAGATGGA
CTTAATTTGCAGTGTCTCCTAACGTCGATATTCCTTTATTTTTCTGTCTTTGCTCCCAATGTTGCGTTCGGTAGTCTTTT
GGACAAGAAAACTGAAGGATGGCTTGGTGTGTCAGAAGTAATTCTAGCTACTTGCATGTGTGGAATTTTGTTTGGTCTTC
TTGCGGGACAGCCACTGATTATTATTGGCGCTACAGGGCCTGTGTTGGTTTTTGAGCAGACTATTTATGAGTTCTGTAAT
GTTTACAACGTGGAATTTCTTCCATGGCGCTGTTGGATTGGCTTCTGGGTCATGATGATCCTGTTTGGGGTGGTTGCTTT
AGAAGGCTGTTTTCTTGTACGATATTTCACCAGGTTTACAGAAGAAATCTTCGCTTGTCTGATCTCTGCTATCTTCGTCT
ATGAGGCCATACACTTTTTAGAAGTGGTTTTCAAGAAAAATCCTTTGCAGAAAACGTACACAGAATCACCTAAATCTCCT
GAATATGAAGTGAAGGGGCAACCAAACACTGCTCTTCTTTCCACCATCATGGTTCTGGGAACATTCTTCGTTGCCTTTTA

CCTGCGGAAGTTCAGAACAAGCTACTTTTTTGGGAAAAAGGCCAGAAGACTTGTAAGCGACTTTGGCATAGTCATAGCCA
TGGCTTGTATGTTACTCGTGGATGTCTTTCTTTACGATAAAGTCGTCACGCAGAAGATTACCATCGGTGATCCTACGAAA
AGAGGTTTTTTACCAACAAGATACACAGAGAGAGGCTGGTTTATTAACCCTGGAGGGATGAAAAGACCCATGGAACCTGG
CTGGATTTTTGCGGCCATAATTCCAGCCTTGTTCGTGGGAATCTTGCTGTTTATGGAAACTGAACTGACGGGAGTGCTCT
TGAACAAGAAAGAAAACAAACTTTTAAAGGGCGCCGGCTTTAATTTGGACCTAATCGTTATGGGATGTCTGTCGTTTGTT
TGCTCCATGATGGGGCTGCCATGGATGTGCGCGGCGACTGTCCGGTCGGTGTCACACTTAAATGCGCTGTCTATCTGGAG
TACGTCTCAAGCTCCTGGCGTTAAACCACATCTGGTCGAGGTCAAAGAACAGAGGGTTACAAACATTGCCATACACGTGT
TGACAGGGGTTTCCATTCTACTTGCTCCAGCTCTTCATCGTATCCCCGTCGCAGTCATGTTTGGTGTACTGTTCTATCTT
GGTGTTTGTTCCTTGTCTGGTATTCAGCTCGTCGACAGAATAATTATGATGTTTATGCCCCCAAAGTATCACCCAGATGT
GCAATATGTTAGAAAGGTCAAAACCCGAAAGATTCACTCGTACACAATCATCCAAGTCGTCTGCCTGATTCTCCTCATCG
TGGTCAAGTTAAACGTCATCGCTCCGTCATTTCCATTTTTCATCATATGCTTGATTCCTCTTCGCAAAGTTTTGACAAAG
TTTTTTGAACAGAATGAACTCGAAGAGCTTGACAATGAAGAGCTACCTGACGATGATAGCGACCTGGACGAGTACGACAG
CGTGTACATGCCAGTGTAAAGGATACGGACATATTTGGAGAAGAAGTGACCCTTGGAGAACACAGCATTATGGTGATGGC
ATAATTTTGATGATGATCTTCATTGATTCAAACCGTGAAAGGGGTGCTGTTACATATCAATAGTTTCCTTGTAGCTCTTG
TATCAATTTTTTTTTTGAGATGCAGAGTGCAAAAAAGTGTTACACGCAAAGCACTGCAAATTGAATGTGACCCAACATTC
TCACGGTTGAGGCGAAAAGGGACTTCATTCATACAGGTTCTTGCAAGGAGTGCTCCGTGAGGGTCAATCCTTTAAAAAAC
CCTTCCAGAAATCTTGTAGGAGGACCCTCTTTATGGGAAACCCTACTGAAATATCCGGAGGGACTTCAGAAGTGCCCCAA
CACGACCATTTTTTAGAGGTGCCTCGGTCTTTCGTAACTAACTTTGTATAATTGTTCAGCTAGAGATAGAGCGCCTTTCG
AGTGAGTGTCGTAAAACCAAAACCAA
>Spi_SLC4γ (Comp3751)
CCCAATTGCAGCTCTGTTCCTTCACAAGCTCTCTTCTAAGATCATACCTGAATTCTCAAACTCGTACAAATAAGAATCAC
AGAACTGGTCAGAGATGGTCAAAGGTGAGGGAGACACTACAGATAACAAGAGAAAAGGTGCTCTTCCAAAAATCCAGGAG
GGAACATCGGAAAGTAACTATGAAGGTGACCATAGTCATGACGATAGCAGGTTCATGCCATCTGTGGAGGAATGCCGGTA
CCTAAACGACCAGGGGCAGCATATAGACTCAGATAAGGTGCCTCATTATCATCATTACCGAACCATCTCAGAAAGAGAGG
AATTTCAGTCCAGGATTCGAAACGGTGCGGTCTCAATGAAGGACATTGAAGTGCTGCAAGAACGCTTGGAGAGAGACAAC
GATAACAATATTTTGGCAGGAATGTCGGACTCTATCCAAATGGCAGGACTTCGCAAATCTCAACGCAAATTGTCAGGCAT
GCAGACTCTTTACAGCTCAACAGACATCAGCGCTAGTTTGGACAAGTTTCCTGTCTTATATGATCGTAATCCTCACGAGG
CATTTGTTGAGTTGGAAGAACTTAGCAAGCACGGCGAGGAGATGGAATGGAAGGAGACAGCTCGATGGATTAAGTTTGAA
GAGAATGTTGAAGATGGTGACCGCTGGGGAAAGCCACATGTGGCTTCACTAACCTTCCACAGTTTGCTAGAACTACGTAA
AGGCTTAGAAAAAGGGACTGTCTTGTTGGACTTGGAGAAGTTTGATTTACCTAACATCGCTAAGGCTGTGGTAGAGAACA
TGGTGCTCTCAGACCAGCTCAAACAGAAGGACAGTAACAAGGTCCTTACGGCGCTACTGTTGCGACACAGACATCAACAT
CAACGACGTCCTTCATCCAAGAGGAGAAAGAAGTTGGCTGTAGCAGCTGATAACTTGGGATTTAATAGCCTAGCAGAGAC
TGGAAACGGTGTCATTCGAATGAATATGAATGAATCAAATGGTGATGTGCCAATAACTCAGGATAAAGTTGACATTGAAA
CTGCCGGTTTGGATGAAGATGAGGAAAAGCAGGAGTCAGATGTAAAAGATAAAATTCCTCAGGGAGCCGAAGCCACGAAC
GTCCTTGTTGGAACACTGGATGAACTACAGGACCCAGTAATGGCGTTTGTACGGCTTGCTAAAGGAGTCCATCTAGAGGA
AATGTCAGAAGTGTCCATACCCGTTAGGTTCCTGTTTATCATGTTAGGTCCAAGCTCACATGGAGAAATTTATCATCAGA
TTGGGCGCTCTGTGGCCACTCTTATGTCCGACCAGGCTTTTCATGACGTTGCTTACCGCGCTGACTCTCGTGAAGAACTC
CTGCAATCCATCAATGAGTTTCTTGAAGACACTCTAGTTCTGCCACCAGGCAACTGGGATCGAAGCGTTCTTCTTCCCAT
TCTTATTGCTCAAAGTAGAGCCATAGCTCTCAGAAGAAAGCTGGCCAAAGCTGCTTCATCCTCCCATTCGGAAGGAGACC
CTGCTTTGGCTCGCACAGGAACCTTCTTTGGGGGGCTTATCCAGGACGTCAAGAGACGAGGCAAAGTTTACCTCAGCGAT
TTCAGAGATGGTTTCAATCTTCACACTCTCCTGGCATCTCTTTTCCTATATTTCGCTTTATTTGCGACAAATATCGCTTT
TGGAGGACTGTTTGAAGACAAAACTGAAGGCTGGCTTGGTTTGACTGAAGTGATTTTTGCAGCTTGCGCTTGTAGTATCC
TGTTTGGGTTGTTTGGAGGGCAGCCCATAATGATCATTGGCGCCACTGGTCCAATGTTGGTGTTCGAACAAAGTATATAT
GAGTTTTGTAAGGCATACGATGTGGAGTTCCTTACTTGGCGCTGTTGGATCGGGTTCTGGGTAATGATCATCTTGTTCGG
CGTGGTAGCCTTGGAGGGCTGTTTCTTGATCAAATACTTTACGCGTTTTACAGAGGACATCTTTGAACTTCTCATCTCAG
CAATCTTCATCTACGAACCACTTGCACTGCTGATCAAGCTTTTCAAAAAGAATCCACTGAACAGGGTAGGGAGCGGAAAA
TACTCAAGCGTGGACGAAGTCAAAGGAGAACCCAACACAGCTTTGTTGTCCACCATCTTGGTTCTGGGAACGTTTTTCAT
CGCTTTCTACATGCGCAAGCTCAGAACAAGTCACTTCTTTGGAAAGCGGGCCCGAAGACTAGTCAGTGACTCTGGTATTG
TCATCGCTATGTTCATTATGGCTATGATCGATTTTGCCCTCGGTGACAAAGTCATCACCCAGCATATATCCATCAATGAT
CCTGTGACGGAAGGCTTCAAGCCCACGCAACACGATAAACGAGGTTGGTTCATCAATCCTGGAGGCATGAACAAAGAAAT
GTCGATGCCATGGATCTTTGCAGCCATCATTCCTGCAGTTTTTGTTGGTATCTTGCTGTTCATGGAAACGGAAATGACCG
GGGTGCTTTTGAGCAAGAAAGAACACAAGCTTGCCAAAGGTCCAGGGTATAATATGGATATGTGTGTGGTGGGTATGTTA
GCTTTCGGATGTTCCCTGCTCGGGTTGCCATGGATGTGCGCGGATACAGTGCGATCGGCTTCGCACGTGAATGCGCTGTC
CATCTGGAGCACGTCGCATGCTCCTGGAGAGAAGCCGCATCTTATTGAAGTCAAGGAGCAGAGAATCTCGAACATCATAG
TTCATGTCCTGACAGGTTTGTCCATTCTGCTTGCTCCAGGGTTGAATGTTATTCCCATCCCGATTTTCTTCGGCGTCCTC
TTGTACCTGGGTATTGTATCCATGTATGGGCTTCAGATGGTTGACAGGTTCATCATGATGTTCATGCCACGTAAACATCA
CCCGGATGTCGGATACGTCAGGAAGGTGAGAACCGGCAAGATCCATTGCTACACTGTTATTCAAGTACTAGCCTTGGCTT
TCCTGGTCGGAATCAAGTTATCTCCTCTGGCACCATCCTTTCCCTTTTTCATTATCTGCTTGATTCCTCTACGCAAGCTG
TTAACAAGGTTTTATAAAGAGGAAGAACTTGAGGAGCTTGACAATTCAGAATCAGAAGAGGAGGACAGCGACTTTGAACT
ATAATTAGTTACAGCATCTGCCGTATTCTGCGTGACTGGGTGTTGCGTGACTCACTTCACGTCTCTATCATTACGTGACA

CACGAAAATTATTTATTCGACGTGAAGAAGAACGCGATGAAGATACAGCCTGCTATCCAGTCAGACAGAAAGAACCTTTA
ATCCACGTAACACTGAATAATGATCGTACTTAACGATCTTAATTATCACTGGCGATTGCAAAAGTTTATCTTCTGCTTTA
TTGTATATGGAATATGGGTGGAACAAAAATAAAGTGGTATGCACAGAGGCCCACGCGAGGCGAGGAGCGCGTATTCCCTC
GCGCTTGTCCCAACCTTCATGCACGCCTCGGGTTTGCTTCGCTAGCTCTTCCCTTATCTTCACTCCCCTGAAAAACGCAA
AACAAAATTATGACTGTTCTTCAACCTTAGAGCTTCAACCTTCAGCTTCAAATAATTTGATAAATTGTGACATCTTCATC
TCTACTTGTTTACAATTAGCAATCAGTGAATCCTCTACACTCACGTGACATCTTCAATCACTGCTCGCTTACAGTTGGCT
ATCAGTGAATTCTCAACACTTACTTGTTACTTTATGAATTAGTAGACAATTTAAAGCCTTCCGGCTTTTACATTTTTGTA
GCCTTTGGTACATCCAGGATTTATTCAGGTATTCAGTTTTACTACAATGTTACCTTAAACCAAGATTTTGTAGATAAAAC
AGCTCTACATGTCAATATTTTTTGTTAAGGTTATATTTTGTAAGACTTAAAGTTATTGAACACTGTAAAATAACTCCAAA
TTATATTTCGTATATTTGCAATATTGGCGTACTACCGGAAAATTGGTGTAAAATTGTTAATTATGTCATGGAATTTAATT
TTGGGAATGGAATCTTAAAACAAGTAGCATCCTTCACTTGATGAATTTGTATATTCTCGTTGCT

>Spi_SLC4δ (Contig3506)

GTGGAATCATGGAAATTCGGCAACCGAGTTACGATAGTATTCCCGGACCCGTTGACGCAGGAATATCCAGTAGAAATGGC
TACTTTGGAGAACTCGACTATCAAAATCATCGTCAGGTTTATGTAGGAGTTCATATTCCTCTGCGACAAAGGAAACACCA
CCACCATCAGCGCCATCATCACCGACGCAGACACAGGTCCTTACTCGAATCCAAAGATGAACTCTCGTCAGATGCAGGGA
CACCATCATTGCCAACAGACAAAGTGCGCTTCATTCTTGGTGGAGAAGAAGCTGATCAAGAGCAACACAAAATGTTCTCA
GAATTGCTGGAATATCATTGTGATAAAGATGGTAATGAAGAGTGGAGGGAAATGGCTCGGTGGTTAAAATTTGAGGAGGA
TGTAGAAGAAGGTGGAAATCGTTGGAGTAAGCCGCATGTTGGTACCCTTTCACTTTACAGTCTGTTTGAGTTGAGAAGTG
CACTTTTACGTGGAACAGTACTTTTGGACATGAATGCAACCAATTTATCTCAAATTGCTGATCTTGTCCTTGATGATCTG
GTGAATAAAAATCAATTGGATCAGGACAACAGGCAGCAAGTACGTGATGCAATCTTAAAGAAGAAGCGTCACCAGGGAAA
CAAAACACAAAAGAAGAAGAGCTCCAACCCAATGAGTAGCTTTGGATCATTTGCAAAGAAAAGCTCGACCGCTGGCTTCA
CTGACATGTTCAAAAGATCTGATTCTTCCCAGAAGACAACTGCTCCAAAGAATTTAGAGGTGGTGAACGAGGTAGACACA
AATGATGGAGAGTTCCCACATTTACCATCAGCTGCAGACTTGGTGGGAAAAGGGAAAGAGAGTGGTGTTAATGGTAACGC
AGGTCTTGACCAGACAGACTCTGAGACTTGCTTAGAAGCGTTTGATGCCAACTTTATGAAAAAGATTCCTCAGGGCGCTA
AAGCTTCCAATGTCTTGGTTGGTGAGATGGATATCATCAAGAAGCCAGTGACAGCATTTGTACGTTTGGAGAATGCATCA
CTCTTAGGTGACCTCACTGAAGTTCCTGTGCCATCAAAGTTTATATTTCTCATGTTAGGACCATCTGGTACACCAGGAAG
GTACATGGAAGTTGGACGTGCTATTGGGACACTGATGTCTGATGAGGTTTTTCATGAGGTTGCCTACAAAGCACAATGTC
GTGAGGATTTGCTGGCTGGAATTGATGAATTTTTAATGCAGGTAACAGTGTTGCCACCTGGTGAATGGGATCCCTCCATT
CGCATTGAGCCTCCAGATGCTGTTCCAGCGCAGGGAAATCGTCTTGAGGTTGTGAAGTCTGGAGGTGGTGGAGAAATGGA
GGAACATGATCCTGCAGGAGACAGAGAAGAACAACTTCAGAAAACAGGAAGGTTATGTGGTGGTCTCATTGCGGACATCC
GACGTCGTGCTCCCTGGTACTGGAGTGATTTTAAAGATGCTCTGAACCCTCAGTGTATTGCATCAATTATCTTCATTTAC
TTTGCTTGTCTCACACCAATTATTACATTTGGGGGTTTGATGGGGACAAAGACAGGGAAGAACATGGCTGCTATGGAGCA
AATTCTTGCTGGTGGAATTGGTGGAGTCCTTTTCTCCTTATTTGGTGGTCAACCACTTATTATTCTGGGTGCAACTGGGC
CTATGTTAGTGTTTGAAGAAATTCTGTATACATTCTGTGATAAATTTGGCCTTGACTACATGCCATTCCGTTTATGGATT
GGTATATGGACGATGCTGTACTGTTTTGTCCTTGTGGTAACAGATGCCAGTGCCCTTGTGCGTTACTTCACCCGTTTCAC
AGAAGAGTCATTTGCCACCCTAATTGCATTGATCTTCATTGTTGAGGGTTTCAAGAAGTTGTTCCATGTATTGGATGATA
GCCCAGTGAGTAAAGGTTACATAACTAGTCACAGTTGTTTATGCTATCCGGGAGAAGAAGATTCTTCCGGTCACCACATG
GCAGACAGCTCGTCTTTTGTCAACAAAACACCACTGGACTATCCAATCGGAGATTGTGTAAGGTATGGTGGTCAGCTTGT
GGGTGAGGCATGTCACTCTAATGTGTTTTTCTTGTCGGTCATCCTATCTCTCGGAACCTTTTTCCTGGCAGTTACACTGA
AGGAATTCCGTAGCAGCAGCTATTTCCCAACAAAGGTCCGCTCTGTAATCTCTGATTTTGCTATCCTGATTAGTATCATG
GTAATGGTTGGTGTAGACTTGGCATTTGGCGTTAACACACCAAAGCTTGACATCCCATTGAAATTTGAGCCAACAAACAG
TAAAGAAAGAGGTTGGATAATCCCTCCTCTGGGTAAAAACCCTGTCTGGACCATCCCTGCTGCAGCAATCCCAGCTTTAC
TAGCCACTATCTTAGTTTTCATGGATCAGCAAATCACAGCACTGATTGTAAACAGACGAGAACACAAGCTCAAGAAAGGA
GCAGGTTATCATCTTGATCTTCTACTTGTGGCTCTTATCATTGGTATATGTTCATTGCTGGGTTTGCCCTGGGTTGTAGC
AGCCACTGTTCTGTCAGTCGGTCATGTGCAGAGTTTGTTTGTAGAATCTCAGTGCACAGCTCCTGGAGAGAAAGCACAAT
TTCTTGGTGTGAGGGAGCAACGTATCACTGGAACATTCATTTTCATCTTGATTGGCCTGACTGTGTTTATTGCTCCCATA
CTTAAGTTTGTTCCAATGCCTGTTCTTTTTGGTCTCTTCTTCTACATGGGATTCTCAGCACTTAGGGGACTGCAGTTCTT
TGAAAGGTTAAAGATCATGTTTATGCCTGTGAAGCATCAACCTGATTTGATGTTTCTGCGACAAGTTCCTCTTAAGAGAA
TCCATATCTTCACACTGATACAGCTACTTTGTTTGGGTATACTGTGGCTCATCAAATCCACGGATGCTGCCCTGATCTTC
CCTGTTATGGTATTGATGTTGGTTGCTGTGCGTAAAGTAATGGAGAAGATTTTCAACCTCCATGAACTTGAAGTGTTGGA
TGACTTGATGCCAGAGAGCATCAAAAAGCAGCAAGCTGATGAGGCAGCAAAAAAGAAGGATGATGACTATGATTCCGATG
ATGATTTAGATGATGAGGATGGTTGTAATCATTACGATGAAACTGATGGTAAAAAGGCCCTCAGTGATGGAAAGGAGTTA
AATGGCACTGCAAATGTTGTAGCACCAATGAATATCTCAGAGGAGATGTGCAGGAGCTCACTGTGGAAACAGTTTGTCAA
GGATGGAACAACAGCAAAAAATAGAAAGAACAAACCTAGTGATAAATCCAAGCATAAACACAAACACCATCACAAAAACA
GGCACCATCGCCACAAGAGAGATCGTGAAGGATCAGCCGATGAAGAGGGACTGTGGATGCTCGAGAAGAAGAAGGAAGAT
GGTGAAGATGCAGGACCTGATGTTGAAATGGGAAGAAAGCTCTCTACAATAAATGAGGACAAGCAGGATATTGTTATTGA
CATGGAGGAGATACAGAAGTCTTTGCTTGAGTTGAATGAAATTGGCAACACTGTGACTGATGAAGGAGCTGACAAGCAAA
CTCCGCGTGATTCAGAAAATCCTGTTTAGAATGTTTCCTGCGTGAATTCTGGCATTCATTTTATACCGGTATTGTTGATC
ATATGAGAACATATTAGTATGCATACATGATTAAGAGGCAGTTGTAATTGCATAAAATGGCACACTCTGAGGTTGCTACA
GACAGCACAGCAACCCAATTTATTTAATTAATATCTCCAGTAACACACACAAGTCAAAGTGCAGACACATACATGATATG

ATAACTGTGGACTTTCAATTTTTTTAAATTTTAGAACTCAGATTTCTCTTCAGGCATGATGATATGTATTGACTGCATTA
ATTGATGAAAAAACTCTGATCGGTAACTAAAGGGTAGATTAAAACATAGTGGTGAAAAAGCTGTATAGAAACGAGGTCTT
TTGATGGGGCAAGTAACTTGCGGTTAAAAAAAAAACTGCATAAAATCTATTGTAAGAGTTGTAATTGAATTAGAATTATC
TTGGAAATTTAATTAATAATCTGTTGAAAAAGATTTGATAATTTGCAGTTTTGTTCACATGATCATGTCTGATAAGAAAT
GTCTGTATTGAACTTCACTGATGTGTGGCTCACAAGATGGTATAAAAAAAAATTCAGTTGGAAAAAAGCATTCAGTTGGA
AAACAGCTCTGTTAAGATTGATTGAGAATTCATGTTCTTGAAAACTACACTTAAGGTAGATAAAGCAGTTTAGAAAAAGG
CTTGTGTAGAGAATTTCATTTTGTTGTTGTTTAAAACTGTAACAGGTTAGCTTTGAAGTTTAAATGGTGAACAGTAACAT
TGGATTGTATGTTATATTTAATGAATAAGCCATCTAGGTGGTAAGATAAAATTATGTCCATAAAACAAATAAACAAACAC
ACTAACAAACAAGCTTCTCCAGCAAAGGTGACAAACAATATCATGTAGCAGTTTGTGCTAATAAATTTCAGTATTTTTGT
ATTTTTAGTAATTAAGGCATAGCAGAGGCGGGTCCTCTTAAGAATTGTTTCTAGCTTTTGTTAATTTGGCCTTTTAATCA
TGGTTTGCACTCTCTCGCAAGCCTAGTGACTATGCAAACTCAGACCTCACAGGTGGGCTCATATTTTTTCACTTCCCTGG
GATGGTATTAGTTTCACTTCATTTCAAGGTTTTCTTTCAGTTACTCAGCCAGGATTTAATGACCAAAACTCAACAGACTC
AATCCTGTAATAAAGCAATCTATTTTGGCACTTAATTTAAAATGAAACAAGTTTGGGACAATCTTGTATTTGTGAATTTC
AGAAGTTATTTATTCCAGCTTAATGCGATAACTAAAGGGGAACCGTCCAATTTTTTAACTTTGTAGTAGAACATTTTCTG
TGTTTGTGGGTGTACTTTCAATTTTTGCATTTGATTTTCCAGTGGTGGTCTTACCACTTAAGTTTGAATCCTTCTTGACA
GTAATTTACACTTAATCAAATATTTGCTTTTGTAACTAAATGTACTGATTTTTTATGGTTTTCAGTTAGTGATAACATCA
ATCAGTTTGATTGTGTGATTATGTTTACGTTATATTTGAGTAGTGCTATTTAGAGAGCACTGTAGGTTAAAGCTGTAAAT
TTTTGATAAGAGATATTTGACAGTATATTATGGAAGTTGTTCAGGAAGTAGTTGCCAATCTCTATGTTACACCTGATGTT
GTTAAATTTATTCTTGGTTCTAATTTCACCATTATTTGTTGCTGCGTATAGTAATTGATGAATGAACTACACCTCATACA
TGAGCTTTGAACCAAGAATAAAGCTTGACAATGACAGACTCGTTTCTTCAGGTTTATATCTTCATTTTGGTTTTAAATTG
TTTAAATCAGTTGGAATAGAACATTTATTTTTTTTATTTTCCTCAATTTCAGATGATCATGAAAACCAATCTGATCGCTT
AGAATTTTAAACCTTTATGAATTTTTAACCATACTGTAAACAAGTTTTATTCACTGCACATATTCCAAACTTTTAGATAT
GGATGTAAAAAGTATTGTAATGTTAGAATATTTATGGTCCAAACACACATTAAGTTAAAAATTAGTTTGTAATTAGTAAG
GTTACCTTAATAAAAATGCATGTCTTGTGTTGACTGGAACAATTTTTTGTAATTCTCAAGACTCAAATATCTGATCACAA
TAGCTTGCATTTTGAGAACCAATACAATGTCTTAGTTAATAATTATGTTTTTTCGTTGTTGTTGTTGTTGTT

>Spi_SLC4ε (comp32210)

GTTTTTTTTTTGTCTTAAAATGTCAGTTTTAAAAGAGTTACAAGCTGCCCGGCAGCAAGTCGATGGTCATGCATGTACTT
CCTCGAGTATACAAAGTTCTTTCGGCCTTGCACTTTCCTGAAACACAACTTTTACGGCAGTCTTAGTGGTTTTGATAAAA
AGGTTAAATCTATAAAATATATAAAACAACATGCAAAGACCTTTCAATCAACCTAAAGGTGTTGGAAAAGAGGACGAAAA
TGGTTCTACCCACAGGAAGGAGTGTAGACGGCTTTGATATTGCTAATATCGACGAAAGGAATCTAAACAACAGCCAAGGAG
ACGTGACGCCGGCAATGGAAATGTTGAAGTTCACAGACTGTGACGACGATGAAAGTGAAATAGAAACGGTTGAGATAATT
AATTTGAATTCTTGTGGACAAGGGTTTGACAACGAGACAGTGGATGAATCATATTTAGTTGAAGAAGATGGTGTTCCTGG
TACTTCTCTAATGCTGTCTAGCCACCAGAAACTCCCTTCAAAAAACTTCTGCGCTGAAATCAGAGCAGCCAAGGATGTTG
AAACTTTCCTTGAACGACCAATATTACTTCTTGGACTGCAGGAGACAAGTCTGCAGGGAATTATTGATGCAATGTTGAAG
AAGCTGATAACTGTAATTGGTGGAGAGGACATTGATTTTGAAGAAGCTAGACTGGCATTTTTCACCCATGACTCTGTTCA
CTGCTTGTCTAAAATAATCCAAGGAACAGAAATATCTGAAGGAGGAGGCTGGGAAGAAACTCAGAATTGGCTTATTGCAC
TGGGAGAACTACCATCTGTACAGCAAAGCCACGTGGCCTTTGCACAACTGAGACATCCTGTAAACTTGGGAAGAACACTC
GAAGAGACTCATTTGGTTGTCTTGGTGCTTGCTCCTAGTAAAGCTAAAAGCACAAAGAACTCACTGGAAACAGGAAGAAC
TTTTGCAACATTATTGTCTGACATAGAGATAAGACAGCTGCTCATTGAAGCAGAAACAGAAGAGGAATTCAAGAAAGTAC
TGTCAGATCGCAAAGGGATGCTATCTTCACAATTATTGCCAAAACGCAAATCTACCATAAGGCAGTTTGGTTCCCAACCT
AAGTCCTCCTTTATCCATCAAAGAGATGATGAAAATGAAAAGAGATCAGAGTTGTTCTCATTTGGAAGGGGGGTCATACG
TGACTTTCGAAGGAGATGGCCACATTATTTATCTGATTTTAAAGATGGAATAAGAGGTCATCGGACCATTCCTAAAGTGA
TCTCCACAACACTTTTCTTATACTTTGCCTGTATTCTGCCATCAATTGCATTTGGTGTTCTCAACAGCCGCAACACTAGT
GGAAAAATTGATGTTCTGAAAGTTATCATTTCACAATCTGTGGGAGGAATTTTGTTTGCTCTGTTTGGAGGACAGCCTTT
AATTGTTTTACTTACCACAGCTCCACTAGCACTTTACATCAAAGTTATTTACAACATTGCGGCAGACTTTGATTTGGATT
TCTTTGCCCTATATTCTTGCACTGGCCTTTGGAATTCATTCTTCCTGTTTATCTACTCTACATTTGGTCTTTCCCAGATA
ATGAAGTGGTCAACAAGATCGACAGAGGAAATTTTTGCTTTGTTCATCTCACTTGCATTTACAGTAGATGCCATCAGTAG
CATCATCAAAGAATACTCAAGTCCTCACTGTACAAGTTTAAGTTCCTGTTGGAACTCATCATCTTGTGTTGTTGGCCAGG
GAATTGCTAATAACTTCACTCATGATCAGGTCCCAGATGTATCAGTTTGTAGCAGAGAGGGAGGTCTCTCCTATGCTGTT
ACAAGCTTAGGAACAGTTTGGATTGGTGTAGCACTCTACAATTTTCGCAAGAGCCCATTTCTTGATGCTGGAAAGAGAGA
GGCACTGGCTGATTATGCACTTGTGGTGGCCGTACTTGTCATGTCTTTTATTGCTTCATTTTTTGTGCGAGATATTGATA
ATTACAAGTTTGAATCAAGCAGCACATTTTCATTTACTGTTGCTCCATTGCACAAGCTCACTTGGGGTGCAGTGTTTGCA
GGACTTGGGTTAGGGTTCTCCCTTTCTCTGTTGTTTTTCATGGATCAGAATATTTCAAGTGCCATGGTTAATAATCCTGG
AAACAGACTGAAGAAAGGCAGTGCATATCATTGGGATTTACTTGTTGTAGCTGTAGTCAATGGCTTTCTATCCATTTTTG
GCCTGCCATGGGTACATGCTGCCCTACCTCACTCGCCATTTCATGTCAGGGCACTGGCAGATGTTGAAGAGAGAGTAGAC
AGAGGGCATGTGTTTGAAATCATTGTCAAAGTTAGAGAAACAAGACTGACTGGGTTTCTCTCCAGCACTCTCATTGCATT
ATCACTGTTAATGTTACCCATTCCTCTCACTCTAATCCCCACACCTGTCCTTGATGGCCTGTTTCTTTTTATGGCCATGA
CTTCCTTATATGGTAACCAGATGTTTGAAAGAGCATTGCTGTTGGTCACAGAACAGGCTGCCTACCCACCAAACCACTAC
ATTCGACATGTGCCTCAGAGAAAGATGCACCTGTACACTGCTCTGCAGTTCTTACAGCTTGGTATTCTATGTGGATTTGG
ATTTGCCCCATTGCCATATCTTAAGATGGTGTTTCCTGTTCTACTCATGTTTATATTGCCAATCAGGCATCTTCTTGTTC

CCAAACTGATCTCTTCAAAGTATCTTGAGGCTTTAGATGCTCACTTGTGAAAAGTGAATTATTCAGCTTTTTGAAAACAA
ACAGACAAATCAGAATAGTTCTTCAAGCATGCGGATGTTAGAACTATAGTATGTTTTTCTG
>Spi_SLC26α (Comp171)
GTTTACGTTTCCTTCAGTTTCATGATCAGCTAATATGTTCCTTCGACTTCTTTGGCTCGAAGATGCTGTAAACTAACCAG
TTAGGAACACTCGCCTTAAGTATACAGAAGACAAATGTGTGGAACGTAAAAATGCTGTGGGAACAAGTGAAATCGTGCAA
GGATATTCGTGCAGAGAAAACAGGATGCGAGAGCTTACCTCACCGGCTCGCGATATTTCGTCAGCTGTTTAACCTGTCGA
TCATCAGCGAGTTGTTTTGAAACAAAATCTTTAAAATTTATGAAAAGTGAACTGAAAGTCACACATCAAGGACTATGAAG
GATTTAGGAGAATCGGAATGTCCGCCGTTGATAACTCGTCAACGCCGAAGACGATACAGAATCAATTTGTCTCGACATGT
TTACAATGAACAAGAATTCACTGAGTCGTTTATCAATCAAGAGGAATACACAGCGAGCTCTGAATTTGAACCCTCTTCCT
TTGCAAAAACATTAAGGCGAAATTTCCTGCCATCTAGATGTACGTGTAAAGAAATCTTACAATCATGGTTTCCCATAGTG
GAATGGCTACCAGCCTATAATGTTCGGCGTGACTTAGCGCACGATATAGCAGGAGGGATAACCGTGGCGATCATGCATAT
TCCTCAAGGTCTGGCATATGCATTGCTAGCATCACTTCCAGCTGTTACAGGACTGTACACAGCTTTTGTTCCTATTCTTG
TTTATATGGCAATGGGGACATCCAGGCATATATCTTTAGGAACATTTGCTGTGGTTTGTCTAATGGTTGGTCACGTTGTT
GACAGAGAAGTAGAGCATTCTTTGTCACCCACGCCAACAGCACCAGCTACTACCAACCAGCCCCCTGGTGGCTCAACTGG
TGTAGTATTACAGAATTCAATTTATAAGCATGAGTCTGATGGCAGTTCGCTGGATGAACAGGACTTGCTGGATGAAAAGA
AGTTAGAAGTTGCCGTGGCACTTTCAATGCTTGTTGGTCTTCTACAGCTTTTAATGGGTCTATTCAAGCTTGGTTTTGTG
GCAGTTTATTTATCTGATCCTATCATTAGCGGTTTCACAACTGGTGCAGCAATTCTGGTTTTCACCAGTCAGGTGAAACA
CATCCTGGGATTAGAGGTTCCACGATATTCTGGAGCATTCGCTGTAGTTAAGACCTATTTATTTATGTTCAAGAATATTA
CTCTTTCTGTACCAGGATCAGTTATCACTGGGGTTGTCTGTATCCTGATCCTGATTGCTCTGAAGTACATCAGTGAGAAG
CTGAAGCACAAAATGAAATTTCCTATACCAGCTGAATTAATAGTGGTTGTTCTCGGGACAGTGGTGTCCTATTTTGTTGG
ATTAAATGAAAAATTTCAAGTTTCAGTTCTGCATGACATTCCTAAGGGGTTACGAGTACCATCTGCTCCGTCTTTCATGC
TAATGGGCAATATCGTGACAGATGCCATAGTGATCTCCATTGTTATATTTGCTACAAACATATCTTTGGCCAAAACATTT
GCCAAGAGGAATAATTATGTAATTGATTCCAATCAGGAACTGATTGCTTGTGGATCTGCAAATGTACTTGGTTCCTTTTT
TTCTTGTTTCCCTGTGAGTGGTTCACTTTCCAGGTCAGTGATTCAAGAGAGCATTGCAAGAACACAGCTCTGCAGCATCC
CAGTTGTTGTAGTCATTATCATGGTGTTGCTTTTTATAGCACCTCTTTTCTATCACTTGCCAAAGGCGATACTGGCTGCT
GTAGTAGTTGTTGCACTAAAGGGGTTGTTTCGACAGTTTAGCAGACTGGTACAGTTGTGGAGAATGTGTAAGCCTGATGC
TGTTGTTTGGTTTGCTGCTTGGTTAGGAGTGGTACTGCTTGGTATTGACATTGGATTAGGAGTTGGTGTCATCATGGCAT
TGTTAGTGGTAATCTGGAAGTCATCCAGACCTCCTGCTTCCCTTCTTGGTCAGATACCCAACACTGGAATTTACAGAGAC
ATCCAAAGGATACCTTCAGCACAGCCAATTCCTGGAATAAAAATTTTCAGATTTGAGTCTGCTATGTTTTACGCCAATTC
AGAATACTTTCGGAGCACTTTGATAGAAATGACTGGAGTTGATCCACAGAATCCAAACAAACGCAGCCGCCTCGGTAGCT
CAGCAGTGCGCTATAGACACCGTGAGGAGGATACTGGGGGCCCGGCAATAGAAATAACAAGGACTGTTGCTGTTTCAATA
AACAGGGCTGTGGAGAATGGGGATGTGGAGGTTTCGCTTAATGATCTTGGTAATCAGCGGAGTCCCTCAGGATACGATCC
AATCCCTACTCATGCCATCATAATTGACGCTAGCACTTTCAATTTTATTGACACCCAAGGTGTGAACACTCTGTTGCAGC
TTGGTGTAGAATATGAAAAGATTGGAGTAAAATTCTATTTGGCGCACTGTCGATATCACATACGAGAAATGTTAGAGAAA
GCTGGCTTTACAGCAAGAATAGGAACAGAACATTTGTTTGTGTCTGTACATGACGCAGTGACACACGCTGTTGGAATTCA
CTCTGAGGATGCTTCCTATTCGTCTCCTGTGATGAGCGACACTGAATCAGCATTGGGGGCTGGACCTTCTAATCAACAGG
TAGAAGAATGAATTACACTCGACGATTTGAAATGACTTGTGAAGCTAATCGAAGAACATAAGAAATCCCACACAAGGGAT
TTTAACTCATTTACTATTTTGCAACATGTAGATACCCAGAAGACGATTTCAATCATAACAAACGAAGAGTGTCCGATTTT
TATTTCTCAGAATATTTCCAGTGAAATGAATGAATTTGCCATCTAGCTGAGATATCAAAACCTCTCACATAAGACGGTTT
TTTTTTTTTCTGAGTTTAAAGTTCTACTCATTTTACGAGATATATTCTTTTTTCACATTTTCACTCGCGTGGTAAGCAGT
CATATGAATTTATTGAAACAAAAGCTCGAAAATTTAGCTCCAGCGGGTTTATAATTTCGCTTGAACTCGGACGTTGATTT
AAAAGGAAAATGGAAGGTTTGCACACAGGATAGAACCAGTCAATTATAACTAGGAGTGACTGGCTTCTAATTTCTCCATA
CAGTGTCACCCTTGAATCAAGGATAAAGC
>Spi_SLC26β (Contig313)
TCATAACTAACCTGCTTGAGGTAGGTGCAAGTGCCACGTGTGAAATTCGCGTTCAAGCGTTTCCCTACTATCATCTTTTG
CCACTGGCTTTATTCTCATGTGAAGTGAAAAAGTTAGACCTCGAGTTGTCCCTCCCAGTGGCAGATCTAGGTGAGGGGCA
CGCTCCCCCCTCTTATTTTGGGTAAAAAATTAAACGCAGAGGGAAGAAAAGCCGGCAGCTAAACACACACAAACGAAAAC
ACGCCCTCTCCCCCCCCCTCCAGCTCAAGCTCTGGATCCGCCACTGATAGCCCTTTATGTACTTTTCAAATTAAGTACAA
ATGAATATTTTATCGAAAAATTAGAATATTTTGGAACACATTCACTCCATGAATCGTAATATTATATATATATGTATGTATG
TATGTGAATTTCGCTTTCTACAAAATCCCTCTGACCTTAAAAAAAACCAAAATTATTCGTTAAGTAATGTGGAAATTGGG
AGGCTAAGAGCGCGATTCCCTCCCATTCCGAAATATCCACCAAAAATTTGAATTTTTCAGTGTGTAAACCTTAATTATTT
TTTTGGGAGACACTCATTTAGAAAGAAAGAAAAAAACTCAGCCAGGATCAGCATTAAAAACATGTCTCCTTGTCTACGTC
TTCTCCCCAAGCTGCGTGGTCACGTGATCGTATGGCAGATACAACAGCGTCATGAATGGATGGAAACATCGCTTGTTGTG
GAATGGCCTGTGTGGAGGCCTCCCCAGAGGAGCTTGTGAACTTCTCAATGAGGAGTGAAGAACAACCAGCCAGGTATATG
TGAATTCCAGCTTTCTTGAACTCTGATAGAAGGGATGGAAGAACATGAAGACCAACAGAATCGATGTAGGTAAAGCTGCT
GCAATCAATGACGACAGTGTGAATACGGTTTTTCGGCTCATCAACTTTAACAGCAGTTGTTGTTGAAGTGCCTCCTTTTT
CCTGAGGTTTCGGTTTAGAACCAGGGTTCCCTCTGTTACTGTTTCCTTTGTCTTCATTCCGGAGAAGATCATTGTTTTCC
TCGTCGTTTTTCTGTTTGACCTCTTCTCCCGCTTTCTGTTCAGCACTGGGATCTAATCCAGTGGTACTAACCAGCGCGCT
TCTGAAGCGCTCCGCGTTACCAAAGTAAAGTGGGGACTCAAGTCTCAGTACTTTCACTCCAGGGACCTCGAAGGCCGTGG
GACATTGTTTGACGTCACGATATAACTCAGTTGCTGTTACTCTGCCAAGGATGGTGTAGCTGGACCTGGAAAGGTGAGA

ACGACTGCAAATAACGCGCAGATCACTCCTACACCCAATCCTTTATCAACTCCCAGCAGAATGACACCAAAGCAAGAGAA
AAACCATACAAACGCATCAGGTTTGTAAATTCCCCACAAAGCCCGTAGCCTTGAAAATTGTTTGAGTAGGCCAATGAGAT
TGGCGATCACAACTGCAGCTAGGATAGCCTTTGGGAGATGGAAGAAAAGTGGAGCAATGAACAGCAGAACCATAAGGACC
AAGGCGACCACAACCACGCTGCACAGCTGAGTGTTTGCAAGGTTTTCCTGTACCGCTGTACGCGCTAAAGCGTTGCAGAT
CGGGAAACACGAAAAAAAAGAGCCAGCGAGGTTTCCTGCTCCATATGCAATCAGCTCCTGGTTCGGGTCGACTGAGTAAC
CACGCTTCTTAGCAAACATCTTGGAAATTGAAATGTTTGTGGCAAATATCACCACTGCTATGACAAAGGCGTCTGAGAAA
ATGTTCGAAATCCGGGATAAAGATGGTACACTCAAAGGAGGAAGCCCCTTTGGTATATCTCCTATGATTTGTGTACCATA
ATTTAAACTGATTTTCGCACCATATGATATAAGAGTTCCGACAACAACCACAAGAAGTTCAGCAGGGATGGGAACTTTCA
TTTTCTGCTTGTATCTCTCGTTCACTTGTTTCAATCCAATTAACACAATAAGACAGAGCACGCCCGTGATAATGGCTCCA
ACGTTCGCTGAGGGAATGTTTTTCAGCATATGAATAAAGATCTTTACTGCGGCAAACGCTCCGCTGATCTGGGGAACTTT
CACTCCCAGTATATGCTTCAATTGGCTGTTGACAACGAGCACCGCTGACCCTGTGGTGAAACCACTGATCAATGGTTCTG
ACAAGAACGTCGCCAGAAATCCCATCTTCACTGCTCCCATGATAATCTGGATGATACCAACGAGTAAGGAGAGCGAAAGG
GCGACTTCCATTTTGGCCGACATTTCCGGTGTCCATAACGCGGTGCCTTCTGTCGGGGAAGGACTGGACATCGAGTCATT
GCCCGGTGTAGGAGTGGGCGCTAATGGCATGGAACCCACTTCACGCTCAACAACTTGTGCCACCATCAAACATATGACTG
CGAAACTACCTACAGACAAGTGCCGCGATGTGCCCATGATCATATAAACCAATACTGGGATAATGGCGGTGTAGAGTCCC
GTGACCGGCGGAAGGGAAGCCAGCATGGCGAATGCGAGTCCTTGAGGGACATGCATAACTGCTACTGTAACTCCTCCATT
TAAATCAGGAAACAAATCTTCTTTCACGTTGTATTTCGGTAACCAGCGAATAATCGGCATGAGCCCAGCTAGAAAAGAGA
TCAAACCCTTCCACGAACACGAACAGGGAAAGAGCTTTGGATGAACAACGCGCCAGTAATAGTTCTTCTTTGCTGAGTCG
TCCTTGGTACTCAATGGAAAGCGATCGCGTTTTGTTGCAGTGAAAAAGTTTTCATCGTAAACATTTCTGGCAATATCTAT
TTCAGGGCTTTGGCGGTGAATTGACCGCTCTCCGGGTGAAGATTCCATTTTATTCAAATCTTCAGAGGAGAGTTTTACCA
TCCAATTCTTATTCAATC
>Spi_SLC26g (comp55357)
TCATGTGAAAACGCCGTGTTCAAAGTTGTATCGCTTCTTTAAATCAACACATTTGCTACACCTTTCAGTTTCCATCACAT
TGACTTTTTTTTATGGATTGACGTTATAATTCAAATAGCAGACCTTAAAAAGTATACTGACCAATCTCAGAAAAGGCGTT
CGGGTCGAGAAAAAAGTTCGAATTTTGGAGAAATGCGGTTAGTTACACATTCCTGTTACGTAACTTTTCGCTCTTTTAAG
ACACGGAAGACAGTAACAAACAAAGAAGCATTATTTAGAATAAGTTAACGAAGGAATTAACTGATTTAAAAAGGGGGTCA
CATCGAGAAGACAGTGGTATGATCCAATTACGTAAGGGAGAGGCACGCGCCAAAAAGAGAAATGTAGTTTGATGGGGAAA
AAAAAGATCAGCTGATTTCCGCCTGTCTTCACGTGAAAAAGGATGATTTTCAAACTGCCTTTATTATAGTGGACTCTTAT
CGGTGATTACTGTAAGCAACAATTTCATTCTGTAACGTTCGTCAGCTTTGGTGCGGTGAAATGGCGTCGGAAGAAACTAG
CAAAAAGTTGCAGACTTTCTCGGTGAGTACTAAGGATAAAAACGCCCACGATCTATTCTGAGGGTGACTTCACATCATTG
TAGAATTTAAAAGAATTTAGTAGAATGAAAGTTTTCTTTTCAGGGAGGAAAAGCGTTCTCGTTAAATGCTCGTGTAGAAT
TATGCGATGCCGCTTTAAATTGATGATGGAAGACGAATATTATCGATTTCCACTCGTTTTTTTCCTTTGCTCGTTGAATT
CTGAACAAATTGAACTCATCTCCTATCTAACTCTTGGATTTTAATGTATGCAAACATTGCCTCGCTGATTGTTTAGTTAC
CGCAGGCAACCATGTGAATACATGAATAGCTCAATTGGCCATGATTGATTGGTGGTTTTTTGAAATGACCTGCTTGCCGT
ACGCTAAAACAAAATGCAGAAATCAAAACTTATCGAACCTCAGGCGATTGCCGCGTCATTTCACTGTAACCAGTTCAGAA
TTCTGCCGATGCCGTTCAGCATAACCTCGGTTCGAACGCCCGTCAACGTGTCATTTTAACATGAGTTGCTCTGCTTTACA
GGGGAAATGCAGGGAATTCTGTTCTCCGGGACCGGTGAAGGTGTTTGTGAAGAAGCGCTTTCCAATCGCGTCTTGGCTGC
CAGAATATAATCTCAGGAAGCTACAGTGCGACATGATTGCCGGCTTAACTGTCGGTCTTATGGTTGTTCCTCAAGGTCTA
GCCTACGCTCAACTGGCCGGACTGCCACAACAATACGGACTATATTCGGCGTTCATGGGTTGTTTCCTCTACTGTATTCT
GGGTACGAGTAAAGATATTACTCTAGGTCCCACTGCGATTATGTCTTTAGTGGTATCTGCGTACGGAAACCCGAAATAC
CTCATTATGTTGTCGCATTAACTTTATATACTGGAATAATCTTACTCGCTATGGGCTTTCTTCGGTTGGGATTCGTTGTA
AATTTTATTTCTATTCCAATCGTGAGCGGGTTTACCTCCGCAGCGACAATAATCATCGCCTTTAGTCAGCTGAAAGATTT
GTTTGGATTACAAAAAATTCCTCGTAAATTCGCCCAGAACGTGTACTTCACCTTCAAGAATATCGGTCAGACAAACAAAT
GGGACCTAACATTGGGGTTATTATGTATAATAATTCTAATAACCCTGAGGAAGGTTGGACGACTCGAATGGGTGAAGCGC
AAGGACTCGAGCGACTCCAGATGGTTGAAGGCAGCAAAGAAGACTGTTTGGCTCATTTCAATCAGTAGAAATGCTCTCAT
AATTTTGATCGCTGCAGTGGTTTCTTCGTCTCTTTATCAGCATGGCCATAAAGATATTTTTACTTTGCCATCGCGTATTA
AACCGGGTCTTCCACTTATGCAGGTGCCTGCTTTGAGCTTTCAAGTTGGCAATGCTACAAGAAGCACCTTGGAGGTGTTT
AAGGATCTTGGACCTGGTCTGGCTGTGGTTCCCCTTATTGGCTTTCTCGAGAGCATTGCAATTGCCAAGGCCTTTGCACG
TAAAAATCGCTACACTGTTGATGCCAGCCAGGAATTGATTGCCCTTGGTGTTGCCAATTGTCTCAGCTCATTTGTATCCT
CCTATCCAGTGACTGGAAGCTTCTCTAGGACTGCTGTGAATGCACAAAGTGGAGTGGCAACACCTGCAGGAGGAATTTTC
ACAGGTGCTATTGTTCTCTTAGCCCTTGGACTCCTGACAGACTCCTTTAAGTATATTCCAAAAGCATCTCTTGCAGCACT
GATCATGTCCTCAGTGATAACCATGATTGAATATCACATTGTGCCCAACATCTGGAAAGTGCGGCGCATAGACTTGGTGC
CTTTGGCTATCACCTTCTTTGGCTGCTTCTATGACATTGAAGTTGGAATTTTGGCAGGTATTGCAGTTGCCTTATGCATT
CTCCTGTATAATGTGGTGTGGCCCCCCATTACAAGAATAGCTAGAGGAGACTATGTGCTTCTAAAAATCAACGGAAATCT
CAACTATCCTGGCGTTGAACATCTGACCAATGAGATTCAAGAAGTCGCAGCGATGGAACCTTCCCCTCCTGGTATTGTAA
TCGATTTTTCTCTGGTCACAAGCATTGACTTCACAGTCACTCAAGCACTTCTGACAATCCTGGAAGATATGGAAAACAAG
AGAATTCCTATATTCTTCTCTGGTGTTCAGGACAATGTGCGAAATATGATGATGAACTCTGGGATTGATTCAGGCATTAT
TAATCAAGGCACACAATCTGTCATAGACTCAATTAATTCTTTGGAAATAGTGGAACAAAATTGAATCAATATTAAAGCAT
CAATAGTGAATAGAGTAGTAAAGTTTCAGGAAACTATTTTGGTTACAGATTAGGCAAATGAAACAGTAAACTTTGATGAT
GACAATGACAATGAGAACATTTTATAGGGAAAGCTTTTAGGATTATTCATCCACAAAAAATACCATTAAAATTACCAATA

CATTTACTGGGTGAAATTTTTCTGTGCTACACACAATTTGAAAGTTTGCAGCATTGTAAGGAGATATTTTCAAACTATAG
CATACTTTCTTTTTCTGTTGTTGGACCTCAAATTTTTCCCTACTGCAGGCAAATTACTCAAATGTTAATTCATGTATTAG
TCCTTTTTTTTGCCTTTCCAAGAGAATCCAACTACAAGTCTTGTATAGAGCAAACTCCATTGTGTATGTAGGTCACATTG
TGTCTGAAAGGACTGCAAGAGTTTAGGGGATACTGTAGACCTTCACAGGTGTCCTTTGCCCAAACATCTTTCAATGCACT
ATTTGAAGGATAACCACCCTTACCTACATATCAATGGAAAGGTTATGAAATGCACTATTTGGCAAAAATTCTTTTGTGCA
ACCAAAATCAGGAGATGTTTAGGTAATTGAAATTCAAGGACCAGGATATTTTGCCACAAAAAACTCTTGGGGAGTCATTT
ATATTGACATGTTATAGTTGGCTATTCACACCTTGGTGGGCGAAAGAATTAATTTGGAAAATATTGTGTCTGTTTATAAC
TTGTGTGTGTTTCAATATTGTACCAAGTTTTTCTTTGAAACTATAATTAAAATAATAAGGCTTAGTTTGACATGAAAAAA
ATTGAAAAAACTTGGCCACCTTAGGTCTACTGTATCCTTAAAAAGTTCAATATTTGTATTCATTTTAGAAAAGTAAAGGT
TCCATGATCATAGTTAACCATTATGTAGGTCAGTCAGAGTGATATATATTAAGATTAATGTTAAATTTCATTAGATTAAC
ACAGAAAATCATTCTATATTATTTAAAGACATTTTACCTTGTGATTTTGCTATTAATTCCATGCATTAGTTATTTATTCA
TTGTGCTTAGATTCAGAATTTCTCTTCAAGTGTAAGTTAAAACAAGCACCCCAGACAA

Table S2. Name of the different *S. pistillata* clones found in EST libraries and corresponding homologs from the genomes of *A. digitifera* and *N. vectensis*.

nom	*S. pistillata*	*A. digitfera*	*N. vectensis*
**SLC4α**	**Contig6766636 (GenBank: KJ829357)**	adi_v1.05797	NVE15348
**SLC4β**	**Comp15849 (GenBank: KJ829358)**	adi_v1.04799	NVE11220
**SLC4γ**	**Comp3751 (GenBank: KJ829359)**	adi_v1.04793	Not found
**SLC4δ**	**Contig3506 (GenBank: KJ829360)**	adi_v1.09009	NVE6522
**SLC4ε**	**comp32210 (GenBank: KJ829361)**	adi_EST_assem_6901	NVE24532
**SLC26α**	**Comp171 (GenBank: KJ829362)**	adi_v1.08419	NVE15856
**SLC26β**	**Contig313 (GenBank: KJ829363)**	adi_v1.07620	NVE8226
**SLC26γ**	**comp55357 (GenBank: KJ829364)**	adi_v1.07348	NVE23992

Table S3 : Percent identities between the anthozoan SLC4 members. Numbers represent ‘‘% identity’’ of the deduced amino-acid sequences of the different BATs. Comparisons were made using LaserGene’s DNAStar software and Clustal W with gap inclusions

	**1**	**2**	**3**	**4**	**5**	**6**	**7**	**8**	**9**	**10**	**11**	**12**	**13**	**14**
**1: SpiSLC4α**	***	47.0	45.4	35.4	21.6	67.9	45.3	44.9	35.5	22.7	54.3	47.2	36.4	22.6
**2: SpiSLC4β**		***	68.8	39.9	22.6	50.1	81.3	67.9	37.9	21.9	48.4	66.4	34.8	22.7
**3: SpiSLC4γ**			***	35.7	22.4	46.3	62.2	80.0	34.9	21.8	45.2	61.7	35.1	19.4
**4: SpiSLC4δ**				***	23.0	37.9	37.3	38.8	72.5	22.6	37.6	41.3	63.4	23.0
**5: SpiSLC4ε**					***	23.9	22.6	24.0	23.3	75.4	21.3	23.4	22.5	55.1
**6: AdiSLC4α**						***	49.2	47.3	35.6	23.7	54.6	49.0	37.5	24.9
**7 AdiSLC4β**							***	67.3	36.6	21.8	46.8	65.5	35.7	23.0
**8: AdiSLC4γ**								***	35.6	23.1	46.0	61.5	35.5	20.6
**9: AdiSLC4δ**									***	24.7	35.1	38.7	58.4	23.4
**10: AdiSLC4ε**										***	22.2	23.0	21.3	53.9
**11: NveSLC4α**											***	48.3	34.5	20.8
**12: NveSLC4β**												***	36.3	22.1
**13: NveSLC4δ**													***	23.9
**14: NveSLC4ε**														***

File S4. genome sequences of SpiBATs

>scaffold_134 450527 454290
ATGACCTCTATCGAAAATATTCCGATTGATCAATCCACTTGTTCTCCGTATGAAGGATTTGTGGAATTGGAGGAACTAGT
CAGTTGTGATGATGAACTGATTTGGAAAGAAAGTGCGCGGTGGATAAAGCTCGAAGAAGACGTCGATGAATGTGCCGATC
GATGGGGGAAACCTCATGTGCCGTGTTTGACATATCGGAGCCTTAGAGAGGTTGAAACGAATCTTCAAAATGGAGTGTGT
TTGTTGGATCTCAATAGAGATACTCTGCCTTCCATTTGGGATGCAATTATTGATGAAATCAAAACGCTGAAAAAGRTGGA
AGATAGTGAATGCCTCGTAGTGAAAACTCTTCTAATGACAAGACATGAACATCATCATCAGCATAAAAGTGGAAAACGAG
TTCAAAACTTCGCCAGGCGCCGGTCACGGATCATGCCTAATGGACAGCGCTCCAGGACGAATGTTTCGGAAGATGTCCAC
AATTTAAGAGAAAGGTATGAGGATTGGCACCAAGCTAAGTGGGTGACCTTTTTTTACTCTTTAACTGGTAGGCACTTTAT
TACTGGTCCAAGGGTGACAGCAAGTTTTTTCCCCGAAATCTTGACGGTTGAGGGAACAAAGCTCGCTGTTTCTGTTTAAG
CCAGTCATTAAGTGTTTTGTTATACCTCCCAAGATACCTACTCGAAACGGGCTGGCACTCAAATTTTCCGAAGTTACAAG
GTGCACAGACTGATTACATGAGAGCGGAGAGCTCAGTTTGTTTTTTCCCCTTTAGAGTTTTGTCGTTTTGCACGTCTTTG
AGTTTGGTGGAATAATAATCTTATTTGACTCTTTGAAAAAAATGTCTACTGTCAATTAAAATATAATACTAAATACAATC
AAGAGATTGGTACACTTCCTTGGGTACCCTTACTTCCAAGGACGTATGCTTAAGAGATTTCATATAACCACTGTTTTGAC
GAAACAAAGCCACGAAGAAATTTTGAATTAAAACGAATGTCCAAGGAATTTTTTTTTCGATCTGAAGTGTCATCGTATCT
TGTTTATATCATCGCAATCTTTATTACATGCTGGACTCATGAAATTTCCTTTCACATGGAATTCTAAGGCCAGGATTGAC
CGACTTAAAAAAGCAATGCTAATTATTTATTTATACGATGATCCTTCTTTGATTTCAGACCGCAGCTTAGTCTCGAACAA
AACATGTTACCCTCCATTGACAGCAAACAAGATGTCAGCGAGTTTCCAGCAAACGTAGCAGTGAGTTTCAGCCCTGATCC
CGTTGAAGGGTTCGTCGAACAGTTTGACGATCACAACAAAAAATCAGATCTGAGAGGGAGACTTCCGGAAAATTCTCAAG
CAACCACTGTTTTGGTTGGGGCTCATAATGGCTTGTCTTGCACCGTCAGTGCTTTTATAAGACTCGCCAAAGGATGCGAA
CTGAGTAACTTAGCTGAAGTACAGATTCCTATCAGGTTTATTTTTATCCTGGTGGGACCTAACGATGACAGGATAGACTA
TCATGAAGTCGGGCGATCTTTTGCAACTCTTATGGCAGATAAACTCTTCCTGGAATCTGCTTATCTGGCCAAGGTAACTT
CCTCAGTTCTTAATCGATCAGCCTTCCTTTTTGCGTAGTCTTATACTTAAAATAATTAAAGCGCGTTGAATGCTATTCAA
GGAGTCAAAAAATAAGTATGTACCCATAAATTTCACGATAAAACTGGTTCTTCAATAAACATGATATCATGTTTTACTCC
TGGAAAGTCAGTTGATTACGATCTTCAGTAACACATAACTTGGAATGGCTTTTTCAAAGTGGTTACGAGCTAGTTCGCTA
ACAATTAAGTGATTTTGACTCGAAGGGGAACTGAAAAATTTGATTGTCTATGTTTCAGCGTCAGCAGGATCTTCTGGATT
CTCTATTCAGCTTTATGGGAGACAGCCTCATAATTCCTCCAGGAGACTGGGATCGAAATCTACTAAGTCAAACAATACCC
ATCTTAGCCAGTCAAACCAAGGAATTAGCCTTAAGGAGACGAAGCGGAGGAATACAAAGCAAGGACAAACCAAATGAAAG
ATTTCGCGAGGGTTTTCAAGAAAATGAAGTGTGTGATGACCCTTTGTCTAGAACGGGAAAATTTTGTGGTGGACTTATAC
GAGACGTTAAAAGGCGATTTCCTTTCTACTTGAGTGATTTCAAAGACGCTTTGGACGTTCATTGTCTACCAACAATCATC
TTTGTGTATTTCGCTTGCCTCGCTCCTACAATCGCCTTCGGCGGTTTGCTGAGCGAGAAAACCAACGCATGGATGGGAGT
TTCAGAGATGATATTTGCAACAGCATTATCAGGAGTTCTATTTGGCCTCTTTGCAGGACAGCCATTGATCATAATAGGAG
CTACGGGTCCATTACTCATTTTCGAGAAGAGTATATTTCAATTGAGCGAGCGATTTGAGATTGAGTTTCTCCCTTGGCGA
GCTTGGGTTGGAATATGGGTGATGGTGATATGTTTTATCATTGTCGCCGTTGAGGGATGTTTTCTCATCAAATATTTTAC
ACGTTTTACAGAAGAAATATTTGCCTTAGTGATATCTATCATTTTTATTTACTACGCATTTAATTATGTGAGACATATCT
TTGATCTGTATCCTTTGAACGCAGACGTCGCTACTACTCTGTTGGTTCCAAAAAAATTCGACCAATCCTACGAAAGGAAAA
TCTGTAGGGAATCAGTCAACGGCGAACACAGCTCTGCTCACCACAGTTTGATGCTGGGAACCTTCTTTGTCGCTTACAC
CTTGAGAAAGTTACATCACAGTCATTTCTTTGGACCCAAAGCTCGTCGCATAGTCAGTGATTTTGGGGTTTTCATCGCTA
TTGTTTCCATGGTGACAGTTGGTTGGTTGACAGAAGGTGTGTACACTCAAAAGTTATCAGTCCCGGATGGTTTTTCTGTT
TCCTCACCCAACAGAAATAGCTGGCTTATTAACCCCATGGGAGAACAACAGCACATGAGTGCTGTCGCCATTTCTGGAGC
TTTCATTCCCGCAGTTCTTGTCAGCATCTTGATTTTCATGGAAATTGAATTCACCGGGATTATTCTGGACAAAAAAGAAC
ACAAATTGAAGAAAGGCGTGGGTTACAACCTAGATCTCTTTGTTCTGGGACTACTAGTTGGCCTGTGCTCAGTTCTGGGC
CTTCCGTGGATGTGTGCCACCCCTGTTCATACATTGTCTCACTTTCACGCCCTGACAGTGCTTAGTACTAACCATGCACC
GGGGGAACATCCTCGTCTGGTACAAGTCAGAGAACAACGCCTCACGAACATTGTGATCCACCTTCTTATTGGCTTCACTG
TTCTTCTATCGCCGGTGATGCGTCTTATTCCAATCGCTGTTCTGTTTGGAGTGTTCCTTTTCCTCGGGGTTTCATCTTTA
TCCCACATTCAGCTGGTCAAGCGAATCAAATTACTTTTTATACCTGCTTCCCACCATCCCGTTGAAAAATTTGTGACGAA
TGTGAAAACGAGAAAAATGCACCTGTTTACGATTGTGCAAGTGTGTTGTGTATGCGCGCTTGTAGCCTTGAAACTCACTG
TTGTTGCGCCAGCATTTCCTTTCCTCATTATTTGCATGATCCCGCTTCGTAAACTTCTAGAGCGGTTTTTCACCAGTTTA
GAGCTAGAGGCGTTAGATACTGAAGTAGAGGATGTGTATGACAGTGACTTGGATGAATATGACTCCATACATTTTCCTTT
CTA
>scaffold_266 314488 324383
CGAGGAGATGGAATGGAAGGAGACGGCCCGGTGGATAAAATTYGAAGAGAATGTTGAAGATTCAAACCGTTGGGGAAAGC
CGCATGTGGCCTCGCTCACCTTCCATAGTCTACTTGAACTGCGCAAAGGTCTAGAGAAAGGTATGATAACTCCAGCTCGT
TAACCAGAATTTGAAGTTGAAAGGTCCTGCCTTTTTGTCATTAACTGAGATAAAACTTCCCAAATGAGCGGTCTAGTAAC
CTAAATAAAATACTTGCTCATTCAAACAAGCTAAAATTTTATCTATAAACAGAAGGCAAATCATATTGCACTAACTGGTC
TCATTYGAAGAGTTCCTTATTAAGAGAGAAAAAAAGTGAGGCACATTGGCTGTTATGTTGAAGAGAACCACCAGATTCAC
TGTTTATGAGGCTCATTGATGCTTTATGAAGTTACCAGTACAATAAGAGAAAATAGGCTTAACCAGTAACTCTTTGATAT
GAAAGRGTGACCAGCATCTATTTTCTCTATACGATGTCACCCCTCAATCAAACATTAAGGTCAGGAGAATACAGATGATG
ATCATCAACTAAAGAAGCTTTGATTGTTAAACAAATTCTGCTTGTCAGCTCCTTAGGAAATGTATAGAGAACAGTATGGA

GAATATGCATACTGATGTTAGGGTGTAAAGAGTTAAGAATTACCCTCTTTTTGATGTCATAGGTACTGTATTGCTGGACT
TRGAGAAATTTGATCTGCCAAGTATTGCTGAAGCAGTAGTAGAAAACATGGTGATTACAGACCAGATAAGTCAGACAGAC
AGTAATAAAGTCCTGGCAGCATTGCTTTTACGGCATAGACATCAGTATCAGATGTCTCCTCCTATTGCCCGACGGCCATC
ATCRTATAATCTTTTGGCTTTGGCAAGGAAGGACTCCAAACACCCAATAGAAGATTCTGAAAATAAAGTGTTAGAACCTG
AAGAAGGTGAAACGTTGGGTGATGGAGCTATCCGGCTAAAGATTGATGAAAGTGACAGTGATTTTCCTGGGCAAGAAATC
AGTCCGGTAAGTCACACAAGCCTTGCCATTGCCAGCAGGGTTTATTGTTGAAATTGGTCAAAGAGGGCCCAGTAGACSCC
ATTTTCTTGTTTTAGGGGAGCTTTYTCAAGATAGRAAGCWCAAGAAAGAAGGTTCCCTAAAATGTGATTGCTTCTGGTTG
TTAAAGGGGCAATGTTGTCTTTCTCTTTAGAGTTAATTAACTAATATTTCTTCGATAGTTATCACTGAGTGGTGATGAAA
AACAAGATCAGAAGGCAGAAGTGTCCTTTGCACCAGAACCTGCTGTGGTAGAAGGAGGTTCTGGAAAACAAGAATCAGAT
GTCAAGTCAAGAATTCCTGAAGGAGCAGAGGCTACTACTGTCCTTGTAGGAACACTCGATGAACTCCAATATCCAGTTGT
AGCATTTGTTCGTCTCGCCAAAGGGTGCCCCTTGAATATCACAGAAGTGTCCATTCCTGTCAGGTTCATGTTTGTGTTGT
TGGGTCCAAGCTCGGAGGAAGAGTCTTACTACGAAATTGGTCGGTCCGTGGCCACCCTAATGTCAGATCAGGTCTGTTAT
ATTCAGATTCATTTAATGAGCTGAATTATGTATTTTGATTGAYTGTTACCTATGATCTACTATTAGAGGACAGACGTAGA
GATGATGTCACCAGTTACTCCTTAACTTTCAAGATCTGATCGTTAATTCTCCCCTCTAGCTGCTACACATTTCCTTGTAA
AGGAAAAGAGAATTTGGTGTTAGATCAAGATAACAATTTCTACCTGATAAGCTTGAGTATTCTCGTTACCTATTTTTCTG
GATAATGTAGGGATATTATAGAGAGAAGTTTCGTGTTAATCACCTATGTTGCCATGGGTCTGTACAGTAATGGCTTACAG
AAAACGTCAAAATGTAGCAAGAACATCAGTGACACACGGGGCTGGGCTTCTTGTGCCACCTTTTTGTTCTCATCACATTC
TAAGGNNNNNNNNNNNNNNNNNNNNNNNNNNNNNNNNNNNNNNNNNNNNNNNNNNNNNNNNNNNNNNNNNNNNNNNNN
NNNNNNNNNNNNNNNNNNNNNNNNNNNNNNNNNNNNNNNNNNNNNNNNNNNNNNNNNNNNNNNNNNNNNNNNNNNNNNNN
NNNNNNNNNNNNNNNNNNNNNNNNNNNNNNNNNNNNNNNNNNNNNNNNNNNNNNNNNNNNNNNNNNNNNNNNNNNNNNNN
NNNNNNNNNNNNNNNNNNNNNNNNNNNNNNNNNNNNNNNNNNNNNNNNNNNNNNNNNNNNNNNNNNNNNNNNNNNNNNNN
NNNNNNNNNNNNNNNNNNNNNNNNNNNNNNNNNNNNNNNNNNNNNNNNNNNNNNNNNNNNNNNNNNNNNNNNNNNNNNNN
NNNNNNNNNNNNNNNNNNNNNNNNNNNNNNNNNNNNNNNNNNNNNNNNNNNNNNNNNNNNNNNNNNNNNNNNNNNNNNNN
NNNNNNNNNNNNNNNNNNNNNNNNNNNNNNNNNNNNNNNNNNNNNNNNNNNNNNNNNNNNNNNNNNNNNNNNNNNNNNNN
NNNNNNNNNNNNNNNNNNNNNNNNNNNNNNNNNNNNNNNNNNNNNNNNNNNNNNNNNNNNNNNNNNNNNNNNNNNNNNNN
NNNNNNNNNNNNNNNNNNNNNNNNNNNNNNNNNNNNNNNNNNNNNNNNNNNNNNNNNNNNNNNNNNNNNNNNNNNNNNNN
NNNNNNNNNNNNNNNNNNNNNNNNNNNNNNNNNNNNNNNNNNNNNNNNNNNNNNNNNNNNNNNNNNNNNNNNNNNNNNNN
NNNNNNNNNNNNNNNNNNNNNNNNNNNNNNNNNNNNNNNNNNNNNNNNNNNNNNNNNNNNNNNNNNNNNNNNNNNNNNNN
NNNNNNNNNNNNNNNNNNNNNNNNNNNNNNNNNNNNNNNNNNNNNNNNNNNNNNNNNNNNNNNNNNNNNNNNNNNNNNNN
NNNNNNNNNNNNNNNNNNNNNNNNNNNNNNNNNNNNNNNNNNNNNNNNNNNNNNNNNNNNNNNNNNNNNNNNNNNNNNNN
NNTATTATCGTAACGGTGGCCAATTTACCATTTCAACTTTGTTGATATATTCAAATTATTTCGTTACCCCCACCGACGCA
GCCCCAAACGGTTTCTTTAGAAACTATCAGCCTTTATTTATTGGTAGCCTGAGTTAGTTTTTACAATAACTATGTCACAA
TGAATACATATATATATTCTTGTAAGAAGTTTAAAAATTTTTTCCAGGTGTAAAACGGACAACAAGAACCTGCATTTCAT
AAGTAGGCAGTCAAATTTGATCGTATACTTTTTTAAAACGCCAAAATTCTCCGTAAGATCGTTTAGGGCTAGAGAATGCT
TGTCGCAAATATTGCTTGCCAATTTAGGAAGAAGTGTTTTTGTGTTCTTGAACACGTTAGTGCAGATGGCGGTGAGCGAA
ACCAGTTTTGTTCTTGTGTAATTAAACCTAGTTTAAGCTGCAGTCATATTTTGCTTTCAAAAGTTTCACGAACTTTAACA
CTRTATATAATGGAAAAAGGGTAGATATAGAGAGAGGATMTACRCAGTTAGAAGTAATTGTTTTGTGTGGTTTTCATGTT
TCAGGCATTTCATGACATTGCATATCGCGCAGATTCTCGGGAAGACCTCCTGTCAGCTATCAATTGTTTCTTGGATGACA
CGGTTGTATTGCCGCCTGGAGAGTGGGATCGTAACGTGTTGCTTCCAATCCTCATTGCTCAAAGCAGAGCTAKGGCACGA
AGAAGAAAGATGGCTAAAGCTGGTAAGGACAAATTTGGTGGATGTTTGTGTTTGATTCAGTGTGACTAAAAGATCTCGAC
GACAGGCGAAAATTTTGTGAACAGACAATGTTTAGGCCGGTAAGGTAATACTCTCAACGAAGTGTCTACTAGTTACAGGG
TGGCTGGGATTTTGAAGTTCTTGACGATAAGATGTTAGGATACTAGAGCGCATTTTTTTAAAGTGTCGTAATACCAAAAC
TATTGTAATTACAAGAGCCAATCAGAACAAGGTAAATTATAACAAGAACCAGTAGGAATTTAAATTAAGAACAAGCCAAC
CGCTTGATTAATTGAGAAAGTGGCACGAGGTTTCTTGAATAATCACAGAGCGGTACAAGGCAACACAAAGCGCGGGAAAA
CGCGAATGGTGAGCTCGTGGTKGGTTTTAGTTTGAATCTGATTGGTTGAGAAGTTGGCGCGAGTTTTTTGGACCAATCAC
AGAGCGAAGTAAGGCGACAACAACGCAATCCCGAATTATTTTCGACTCTGAATTAAAAATTGCTCTRTATCGTCGTTTAC
AGCTCTCGCTGCAGTTGCTGAGGAACAAGAAATAGACCCATTGGAAAGAACAGGTTCATTCTGTGGTGGCCTTGTGGAAG
ACATCAAAAGACGAGCTAAAGTTTACCTCAGTGACTTCAAAGATGGACTTAATTTGCAGTGTCTCCTAACGTCGATATTC
CTTTATTTTTCTGTCTTTGCTCTTTTGGACAAGAAAACTACCTCAGTGACTTCAAAGATGGACTTAATTTGCAGTGTCTC
CTAACGTCGATATTCCTTTATTTTTCTGTCTTTGCTCCCAATGTTGCGTTCGGTAGTCTTTTGGACAAGAAAACTGATGG
ATGGCTTGGTGTGTCAGAAGTAATTCTAGCTACTTGCATGTGTGGAATTTTGTTTGGTCTTCTTGCGGGACAGCCACTGA
TTATTATTGGCGCTACAGGGCCTGTGTTGGTTTTTGAGCAGACTATTTATGAGGCAAGTGTACGGCAACATCTGTTTACT
TTCTCTATTCTGTCTTCATTTCGTGATCCACGGATTGACAAAGAAAAAAACCAAAAAAAAAAAAAAACCGAGAGCAGGACG
GAAATTTCAAATTCTCTTTCCATTAAAATGAGACACGACTGCCTATTCCATGCGCCATCGGTAGCCGCTTATGGTAAAAG
TTATGGGAACCCCTGCATCATATGCTTGGATGSAGAATGGAATGGTGAATTGAGTAACGAGTTTGTTGTTTTTATTTTAC
ATCTTTTCTTTAGTTCTGTAATGTTTACAASGTGGAATTTCTTCCATGGCGCTGTTGGATTGGCTTCTGGGTCATGATGA
TCCTGTTTGGGGTGGTTGCTTTAGAAGGCTGTTTTCTTGTACGATATTTCACCAGGTTTACAGAAGAAATCTTCGCTTGT
CTGATCTCTGCTATCTTCGTCTATGAGGCCATACACTTTTTAGAAGTGGTAAGATATTTACRGAAATTTAGTTTTACTTA
TTATAACTGATTTTTTAGTATTAMGTTTACGTGTGTAATGAACTAAATTATTCGCTGTAGGTTTTCAAGAAAAATCCTTT
GCAGAAAACGTACACAGAATCACCTAAATCTCCTGAATATGAAGTGAAGGGGCAACCAAACACTGCTCTTCTTTCCACCA

TCATGGTTCTGGGAACATTCTTCGTTGCCTTTTACCTGCGGAAGTTCAGAACAAGCTACTTTTTTGGGAAAAAGGTTTGT
AGTGATCACAACTAGCGTGTGGTGTATATTCCCTCTCAAAGTTGAAAARATGTAAGGCACCTAGGKCCGGTTATYTTWTT
KAAAAAAACCGCGTTCTAATCTATCTTGAATTTAGCTCAATCTTTAATCTGAACATTCAGTTTTGTAAACASTGTCTAGA
GGACCGAAAGCTAGCTGTGGAAGAACATTTATTTAATGAAAAAACCTCRTATAGAGAATGCGTAAGGCCCAGTGCTTGCG
TAAAACTGCAATTTGGGTKAGACCTCGTGTAAATAACCGTYCTTTAATGTTATGCAGCCTTGTRATACCTTTTAATTACT
CAAGCTCGATTCTGAAACTCAGGTTGCTATATGTTAGCCAGATTCTGTCGATTGAAATTTAGCTTAGAATCCGTAAGTTT
CAAGTTTAAAATTTGAGTGGAGGTTGACTTGGTATATAACTCCCATAACCATATAGTAAATTCTTGCTTCCAGCCGTTAT
ATGTTTTCTTTGGGATGAGTGGAGAGAATTTTGCGTTCCCTCAAGAYAAAACCGTCCAGCTGTGTGGTTTCTTTCTCCTC
GCAACCTTTTTTCGAGAGCAATACTCAATTGAGTGTCGAAGGTTGTTGGTTTTACTTTTCTTTGCTTTGTAATTGGCTTA
GAAAACTCCCGCTATTCGGCTCTCAAYCAATCAAATTCAAATATAAGACTAATCGCGTCTTGATCAAAACCGTCTTCCCG
CGCTTCATGCAGTTCACTCGTTTTAAACGTGAGTCGTCATTGGCCCCTTGTAAGATATCGATTGTTTTGATTGGCCGTTG
CGTATTCTTTGCCTTTGGTGTCCATTACTCAATCGGAGAAGCTRCTGGATTTAAATGGAGCTAAGCACAGGATGTCGGGA
TCCTTAGAGCTCAATAAGACTCGACTTTTTTTTCTCAGGCCAGAAGACTTGTAAGCGACTTTGGCATAGTCATAGCCATG
GCTTGTATGTTACTCGTGGATGTCTTTCTTTACGATAAAGTCGTCACGCAGAAGATTACCATTGGTGATCCTACGAAAAG
AGGTTTTTTACCAACAAGATACACAGAGAGAGGCTGGTTTATTAACCCTGGAGGGATGAAAAGACCCATGGAACCTGGCT
GGATTTTTGCGGCCATAATTCCAGCCTTGTTCGTGGGAATCTTGCTGTTTATGGAAACTGAACTGACGGGAGTGCTCTTG
AACAAGAAAGAAAACAAACTTTTAAAGGGCGCCGGCTTTAATTTGGACCTAATCGTTATGGGATGTCTGTCGTTTGTTTG
CTCCATGATGGGGCTGCCATGGATGTGCGCGGCGACTGTCCGGTCGGTGTCACACTTAAATGCGCTGTCTATCTGGAGTA
CGTCTCAAGCTCCTGGCGTTAAACCACATCTGGTCGAGGTCAAAGAACAGAGGGTTACAAACATTGCCATACACGTGTTG
ACAGGTCAGTTCAATGGGTTTTGTGAACGAATTTTAACAGCGTGTCGAAAGTTATGGGGTCACGTTGGTTTTCTCTACTT
TGCTCTGTGATGGGGTTTAGAGAATTTGCGCCACGTTTTAACCAATCAGATGTAGTACCAGCGACTTGGGTAAAGGGGTA
AACATCTTAAGAAGCTGTAGTGCTGCGTCGGTTGAGGCTGGGGTTCTACCAAATGATTTAGTTTAATCAACTGAGTCGGT
AATGTAAATGGGCTTCCGTAAAGGGTTTCTAAAGCTGTCGTTTCGAGCCTTAGCCCTTTGTCAGAGCCAGCTCTTCGTTA
GCTCTTCGAARGGTTGCGATTGAAACTTCAAACTGCCTACATACACACACAAAAAAGAAATAAAAATTACCAAAATATGA
AAAATAAAATAGGATTTGCGGTGCACATTCCACCATGTGGCTCTTCTTGTCCACTGTTTCCAATTCGAATTGGAATTTAG
AATGTTGATAAACCAAAGAATTTTGTATTGGCGACTTGGTTATGACTGTTTTCCCGCGCGACGAGCATATGGCTCGTTTT
TTCTTCGAGTTTTCATTGGCTCCTAGTGATTCTTCCTTTGTTCTGATTGGCTGTTATGACAAATTACGACACGTGGCGTG
GTTTTTCTTTAAGTTTTCATTGGCCCCTTGAAATTTTTCCTTTGTTCTGATTGGTTGTAACGGACGTTTTGGTCGATTGC
AGGGGTTTCCATTCTACTTGCTCCAGCTCTTCATCGTATCCCCGTCGCAGTCATGTTTGGTGTACTGTTCTATCTTGGTG
TTTGTTCCTTGTCTGGTATTCAGCTCGTCGACAGAATAATTATGATGTTTATGCCCCCAAAGTATCACCCAGATGTGCAA
TATGTTAGAAAGGTAAGCTGCTACAGACACAGCGATCGAACACAGGACATGCGCAAGGGAATTAATTTCGTTCAGCCGTG
CGCCAATTTCCCGCGCAAAATTTCAAAGGAAAACATGAGAAACAATAGCTGTCACGCAAAATAAGCAATAGAAAATGGAA
ACATGCATAGAAATCTGAAAAGGAAACAAAAAAATTACGGGTTCCCTCCTTATTTATCATTTTGTTTTGAGCTTTACCGA
GAAAGCTTCGCGGTTTTCAAACACCACGACCTACTCGCGCTCGTGTCTGTTTTCATGATAACTTGATTTTGACACCCTTA
AAACACGTCACCTACCTCTACAAACTCGCAAGTGCACAGACGTTTGGCCGCCTTGCTGCTGCTACGATACGTACAGCAGT
TTTTTCGATAGTGACTTTGACGTTGATGAACGATATGATAAACGATGTTGATTTTGAATACTAGCACACGCGAGSCGTTA
TTCAGCTGCAGCAGCTAGCAGACAGGACTCGTTCAAAACTGTAGCGTCTTCATTGCTCTGTAAACTTACCATGTGCGCAA
TAAATGGATGATACGTAAACAAAAAGCGAAAGATAGCGTGCGCCCGCGTTGATAGCGCGCCTATCTTCCAATGTCAAACG
AGAAAAGTTCATGTGCATTTATTCGTGATTCGTACCATTTTTTTCCTTAGGTCAAAACCCGAAAGATTCACTCGTACACA
ATCATCCAAGTCGTCTGCCTGATTCTCCTCATCGTGGTCAAGTTAAACGTCATCGCTCCGTCATTTCCATTTTTCATCAT
ATGCTTGATTCCTCTTCGCAAAGTTTTGACAAAGTTTTTTGAACAGAATGAACTCGAAGAGGTACGTCAATTTTTTATTC
CTTTATCCCAAGATGGGATTGTTAATTCTCCCCTTTAGCTRCCACACATTTCCTTGTAAATTAGTTACGAGAATTTGGTG
TTAGATCTACTTGATRAGTTTGAGTATTGTCGTTACCTTTTTGCTGGATAATGTATCGATATTGTAAGGTAGTGTAGAAA
AATGTTCGTCTCACTTCGTGCGAGACAAACTGGTCGACCAAAATGAACATTCATATTGTCGCCTACAGAAAGGTACTGAA
TTTGGAATCTGTCGATTATGATACATAAAGTTATACTTAACTTAGACATCGGACGTGTTTCTTGACTTTCTTCATTTGAT
ACGTGCACTTTTCACAGCGGCAGTCACATAGCTTTGTGCTTATCGTGTGAAAACCCTGAGACGTTTGTTATGCAGTGACT
CAATAACTCGTTATGTGGCAAGCTAACTGAAGTTGATTTAGTCTCAGAGGGAAATGGCCCGTAAGTGCCCCTGGATAGGA
TGCTAATCCATCACAGAGCCCCCTCCCCGTCCCCCCCCCCCTCCTCAGAGTATCTTTTCGATTCAAAGACGCGTTAACCT
CCAGGCCATTGCATCTCCCGGAGCCAACTCGAAATTCTAAAATCATTTTCCTGACAGCTTGACAATGAAGAGCTACCTGA
CGATGATAGCGACCTGGACGAGTACGACAGCGTGTACATGCCAGTGTAAAGGATACGGACATATTTGGAGAAGAAGTGAC
CCTTGGAGAACACAGCATTATGGTGATGGCATAATTTTGATGATGATCTTCATTGATTCAAACCGTGAAAGGGGTGCTGT
TACATATCAATAGTTTCCTTGTAGCTCTTGTATCAWTTTTTTTTTTGAGATGCAGAGTGCAAAAAAGTGTTACACGCAAA
GCACTGCAAATTGAATGTGACCCAACATTCTCACGGTTGAGGCGAAAAGGGACTTCATTCATACAGGTTCTTGCAAGGAG
TGCTCCGTGAGGGTCAATCCTTTAAAAAACCCTTCCAGAAATCTTGTAGGAGGACCCTCTTTATGGGAAACCCTACTGAA
ATATCCGGAGGGACTTCAGAAGTGCCCCAACACGACCATTTTTTAGAGGTGCCTCGGTCTTTCGTAACTAACTTTGTATA
ATTGTTCAGCTAGAGATAGAGCGCCTTTCGAGTGAGTGTCGTAAAACCAAAACCA
>scaffold_266 342400 360489
CCCAATTGCARCTCTGTTCCTTCACAAGCTCTCTTCTAAGGTTTGTTCTTTCAATTTTTTAATTATTTCCTAAGCTAGTT
GGGTTGGTCATGAACAGTTGCTGTTGTAATGAATCCATTTTCTTTCTTTTTTTTTCGCATTGTAGAGATAAATGATGAAT
TCTTTGTAGCATTTGTTTATGTTATAGATTAGTATAATTTCAACATATTACGACTCAGTACAAAACATTAAATGACACTA

AGGCTTTGAGAAATGAAAATCGCGAGCTTTACATTCCTCAAAGCTTGTATAAAAGTCAATGATCATTGTAAGTTTTAATT
AAATAACTACAGGCGTTTAAAAATTTGGCGTTTTTCTATAATTTGTTCATGAAAGGAAGAAGTCTCGAACACTTTATATG
TGGCTTCCGTAGATAATGCTATTTATAAGTGAAACTCGTTTTCTTGAGAAAACWTTTCCTTAGAAAATAGTAGTTTAAAC
ATCCTTTTTTTATCAGCAAAGGTAAGTGCACTTCGTARGAAAATTAACAGGTTTTAATGTTAATCATTTTATAAATAATT
ATTATGCAATTATTTGCAATATCTAGTAATTTCTTTTCGATCCTCCAATCGTGCGTCAAATTGTGTAATATTTTTAATTT
TAAAACATTTTTGTCCAAAATATTTTTCTCAGAATTGGCAAATCCTTTTGGAATTTGTTGGTTAGTTCAATGCAGTACGC
ACTTAGGCTTTTGTGTGATGGATGTTAGTGTACAGGGTTCTGAACTTCGATACTTTAAGAGTTGCAATACCTTACGTGCT
GGTCATCAGCTATTGGCGTCACTAAGTTCTGACAAGTTCAATACCTCTGATTCAACATTTCAAGAGAGATAAACAATGAA
ATGATCCTTGCTAGAAATCCAGGCTTTTGTTCCTTTTTGAAATAAGTTTTCATAACCTGGTTAAAATTTACTCGAACGGG
AGTTAGTTTTTTGGCTGTCGCATTTAATTTGGGTGCTTTTTGGACGAGGAGCGAAATTTAAGGGGGCACATCCATGACTT
TGCTGTAATATCAGGGCCAGTTATGTAAGAAGAATAAGCAATTACGGTGAGGAAACGAGCTTCAAACTTTTTCATGAAAG
TTAACTGACTGTTTGGTTGTCTCTACAGAAAAGAGTTTGTAATTCTAAAAGCTCTTTTAGTTAAACAGCTACCTAAGGCT
GGCAAAGTCTTAAGTATTGGGGTTTCATTAAGAATCGTGGTCGCAAGATAAATACTGTAAAATAAGTTGAAAATATTGTC
CAGGTCTAAAAAAATTGTTCATACCTTGCAAACTTAGGTGCAGAATTTTGTGCAGCAGTAGTTCAATTCTTTGATCTTGG
ATGCCCAAAAACATCCTAAGAATTTTTCTGATGTACGAGATTATGTAAAAACTTCTATTGAAGATGTTTTTTGGGGATTT
AGAGAAGAATTTCTATAAGAGTTAGCTCTGAATCTTAAGTCGTGTTTTTGTTAGATTGGCTACTTACTCGAACTGGGAAA
AAACTTGCTTTTTTGAGACGTGAAAAAAGGCTTGAACCGACAGCAAATTTTTTTTTGTGAAGGTCAACAGTCATACAGAT
TGTGCAATTTAGCCTTAATGTTTATAACCCTCGTGCAAGAAGAAACATTATTTATATTTTACTTCAGTTGTAAAGATCGA
GAGAGTCTGCTCTGATTTTTGCATTAACATCACTCTTAAGATATTGTCAATAGATTTTGGTTTCATTACGAACACACGAA
AATTAAACTTCTTGTGACAATACTCGAAACAACTCAGTACATTTATTAACTTAATTATGGTAACTTCACAARTTAAGTTA
AGACGGTGACGTTTAATTCCTGTTGAAAGCTGCAGACATTTCATGAGAGCTTTTGCACTGTTTTGTTTTGATATTGGARC
CGATATTATGTAGGTCACGGATTGTGGTCCCAATATTTTTGTGGGCAATTTTTTTCCATTTAAAAACTAATCAATTTACG
TCATATTTGCCATGGCAATGCTTTCATTGACTTGTAAACCATATAAAATGTTTAGACATCTAATTTTTTAATCATAGCTA
AGAATACTTCTTTCATGACTTCGGGAGCTCGATAATTTTGCGTAGGTTAAGTGCTGGAGAGAGTTGTCTTGCATGACTAA
AGCAATTTAACCTATATTATTAAATGAGTCATAAATTCTAAAATTTTTGACAGGACCACTTCATATACACCAATAAACAA
CACAAAAGAGTAAAGCGTCTTAATAACAAGAATAAACATGATCTTTATACAATGATGAATTAGTAACACCTTCAGAATGA
TGGCAACTTCTTTAAAGCTATCACACTGCATAGTCCTGTCTTCCTCCTCCCCCTCAGAACTAAACAGAAAAATTAATATA
TAAAATAATTTTGTATAAGAAATCAGATTTAGCAAGTTTTCTAAGGGTCTGACCTCAAGAATAGTTTTGGTAGAATGAAA
CTAGTGAAGGTAAAGTAAGAACTCGTGTAATTGAAAAAAAAGGCCAAAATGAAATTACATTTGACGATAAAAACTTGCGA
CAAAACGTTGCTTGATAACATTGTAAAATTTAAAAACGATAAAAAGCTATAGAAAGCTAAAATCGACGACGTTTCGACGT
TAACATAACGTTTTTATCAAGTCAAGAATGTACAAAAATAAGTTCTATAAATACTAAAAACGAACAATAAGAGAAAATGA
TTTGCGGGTTAAACAAAAAGTTTAGCACGTATGGAGTCCGTTTGCACGTTCAGATTAGGTTTGAATTTCTTAATACAAAG
CATTTCAAACACTAAACAATTAAATTTGCTTTGACACTTTCTCAGAATCTTAAAATGACTATCATTCAAACGATCCCTCC
TACCATGCGCTTCATGAAAATGCTTGCCAATTGCCGAATTTTTGTGTTCAGCGACACGTTGAAAAAGGTGTCGGCCGTA
TAGCCGACATAATCTGCATCGCACAGATCACATAAAAAATGATAAACAACACATCGCTGATTAACAATTGACGGCTTGGG
TTCTTTAGGTTTAAGGTCTTGTTCTAATTTCTTGCTTACAAAAACTGGCTGCAAAACGAGGCCAATCTTATTACTGAGAT
CACACAGCTGTCTCCGAACAGAATTAGCTGAAACCTGATCTTTGATCTCGTCAGCATTTCTCTCCGCTGTGCCTACAGAA
GGTTTACGAGAAACAAAATTGGAAATAACAGAATCTATAAGACTACAAGGGTAATCAAGATGGCTAAAAATGGAGCGCAA
TTTGGCGCATTCTTCATTAAAAGCCTCTATTGTGGAGGATAGCGTATAAGCGCGATGTAACAATGTCTTTAATAAAGAKT
ATTTATTAAAGTCACTTCTTCTACGTAAAATGAAATTAGTGCACAGAAAGTAAACCTGGAAAAAAATCTCTTCGTCCCTC
CAAATGATTTTTTGAGAAGAAATAATTTAAAAGAGATGTCATTTTTACTAAAATTATTTTGCATAAATGATTTGAACAAA
AATTTGAAAGTATTTTGTTAGAAATTTAAACAAAGAATAGGCGTCTTAGAAAGTTCCAGCACCTTCCGTTCCCTCTGTGA
CTTCTTGAACGTTCTTTTGGTGGGCAYCAGTTTCCACATTCACTCTATCTAATCAGCGGCATCTTGTGTTGATAACTGAG
GTAGCCAGAAAAAATGTTATTGTTCGTAAGTGGCAAAGGTCTGCTCTTGTACATGATCCTGTTTTTTTAGTGATATGTTCC
TGAACTTGTCCGTCGGGGTTGTCATGGTAACTGGATTTTGTGCTCTTGACCCAGTGTTTTAGACTTACACCCAATCATAT
GAAACATGAATTATTGCTCCCAAGTAATATTTTTTTACAGAGTTTCTCTTTGATTTGTTTCGTTTTGTTTTTTCGAGAAT
AATGAAAATTAAAGAAATTTTTGCTTTTCAATGATGTTGATAAAAGATTTGCTCTGCTTCCGCTGGTAAAAGTGGCGTTT
TCAAATGCGCCGTATATCGATGGTTATGCAAGGTCAGCTCGAAAATTATACCATGATAATTTTGGGATTACAACCAATTT
CAAGTAATGTGTAACCTGAATGGGAAATCATGATAAGAAAATCAGTGCAAATTGGATTATTCAACAACTATTACCTTCCC
CCTAAATTTCTTCAATTTTTTTTTTTCAAGCTGACCTTTGTTTGTTGATAACAAACTAATTTTTCATGCAAACAATTTTG
GTATTSACAAACAATAAACTCAAGCTCTCACATTTGTAGTTTTGATCAAATGAGAAAAAAAGTTTACAATTGCCTTTGTT
AGAATCTGCTTTTCGACGTGAGTTTTAGCGATGAAAAAGCTTTAATAATATCATCTCGTTTTTTTTCTTGAAAAGAGAAA
AGATGTTTGAAGTGACATCTTTGAGATGGTTATGACCTTTGAATAATCAAAACTCTCAGATTTTTTTTAATAGCCATGGA
AAAATTTTTGCAAGGGTTCCTGCTTTTAGGGTTTTTTTTCCGTAGGAAATTTAAGATATGACTTTTGATAGAACATCCAG
CTAATTTTTTTTTTGAAAATTGTACACAGCAAAATCTTTTGGCTTTGCAGAGTAAAATTATTTTTAAGATCTAAGTGGTA
AAAAGATTATATCATTGACTTAATTTTGATTAATCTTATCTCAGTTTAACCCTTAAACCCCTAACAGTGACTTACATTTA
ATTCCTTCTCACAGCATCACAGCTAAATCAATCATTAAGGTCTTTAAAATGAGGAAAGTGATTGCAAACTAAAGAAGCTC
GTGATTGTCCAACAAATTCTTCTTGTCATTACCAATGGAAATATAAAAGGAACAATGTGGAGAAAAATAATATTGATTTG
AAGGTGTCAAAGGTTAACCTTTGTAGATTGTCGAGGAAATCGTATTTSTAATGCTTCTACGTTAGATACTAAAGTTTAGT
TTTTTCTTAGGTCTTAGAATCTGTTCTTTTATGAAATGTGAATAGTTATCCCTATTCTCTGCATTTTCTGTGGATGTTAT

TGCCTAATTAGATGCTCATAATAAACGTAGTTCCCTTTTGGAATTATCACTGTTCTATCAGAAAGGCAAGGTATTAGAAC
TTACTAAGTTTAAAGGCAGAAAAAGCGTTTGCAATCTTTACTATATGAAGAAGTATACTGTCATTGATGATAACACAAAG
TTGTGTCATCATTTTAACTCATTATCATGAGTACGTAGAAGAATGATTCCATAACTTACAAATTTATCCCTGGTTTAAGC
TGTTAATGTATTTATGCACTCGTCTCCAAGTATTGGCTCCTCAGGCTTTTTAAAGCAAAGAAAGTATTGCTCGACAATGT
TTGGTTTTAGCTTTGTTTAACTTGATAAGGTTATTTAGGCCACAAATGATCATGGCTTGATCAAAGAATTTCTTAAAATG
CCAGGAAAATCGATAACATGAAATTGAACATCACACATGCAAAGAAGGTAAAGCAAATTATTTTGTTGATGTAATTGAAA
ACACCATAAATTTTGAATTGTCAGAATATTGCCTTTATGGTGTTATATGGTTCAGGAAATTTGTAGAACCAAATATATTT
AGTGATTGTGTAATTGTCTCACTTGGACTATGGACTAACCTATTGTTAAAGGTTTATGAAGACTAGCAGACTGTCTGGTG
TTTGTTGATATACATGGTAGTTAAAAAGATTGGATTGCATATTGCATTGTCACGGGTAAGGCGCTGTGATCGCAGGTTTG
GCTTATCAACGATAACCTCAGGAAAAAATCWTTGTCTTTTTTGGCCATTTTTGTTACTACTAACAACAAATATTTACAGAC
AACTTCTCTGCAAGTAGGTACTTATTGTTAAAAATTCCTTTCTTGCACAACAGYAGTACGTCAAAAGCATTGACATCAGC
AGTGAGTTAGCATAATCCTAAACTAGTGATGTGTCGCTAAATGATCGTGCATTTGTGATTATCATATTTAATGAGCTTGC
TGTTCCTTGGAAAAGGATCTAATCTTCTGTAAGGTAAAGGTCACATTTCTTGGAATAATTTTCCTCTTTTACGACTGTGT
TACTGGTACATTTTTAATGTATTGTGAGCTTTGGCAATCGCAATGTTTTGGAAAAGTATTGTTTCTCCGATATGGTATGA
TTACTAGATTTTTCATAGCTTCAGTCCTACTTTTTATGTTTATATTTCATGATATGGAAWACTCAATTTGTGATTACCAA
AGAAAGCAAAAGTTTACCTCCAGGACTCAACTTTCCAAGCTGATACTCCATGACAGTGTTTAGGATGGTCATTCTCTAAA
CAGAACTTTGACACTACTACTCTATTTGAATTTTTAGCTTTTCTCTGTTTTAAAAAAGTGTAAGAACTACGTGTTTCCTG
TTTTTTATTTTTATTTTTTTTTTTTTGCTAAGACCTCTTTGTTATCTTTGCTCTAACTTGCTCTTACTTAACCAACTTGG
TGCTGAAGTAGATCCACATTTTTTCCGATATGAATCCTTTCAACAATGCTTTATTATTCAGTAGAATTACAATTTTTTTG
YATTTAGTTATTCAGTCCTTTGAGGACCGTAGTATTAAGAAACCAACATACAAAAAGACTTCGGGAATGATTTATTCGGT
AATATAGGAAAAATGAATAGAATTCGAAACTGAATTTCCCCAGGGGAAGCTTTGTTAATATTACTTTTGCGTGTGTTTTA
TTTTTGCATCTTTCAAACTCATGATATACTGCCAATATTGTCAGTATATCAGTATTTTTTGTATAACTACCATTAAGTTT
GTTTTAGTCCTTTCAGTTTTTTCGAAATTATGAAATAGTTTTACTTTCCATTTTTCACGTATTGGTGCTGCTTTATCTAA
TATATAACTGATTAAAAGGAACCTCCACTCTTTTAACAAATAGCGCTCAAAATGTTCCAAATGTACTTCTCAATAGATCT
GGTATATAAAAAATTCCCCAACCAATTGCTAGCGATCTGATTTTTTATCGTTTTTTAAAGAAGGTACTAGTGGCCACTGA
ATTTCCCACGGTCGCCATCTTGAAAAGTGCGCAATGCACTCTGGGCTGATGAAGTAAAATTGTTGCTCGGTGAACTCGCG
GTGTAGTTTCTTACTCTTGATGATGCTATATTGCTGTCGTCTTTAGAAAATTTCAGTGAATGTGTCACAAATCTTCCCCR
TGAACTTTTCGAGCGGCGGTCCACATTTTCAGAATTAAAACCTGATGCTTCTCCATATTCTTCATCTTCTACTTCACTTC
CCAGTCTCTCTTCACTTGAAGAGTGCTCCAGCTCCAACTTATGGGAAAGATCTGTGACACATTCACTAAAATTTTCTTCA
GACGATGGCAATACAGCATCAACAAGAGAGAAACAGCACACCGTGAGTACACCGAGCAACCATTACATTTGAACATTTT
GAGCGCTATTTTTTATGAGTGGAGATTCCCTTTAAGAGATGGTAAACAAAAAAAAATTAACTTTGTTGATGGGGGAACTT
CTTGATTTAGAAACAGTTGTTGATAGCTAAATTAAAAAAATATTTTTCTTTGGAAATTACATCCCAGGACAGCATGTAAA
GTAAGCAACTTCCTCATGTGCTTTATTGTACCTCGTACAGGAACACCTATGAAGAACTTGTTCGCATGCTTTGTTTATGT
TAGTTACAATTGCTTTTATTTACACACCTGTATAAACTAGGATGTTCAATGCATCCTGGAAAATGTGCAGTATTTAAGTA
GATTATGAAAGAATAGTGTGAAATGATCGCTGTAGAGTTTCTAAAGCTGACGTATCGAGAGTTAGCCCTTTTTTCAAAGC
ATAGTATTTGTTTTAGCCGTAGATGTCTGTTGYATATTGTTACCTGACTGGAAACAGCGAAAGTTCTTTGGTTGTGCTTA
GAGCTTCATCGTATTATGATAACCTTGACTTAGAGAATATCCCTTCGTTTGTCTTTCTAGATCATACCTGAATTCTCAAA
CTCGTACAAATAAGMATCACAGAACTGGTCAGAGATGGTCAAAGGTGAGGGAGACACTACAGATAACAAGAGAAAAGGTG
CTCTTCCAAAAATCCAGGAGGGAACATCGGAAAGTMACTATGAAGGTGACCATAGTCATGACCATAGCAGGTTCATGCCA
TCTGTGGAGGAATGTCGGTACCTAAACGACCAGGGGCAGCATATAGACTCAGATAAGGTGCCTCATTATCATCATTACCG
AACCATCTCAGAAAGAGAGGAATTTCAGTCCAGGATTCGAAACGGTGCGGTCTCAATGAAGGACATTGAAGTGCTGCAAG
AACGCTTGGAGAGAGACAACGATAACAATATTTTGGCAGGAATGTCGGACTCTATCCAAATGGCAGGTATGTTGTAAAAT
TGCAGATAGATCTMATTTCTCTGACAGGAAAACCTCTTTTATGGTGTTTGTAAAAAAATACATTTGCTAACCATTGTAAA
GGTGCGTTTTATACAACTCTTGCGCATCATGGCTACGCTGAGGAGTTAAGCAAATTTTAAGGTGCTTAGCAGAGCATTAT
GTCGATAATCGCGAGGGCAGGCAATTGCAAATAATGATAGAGCACTTTTCGATGGAGTGTAGTAGAATCAAAACCAAAGT
AATCACAACGACCTATCAGAAGAAAGGAAAACACCTTTAAGAACCAATGAGAACTCAAGGTAAAAACAACCAAACTGCCT
CAAGCGCGGGACCAAGACGTGATTGGTTTCAGTTTTKCATATGATTGGTTAAGAAAGTGTGCGAGTTTCCCAGACCGGTC
ACTTTGAGGGTCTCATTATGCTGCCGAATTTTTAAGTTGAACGCGTGCTTTGAAAAATGCGGAGTTTGACTGATGATGCC
TCCTCGTTGTTGGAGTTTTTTTAAGTCTTGTGTCAACTGAGTTGGCAACCAAACAAACTTTCTTTTAGTACTGAATCTCT
TGCAACTTGATTGAATTAATTATTTGTCGATCTTATTTTCTCCTTGAAGGACTTCGCAAATCTCAACGCAAATTGTCAGG
CATGCAGACTCTTTACAGCTCAACAGACATCAGCGCTAGTTTGGACAAGTTTCCTGTCTTATATGATCGTAATCCTCACG
AGGTAAGACTCAGCAGCARCTTTTAGATCAATCCACCCCTCCCTGGCTAGTTTTAATGACCAAAAAGGGGTGGGGCGATA
AAAATTGGTAACCTTTATCCATCACTCTCCTCGTTTATATAAAGTCATTACATCGCTTTGGTGATACTGCTCAATCACTT
CCAGTCTCGCGAGGATGACAACTCTATCAGTGAGAGAGAGTGAAACACAATGCAGGTCAAACYTCCTTTGGATAGTTATT
GAAAACATGACTTACGTCCGAATATATTGCAAAAGACCACTTTTTGCCAATTCAGTCATATACAAAGAGCCTCCACTCGC
TAGACGTGCAATGTCGGTAAAACCAAACAATCACCACTTCTTGGTAAAGTTTGCGTAGCGCACAAAATTCAGCATTGTTT
GGGGAAGCTGGGGGGGGGGGAAGGTAATATCGGGTCAAGTTTACCAAGATTATTTAGTTCCCCTGTTGTCTAAGACCCCT
AAAATAATCAATACCAAACTGGAGTCATTTTTACTGGATCGTTTCATTGGATGGTGAAGATGATCAGTGATGGCACAGTC
TAGGTAACGTAGTTTGTGTGAAGGGGGCTAATGATTTTAGAGGGTAAMCCTTTACAACCTAACATCWGTATGCATGTTCT
CCATACATTTCCTGAGGTGCTGACATGGAGACTTTGTTTAACAATGTGTATGTGATTCAGGGATAATACTTTAAGGAGAA

GTTAGGCACTGGTCACTATTAGGATTCAAAGGGTTCACTGGTGCTCAGTGCRCTTTTATATGACTTGMATTTGTTAATCA
CATAGTGATAAAGCTTCTTTTGCTTTATAGGCATTTGTTGAGTTGGAAGAACTTAGCAAGCACGGCGAGGAGATGGAATG
GAAGGAGACAGCTCGATGGATTAAGTTTGAAGAGAATGTTGAAGATGGTGACCGCTGGGGAAAGCCACATGTGGCTTCAC
TAACCTTCCACAGTTTGCTAGAACTACGTAAAGGCTTAGAAAAAGGTATGAGAATTAAGGTGTCCGTGGTGAGAGTTAGG
TTTTAAATCAGACAATTTCCTCCAGTTGATAGTTCTATCTTCTCGTCACCTTTCTACTTGAAGGTTTATTGACATTGTAA
GGAAAAATTCCTTTCTGGTCACTCTTGGGAGTGAAGGGTTTAAGTAATGCTTCGTTGCCATTGTAGATGTGGTTTCACAK
GTGTTTAACATGTATTTCGTTCTCTCAGGGACTGTCTTGTTGGACTTGGAGAAGTTTGATTTACCTAACATCGCTAAGGC
TGTGGTAGAGAACATGGTGCTCTCAGACCAGCTCAAACAGAAGGACAGTAACAAGGTCCTTACGGCGCTACTGTTGCGAC
ACAGACATCAACATCAACGACGTCCTTCATCCAAGAGGAGAAAGAAGTTGGCTGTAGCAGCTGATAACTTGGGATTTAAT
AGCCTAGCAGAGACTGGAAACGGTGTCATTCGAATGAATATGAATGAATCAAATGGTGATGTGCCAATAACTCAGGTTTG
TAGTGTTGTGTTAATGTGAAGCCAGTAGTCTTCTCCAAGTGAAATTCTTGTTTTAAAAATTCTTACTACAGATTACCGGG
GCTATGGCATTGTCATTATCATTATCATATTCTTCATTGCAAAACAATAGCTCTATATACCCTCAAGTGGTACACTCACA
CCAAAGTAGAGACCACTAGTCACAACTTCTTACTTTAATGTGATGTGTTTGACTTTCTCTTCATTTTTTATTGACAACGA
ACAGGATAAAGTTGACATTGAAACTGCCGGTTTGGATGAAGATGAGGAAAAGCAGGAGTCAGATGTAAAAGATAAAATTC
CTCAGGGAGCCGAAGCCACGAACGTCCTTGTTGGAACACTGGATGAACTACAGGACCCAGTAATGGCGTTTGTACGGCTT
GCTAAAGGAGTCCATCTAGAGGAAATGTCAGAAGTGTCCATACCCGTTAGGTTCCTGTTTATCATGTTAGGTCCAAGCTC
ACATGGAGAAATTTATCATCAGATTGGGCGCTCTGTGGCCACTCTTATGTCCGACCAGGTAAGGGTCTTCGTTGATTGAC
AAAGCGGTGCTCACAGGGGAACCACAGGGTGCTTGAAGGCTATTGTGTGAGAACTCTGAGTTGTCCAGCTCTGGAAAGCT
TTCGCAAAGAAAAGTGATACAAGTCATCTTAAGACAAAAGAGAACTAACGTTAACAGCTCGTCAAAGCGCATTTCAACTG
AGTGTCGCAAGACCAAAACCAAAGCAGTCACAACGACCAATAARAACTAACGACGCCTGGAGCGCGAGAAAACGCRAGTG
ACCAGGACGCCGTTGGTTTTAGTGTTCATCTGATTGGTTAAAAGGATAGGACGAATATTATCCACCAATCACATCTTGAA
GTGAAAAGAACAAAACCAGCAAGATCCCGGATTACTTTTGATACTCAGTCAGAAATTGCTTTGGTACATAATTGTAAAAT
CCACGATGATAAGAAAAATGAATTTTTTTTCCAGGCTTTTCATGACGTTGCTTACCGCGCTGACTCTCGTGAAGAACTCC
TGCAATCCATCAATGAGTTTCTTGAAGACACTCTAGTTCTGCCACCAGGCAACTGGGATCGAAGCGTTCTTCTTCCCATT
CTTATTGCTCAAAGYAGAGCCATAGCTCTCAGAAGAAAGCTGGCCAAAGCTGGTGAGAATATTGCACTTTGTTTGACAGT
GAACTCCTTATTACGAACGCCTTTATGATKAGCTCGTACGCAGATTTATTAACGTGGAGGGGGGGGGGGCTGTAGCACCT
TGTAAAATCCTTTGCAACTGCAAGGAGAACTTTATTTTTGTTGGCACATGGCAGTAGCGGATCTGGGGAAAGGGGCCCAA
GGGGCCTTGGTCTTCCCCTTGTTTTGGGTAAAAATTTAAATTGCGGAAGCAAGAAAAGCCGGCAGGTGTACACACACATA
ACCCCTCCCCCACCCTTAGCTCAASATCTGGATCCGCCTCTGCGTRATTGTTCCTGCAAACTGGACTAGGGGGAGATGAA
AATCTCGTAGGCGCGTGTAACTGTATATCAATAACGTTTTGTGAGCGGGGAAATAAAGTCAGAAGGGGGAACTTATAGGT
GCGATTTCACTCTGCAAACCTCACCTTGTGACGATTTATCGACAGCTTCATCCTCCCATTCGGAAGGAGACCCTGCTTTG
GCTCGCACAGGAACCTTCTTTGGGGGGCTTATCCAGGACGTCAAGAGACGAGGCAAAGTTTACCTCAGCGATTTCAGAGA
TGGTTTCAATCTTCACACTCTCCTGGCATCTCTTTTCCTATATTTCGCTTTATTTGCGACAAATATCGCTTTTGGAGGAC
TGTTTGAAGACAAAACTGAAGGCTGGCTTGGTTTGACTGAAGTGATTTTTGCAGCTTGCGCTTGTAGTATCCTGTTTGGG
TTGTTTGGAGGGCAGCCCATAATGATCATTGGCGCCACTGGTCCAATGTTGGTGTTCGAACAAAGTATATATGAGGTAAA
TATCAGCCCAGCACATGTGGCTTTCCCCAAAACCTTTAACACTCGGACACGGCGCCTGCAGTTCCTTTTTGCAAAGAAAA
TTAACTAACCATTAGGCTATTGCGTCTTCCAAAGTTCCTACGGTTATGAGAAAAATCGACGATCGATTAGATAGATTTTG
ATCTGACACTAAAATCTCCCGCGTATTTGTCAAAGGATACTGTAGACATCAGCGTAGATAATTTCTCTTTCAAGCTTAAA
ATTAAAGAAGTCAATGTCCTTGTCTTATGATGATGACGATGATGATAATGATGATGATGATAATGATTATGATGATTAAT
ATATTATTATTAACATGTTATTATTTCAGTTTTGTAAGGCATACGATGTGGAGTTCCTTACTTGGCGCTGTTGGATCGGG
TTCTGGGTAATGATCATCTTGTTCGGCGTGGTAGCCTTGGAGGGCTGTTTCTTGATCAAATACTTTACGCGTTTTACAGA
GGACATCTTTGAACTTCTCATCTCAGCAATCTTCATCTACGAACCACTTGCACTGCTGATCAAGGTAAAGGATCCTTTTT
TTGAATGCGTGAACGTGATTCCAAAKAATAAGTGACTGCATCGTTCAGTTATTTAGTCATTCCTAGCCGTAAAGTAAGAG
ACTAGCCATTATTTATCGCCCCTCGAGGGGAGGGTGGGGGTTGGGGAGATCACTTGGTTTTCAGGGGAAACAGAGCGGGG
GAGGGCTAGTCATCGCCGAAAGAGCACGTATAATTAACCGGCAATGAGATGGGATAATTCGAATATTACAGAGCCTTTTG
GGGCGGATCAAGTGATATTATCGTGACACAACCAAAATACCCCCCTCCCCAATCCTCCGACGATAAGTGATAGCCGGCCT
TTAATTAATTCCGATTATTTGCAGCTTTTCAAAAAGAATCCACTGAACAGGGTAGGGAGCGGAAAATACTCAAGCGTGGA
CGAAGTCAAAGGAGAACCCAACACAGCTTTGTTGTCCACCATCTTGGTTCTGGGAACGTTTTTCATCGCTTTCTACATGC
GCAAGCTCAGAACAAGTCACTTCTTTGGAAAGCGGGTTTGTACCACTCTTTTTTTCTTTGACATTCACATTACCTATCTG
AATGTTCATGCCTTTATTTGGCATGAATGTTTACCTGTTTGTTGTGTTCATTTTAAGTGAAGGCAACATTTATTTAAGAG
TGCGCTTACGAGAAACTGATTTTCCAAATGTGAAAGAAGTTGCGCGCGCGGAACAACTTTATACTTATCAGCGGCTACAA
GCGCGGGAAAACAACGCTCTGTGATTGGTGTAGAAAACTCTTGCCACCGTCTCAACCAGTCAGGCACAAAACGAAACTCA
GTCGCGACTTGGTCACTTGCACTTTCCCACGCTCTTCGTTGCATTGAGTTCTGATTGGCTCCATGTTATATTTTCCTTCG
ATCTTATTGGCCATCGTGATTACTTTGGCCATCGTGATTACTCAACCTAAATGYGCATTAATTGAAAAACGTTCGTTTGG
TCTCCTTGCAGGCCCGAAGACTAGTCAGTGACTCTGGTATTGTCATCGCTATGTTCATTATGGCTATGATCGATTTTGCC
CTCGGTGACAAAGTCATCACCCAGCATATATCCATCAATGATCCTGTGACGGAAGGCTTCAAGCCCACGCAACACGATAA
ACGAGGTTGGTTCATCAATCCTGGAGGCATGAACAAAGAAATGTCGATGCCATGGATCTTTGCAGCCATCATTCCTGCAG
TTTTTGTTGGTATCTTGCTGTTCATGGAAACGGAAATGACCGGGGTGCTTTTGAGCAAGAAAGAACACAAGCTTGCCAAA
GGTCCAGGGTATAATATGGATATGTGTGTGGTGGGTATGTTAGCTTTCGGATGTTCCCTGCTCGGGTTGCCATGGATGTG
CGCGGATACAGTGCGATCGGCTTCGCACGTGAATGCGCTGTCCATCTGGAGCACGTCGCATGCTCCTGGAGAGAAGCCGC

ATCTTATTGAAGTCAAGGAGCAGAGAATCTCGAACATCATAGTTCATGTCCTGACAGGTGAGTTTATACAGCTTGACTCT
AGTGGGGATGGGGGGGGGGAGAGTGGAGGGTTGAAACCTGAGTGGTACTGCGGATAGGGAAATATGCTATAAAGCGGAAA
CTGAGCTGGGAGTGTAAGAAAAAAGGCGAAGCTAAGAGGGCCCTAGAGGATATTTTACCCACCTTTATCAGGTGGAAGGC
TCACAAATTTCGACATTAAAACAACTCAGCAGGCTCCTGAATGGAAGTAAAACATAAGTGTAAGAACCCCTCCTTCCCAC
CCCCCCCCCATTTCACGCGAACATCCTACTTGAATTTCCTTTCTGTATTCGTCTTAACTTACTCAGAGATGTTTTCTCAGT
CGAGTTAGCAAAGCGAAGTAAATCAAACCAAAATAATCCCGAATTACTCGCGACGCTCCATTGAGAATTGCTCCAGGAAA
TTTTAAGGTATCGATGCAAAATGCTGAAGGTTACGTTAACGTGCTCCGGGAAATCTCATAAAATCCGGGGGGAAACTTAT
CATAGAGGAACCCCTTACCCAGGAGAAGTAGCGATACTCATATTAGTTTCATGTCACGGAAACTACGACAAGCTCTGGCA
TTGTCTGGGACTTCAGTTTTTACAATTTTTGCCGTTGTTGCAGGTTTGTCCATTCTGCTTGCTCCAGGGTTGAATGTTAT
TCCCATCCCGATTTTCTTCGGCGTCCTCTTGTACCTGGGTATTGTATCCATGTATGGGCTTCAGATGGTTGACAGGTTCA
TCATGATGTTCATGCCACGTAAACATCACCCGGATGTCGGATACGTCAGGAAGGTACGTCATTTCTTTGTACCCTAAGAG
TGACCAGCATCTAATTTCTCCTTACATAGACACTGCTAAATGATTCATTAAGATCGCGAGAATAAATGAAATGATCGCCA
TCTTAAGGATGCTGGTCTTTAAACACGAAGGCCCGCTATAAAAAGGAGAGGTGCTTTAGGTACCTCTGGACCATTTGTCA
ATCAACCGTCGTGTGATGTAGAAGTCTAAGAGTCAAGTCAGCTTGAGCTACAGCTTATCCAAAGCCAGTGAACATCGGGC
GCATTCCTCAAAATTGTTTTATGTTTCATCACAATACAAGAATTTTTGAGATTTGTATCTCCTTTCTCCAATGGAAACT
GTCTTTATTTGTGGCTGTAAATTTGAAATCGAAGTACGTGGTTCGCACKTTACATCATTGCCACCATGCTGATAGAGGTA
AACATCTCATTAGCTCCTTTTGTTCGTCTACCAGGCACCTAAGGGACCTCTCCTTTTATAGCGCGCCTTCGTGTTTAAGA
AATTCTCCTCGTCAGCACTTTAGGAAACGTATGGAGCACAGTATAGAGAATATGCAAACTGATGTTAGGATGCAGAAAGG
TCAAGAGACGACTAAGAGTCTAAAGGTCAAGGGTTTGTTTCCTCTTAGGGAGCTGGGAGATTTTTTTCAGTCCCACGTTC
GTGAGGAGAATAAATTTTATCTTTCCTAAAAAGCAAAAAATACTTKTTGTTGCCAAATCAAAGCTTACTTCCTTGTTAAC
AAACCCTTATCCGCGAGAGACGAGAGATCCTGGGTACATGTCCTCCTTTGCGCCTTTGTTACGTCACAAGGTGGCACTTA
CCACGCCATATGAGGCAATACAATGAACACTGGAAACAACTTTCTTACGTATTTGATTTTTTTTTCGTGTTTTTTTTT
TATAGGTGAGAACCGGCAAGATCCATTGCTACACTGTTATTCAAGTACTAGCCTTGGCTTTCCTGGTCGGAATYAAGTTA
TCTCCTCTGGCACCATCCTTTCCCTTTTTCATTATCTGCTTGATTCCTCTACGCAAGCTGTTAACAAGGTTTTATAAAGA
GGAAGAACTTGAGGAGGTAGGATGGGCTTAAAAAAKTGTTCATTATCATCTCCAAGAGTGTTGTCTAAATTCTTAGGTTT
TTCATAAGCAGTACGTGGTAATCTTYCTATTTTTTTTGGTTGAATCTTGGGTGTGAAAAAAAAGTGTTTAGAATAACAGAA
GATATTCGCCTTCTTCACAAGTCGAAGAACATCCTTTTTTATCGCCCCGAAAAATCCTGTCTTTCTTCTATTTACTCGCG
ACGCTTCCGGCATTGCAGCTCTTGGCAGTATACATGACGCGCACCATATAAGAACCTAGTGGTTGCCAAGCCTGCCCTAG
AGTCTCTGTAGCTTAGAGGTACAGCGTAGGAGCGCGGAATCTGAGGGCCAGTGGTTCGATTTCTCGCGAGGAACTAGATT
TTATCTTTTTCTCATGTGATGAATAACATTTCTCTTGCTGTTTTATTTTGAGTTTTTTTCTCCCTAACACTTTAACTCCC
AACATCTGATCGTTAATTGTTTGCAAAAACAGCTGATTTGTATTTTGCTTTTATTTTGCAGCTTGACAATTCAGAATCAG
AAGAGGAGGACAGCGACTTTGAACTATAATTAGTTACWGCATCTGCCGTATTCTGCGTGACTGGGTGTTGCGTGACTCAC
TTCACGTCTCTATCATTACGTGACACACGAAAATTATTTATTCGRCGTGAAGAAGAACGCGATGAAGATACAGCCTGCTW
TCCAGTCAGACAGAAAGAACCTTTAATCCACGTAACACTGAATAATGATCGTACTTAACGATCTTAATTATCACTGGCGA
TTGCAAAAGTTTATCTTCTGCTTTATTGTATATGRAATATGGGTGGAACAAAAATAAAGTGGTATGCACAGAGGCCCACG
CGAGGCGAGGAGCGCGTATTCCCTCGCGCTTGTCCCAACCTTCATGCACGCCTCGGGTTTGCTTCGCTAGCTCTTCCCTT
ATCTTCACTCCCCTGAAAAACGCAAAACAAAATTATGACTGTTCTTCAACCTTAGAGCTTCAACCTTCAGCTTCAAATAA
TTTGATAAATTGTGACATCTTCATCTCTACTTGTTTACAATTAGCAATCAGTGAATCCTCTACACTCACGTGACATCTTC
AATCACTGCTCGCTTACAGTTGGCTATCAGTGAATTCTCAACACTTACTTGTTACTTTATGAATTAGTAGACAATTTAAA
GCCTTCCGGCTTTTACATTTTTGTAGCCTTTGGTACATCCAGGATTTATTCAGGTATTCAGTTTTACTACAATGTTACCT
TAAAYCAAGATTTTGTRGATAAAACAGCTCTACATGTCAATATTTTTTGTTAAGGTTATATTTTGTAAGACTTAAAGTTA
TTGAACACTGTAAAATAACTCCAAATTATATTTCGTATATTTGCAATATTGGCGTACTACCGGAAAATTGGTGTAAAATT
GTTAATCATGTCATGGAATTTTATTTTTGGGAATGGAATCTTACAACAAGTAGCATCCTTCAGCTGATGAATTTGTATAT
TCTCGTTGC
>scaffold_374 146951 164338
AACAACAACAACAACAACGAAAAAACATAATTATTAACTAAGACATTGTATTGGTTCTCAAAATGCAAGCTATTGTGATC
AGATATTTGAGTCTTGAGAATTAMAAAAAAATTGTTCCAGTMAACACAAGACATRCATTTTTATTAAGGTAAMCTTACTAA
TTACAAACTAATTTTTAACTTAATGTGTGTTTGGACCATAAATATTCTAACATTACAATACTTTTTACATCCATATCTAA
AAGTTTGGAATATGTGCAGTGAATAAAACTTGTTTACAGTAWGGTTAAAAATTCATAAAGGTTTAAAATTCTAAGCGATC
AGATTGGTTTTCATGATCATCTGAAATTGAGGAAAATAAAAAAAATAAATGTTCTATTCCAACTGATTTAAACAATTTAA
AACCAAAATGAAGATATAAACCTGAAGAAAYGAGTCTGTCATTGTCAAGCTTTATTCTTGGTTCAAAGCTCATKTATGAG
GTGTAGTTCATTCATCAATTACTATACACAGCAACAAATAATGGTGAAATTAGAACCAAGAATAAATTTAACAACATCAG
GTGTAACATAGAGATTGGCAACTACTTCCTGAACAACTTCCATAATATACTGTCAAATATCTCTTATCAAAAATTTACAG
CTTTAACCTACAGTGCTCTCTAAATAGCACTACTCAAATATAAGGTAAACATAATCACAAGATCAAACTGATTGATGTTA
TCACTAACTGAAAACCATAAAAAAATCAGTACATTTAGTTACAAAAGCAAATATTTGATTAAGTGTAAATTACTGTCAAGA
AGGATTCAAACTTAAGTGGTAAGACCACCACTGGAAAATCAAATGCAAAAATTGAAAGTACACCCACAAACACAGAAAAT
GTTCTACTACAAAGTTAAAAAATTGGACGGTTCCCCTTTAGTTATCGCATTAAGCTGGAATAAATAACTTCTGAAATTCA
CAAATACAAGATTGTCCCAAACTTGTTTCATTTTAAATTAAGTGCCAAAATAGATTGCTTTATTACAGGATTGAGTCTGT
TGAGTTTTGGTCATTAAATCCTGGCTGAGTAACTGAAAGAAAACCTTGAAATGAAGTGAAACTAATACCATCCCAGGGAA
GTGAAAAAATATGAGCCCACCTGTGAGGTCTGAGTTTGCATAGTCACTAGGCTTGCGAGAGAGYGCAAACCATGATTAAA

AGGCCAAATTAACAAAAGCTAGAAACAATTCTTAAGAGGACCCGCCTCTGCTATGCCTTAATTACTAAAAATACAAAAAT
ACTGAAATTTATTAGCACAAACTGCTACATGATATTGTTTGTCACCTTTGCTGGAGAAGCTTGTTTGTTAGTGTGTTTGT
TTATTTGTTTTATGGACATAATTTTATCTTACCACCWAGATGGCTTATTCATTAAATATAACATACAATCCAATGTTACT
GTTCACCATTTAAACTTCAAAGCTAACCTGTTACAGTTTTAAACAACAACAAAATGAAATTMTCTACACAAGCCTTTTTC
TAAACTGCTTTATCTACCTTAAGTGTAGTTTTCAAGAASATGAATTCTCAATCAATCTTAACAGAGCTGTTTTCCAACTG
AATGCTTTTTTCCAACTGAATTTTTTTTATACCATCTTGTGACCCACACATCAGTGAAGTTCAATACAGACATTTCCTA
TCAGACATGATCATGTGAACAAAACTGCAAATTATCAAATCTTTTTCAACAGATTATTAATTAAATTTCCAAGATAATTC
TAATTCAATTACAAMTCTTACAATAGATTTTATGCAGTTTTTTTTTAACCGCAAGTTACTTGCCCCATCAAAAGACCTC
GTTTCTATACAGCTTTTTCACCACTATGTTTTAATCTACCCTTTAGTTACCGATCAGAGTTTTTTCATCAATTAATGCAG
TCAATACATATCATCATGCCTGAAGAGAAATCTGAGTTCTAAAATTTAAAAAAATTGAAAGTCCACAGTTATCATATCAT
GTATGTGTCTGCACTTTGACTTGTGTGTGTTACTGGAGATATTAATTAAATAAATTGGGTTGCTGTGCTGTCTGTAGCAA
CCTCAGAGTGTGCCATTTTATGCAATTACAACTGCCTCTTAATCATGTATGCATACTAATATGTTCTCATATGATCAACA
ATACCGGTATAAAATGAATGCCAGAATTCACGCAGGAAACATTCTAAACAGGATTTTCTGAATCACGCGGAGTTTGCTTG
TCAGCTCCTTCATCAGTCACAGTGTTGCCAATTTCATTCAACTCAAGCAAAGACTTCTGTATCTCCTCCATGTCAATAAC
AATATCCTGCTTGTCCTCATTTATTGTAGAGAGCTTTCTTCCCATTTCAACATCAGGTCCTGCATCTTCACCATCTTCCT
TCTTCTTCTCGAGCATCCACAGTCCCTCTTCATCGGCTGATCCTTCACGATCTCTCTTGTGGCGATGGTGCCTGTTTTTG
TGATGGTGTTTGTGTTTATGCTTGGATTTATCACTAGGTTTGTTCTTTCTATTTTTTGCTGTTGTTCCATCCTTGACAAA
CTGTTTCCACAGTGAGCTCCTGCACATCTCCTCTGAGATATTCATTGGTGCTACAACATTTGCAGTGCCATTTAACTCCT
TTCCATCACTGAGGGCCTGGACAGAAAGGTTCAAAAGTAGAATTTACTGGACAGAAAAAACATCTAAGTATCATAACTGA
ACTTTTAGATCATTTAGTCCCKATGTTATAGGTCTTGGTTCATTTTTCATTTTTCATCTCTTAATCTCTTAGTTTTAATC
ATGTAGGAAAAAGAATTCTACTTCTGGAAAGATTAAAAAATAAAAGGTTCTCTTTTCCCATTGAGCCCAAAACCAATTTT
TTGCTTTTCCTTGCATACAGATTAGATACCTGTAAGTCAAAATGGAATTCAACTGTCATACACAGACTACAGGTAARCAA
ATCCAGTCAAAATGGTCTTGGATAATTACTAGGTGCACCAGTCACACCACTAAAAAGTGTTTTTTCTTTTTTTTTTTTAA
TTTTTTTTTTTAAATAGGACTAGCATTATAAGGCATTTGAGACTCTTCACACATGTACAATAAACACTATCTGCTATAC
TAAAAGAAATTTGCATGAGCARAAACAGCACTGATACACAACAGCACAGGTGTCTGCCTCACAGGGGTGGGAAAGGATTW
AACATGAAACACATAAAGCGAAAAAAGTAGGCTTAGCTTTTACTAGTGACACTGAGGCTGATCACAAACCATCTGGTACC
AAACAATAATTGGAGCAAAAAACAATTCAAGAATTACCAAACTGAAAGGTAAAACATGCTAATTAGATAATATGAAAATC
TTTAGACAAAGTAAAGAAAGCTAATGATTATATTCACCTTTTTACCATCAGTTTCATCGTAATGATTACAACCATCCTCA
TCATCTAAATCATCATCGGAATCATAGTCATCATCCTTCTTTTTTGCTGCCTCATCAGCTTGCTGCTTTTTGATGCTCTC
TGGCATCAAGTCATCCAACACTTCAAGTTCATGGAGGTTGAAAATCTTCTCCATTACTTTACGCACAGCAACCAACATCA
ATACCTGTTGACCAAGAGAATGGTTTTGATGAGAAAGCACTGATAGCAGCCAAACATGTTAAAAAAAAAAATGGACGCTT
AAAATGGGTGCTCCTACAGGATACAATACAAACAAAACCAGTTGTGAATGTATATCTATCAAATGATACACCACTTTGAA
TGGTTCCTACTTAATATGATATATCAGAGGACAGACACATCGATGATCATCACCATAAACAGCGTCTGTTTTTTTTTCTA
TCATGTAAAACTAATAGATTGCATGCTGCTGTAGGTCTGTTACTAATAGATCAAAGAGAACATCTAAATGTGGTGACAAC
ACCAGAGTCACACTCAGATGCACCTTGTATAAATAAATAAAATAAATAAATAAACAATTCAGCAACAGACATTCCACTGG
GTGGCTCTTCTTGTCCACTGTTTCCAGATCGGATTGGAATTTGGAATGTTGGTTTTTGCGGAGGGAGGAAAACTGGAGGA
CCCGGAGAAAAACCCTCAGAGCAAGGACAAGAACCAACAACAAACTCAACCCAGGCCACTGCAGCGGTGGGAGGTGAGTG
CTCTCACCACTGTGCCATCCCTGTGCCATCCCTGCTCCCCARTATAACACTTTCTTGTTGTAACCACATTTTGAAGTCAT
CAATGATTTATAACTGAACAGATGTACAGCAATATTGAATCTCTTTGTTCACTAGTTTATCATCTGAATCAATTACAAAT
AGCTCAACCAGTAATTTATTTTGACTTTGTGGTGGGTTTTATGCTGACTCAGAACTTTGTAGTTCTATTTAACCCTTAGA
CCAATGCACATCACTGTACTAAATTAATAAAATGGTTTAACCTTTCATTTTGTAAGCACAAAAAGTAAATACAAACCATA
ACAGGGAAGATCAGGGCAGCATCCGTGGATTTGATGAGCCACAGTATACCCAAACAAAGTAGCTGTATCAGTGTGAAGAT
ATGGATTCTCTTAAGAGGAACTTGTCGCAGAAACATCAAATCAGGTTGATGCTTCACAGGCATAAACATGATCTTTAACC
TTTCAAAGAACTGTTGGCCAAAAGAAAAATACCACAAGTAAATACCCACATTACTTGGTGACCAYAGCATACAAATATCT
CAAGAATAATTATGGCTTTGACCTCTTGTAWGGGGATACTAGTTAATATCTTACAGCAATATTTCTAAATTTGTTAAGTA
ATTCACATTTCCCCCTCGCATTTTAATGAGATATTTGGTTCACTGAAGGACTTCCTGTACATGCAGACTATTTTTAAGAG
GGTGAGAATTAATCACAGAGGCTCACTCTATACCTATTGCATTTTTAACAAGGTTGACTAGAACTAGCAATTTTGACGGT
CCATCAATAAAACCCAATACTGCCTTTCTCACAGGATAAGGGTGCCACAACAGATTTTAAATGTGCCCACTTCTGTCGCT
TCATATTAATTTTGTTCTCAGTAGAGCTGCAAGCACAGTAGTCCTAACACCATCGTTAATTTTTCCCCAAGAATTCACTA
AGTATCCCAGAATCTACATCATCCATGCTTTGAGGTTCTGTTAACAATAACCAGCCATTCATTTATGCCTATCCATGTAC
ATTCACACTATAGGTACAGATTGGCACTAACCTGCAGTCCCCTAAGTGCTGAGAATCCCATGTAGAAGAAGAGACCAAAA
AGAACAGGCATTGGAACAAACTATAGAAAAAAGGTGGCAAATTAAACACAAGGAAATCAAAACCAGTATGACTTAACTCA
TGGAAAAGATTAAGAGCAGACTATGATTGAATGTAGTACCACCAAAACCAAAGTCATCATGACAGCCAATCAGAACAAAT
GAAACTATAAGAGYTAAAAGTAAGAACAAATAAATTTTCTGATGGAAAACAGTAGCAAACATGTCACTATGGGTTTTAGT
TTTGAGTCTGATTGGTTCAGTAATACAAGATTTATCAACCAATTTAAAGTGATGTAAAGCAAAACCAAAACAATCTTAGA
TCCATTTTGACACTGATTTGAAAATCACATCTAAAAYGCATTTTTCTACGGAATGCTTGCCAAATGCAAGAAACACAGAT
AGACTTAAATATTTAAGGAGAGGTAATTAGAAACACAAACCTTAAGTATGGGAGCAATAAACACAGTCAGGCCAATCAAG
ATGAAAATGAATGTTCCAGTGATACGTTGCTCCCTGTGAGGTAATTAATAAATTTAGAGCTGAAGTTATTTTAATATCTG
TTAATCGAGTAATCCATGTTTTTATCTAATACCACAGTGACTGTTTTTTGATGGGCTTGATACAAACATTAAATAACTCA
ATTTATCTACTGTAGGTCAAYGCTACATGTTACGTGTTCTCTTTAAATTTTGCAAAATTTAGCAGTAAGTGTATATCCAA

TGCATGTTAAAGCTGCAAAGCCACAAATCAAACTTACACAAAAATTTCTCGCAACAGAGTAGATCTACATTCTGTTGTTC
ATCAAATTTCTAACAACACAAGTTAAGAACCTTGCAAGTCAATTTACCCCTTTCAAGTAACTTAATTTAATCTGAGGTAT
GTTCTAGAGTATAATGTAATTGTTCAATGACAAGCCAACATACCTCACACCAAGAAATTGTGCTTTCTCTCCAGGAGCTG
TGCACTGAGATTCTACAAACAAACTCTGCACATGACCGACTGACAGAACAGTGGCTGCTACAACCCAGGGCAAACCCAGC
AATGAACATATACCAATGATAAGAGCCACAAGTAGAAGATCAAGATGATAACCTGCTCCTTTCTGCAAAGAGAAAAACAT
AATGTGAGTCTAATTACCAGTTTAATTACAAGCACATACTTATTGCTGATAACTTCTTGGACCAAAAATTAATACTAACC
AACAGTTTGCAACACAACCCTGGTTACTGTAAGTACTTTCAGTTCAAAAGGTTAAGATCAGTGAAATGCAAGTTTTGGGC
TGCAAGGTTATAAGGTTAATCTGGTTTCCCTAAGGCTTATTTTGGCCAAGGGCTAGGATTACTKCAGAGGAGCATTGGTC
TCAGACTAACATTAGCATTTATGCTTATCATCAAGAATTCAGTATAGATCTATAACCTATTTTTACTGTCATTATTGAAA
CTATAATTTCATGTTTGTTTTAAGCTCACTTGATAGAATATCAACTCTGRCTTGYCTAGCAGACAAAATGCAMAGCTTAA
ACATCCCCACTCTTTCTAGCAACACATGGGAATTTCACCATTGTCTGTACCTGAGGTGGGAGGGGGAGGGTTAAACCTTA
TCTTGCTGGGGTGGTCTCAAGCCTTTCCAGCAGAATACAAAAGTTATATCTGTAATACGGAGGTGTTTAAAGGTAAAGAG
TTCATGTTAATGAGCCAGTGGCTCAGAAGAAAAGTTCTAGATAAGGTCTAGTTATGAAARCTAAGTCGATGTTTAGAAAG
TCCTCCCTTTTACAAAAAATCAACYGTCAAATGCAGCCCTGGGGGACAGATGTTAGAGCTTTGTAATGTTCAGTGTCATT
AATATTTTAACAGACACTTCTCAGTTATTAATCACAGTTTTTTAAGCAGGCACTAAAAAGATCTCTACACTATACAGCAC
ATCATGCCATGCACACAAAACAGATCTTCATACCTTGAGCTTGTGTTCTCGTCTGTTTACAATCAGTGCTGTGATTTGCT
GATCCATGAAAACTAAGATAGTGGCTAGTAAAGCTGGGATTGCTGCAGCAGGGATGGTCCAGACAGGGTTTTTACCCAGA
GGAGGGATTATCCAACCTCTTTCTTTACTGTTTGTTGGCTCAAATTTCAATGGGATGTCAAGCTTTGGTGTGTTAACGCC
AAATGCCAAGTCTACACCAACCATTACCATAATACTAATCAGGATAGCAAAATCAGAGATTACAGAGCGGACCTGTTATG
GGAAAGAAATTGAGCAGGTTATTAGAATTATTACATTATTACAGTATAGTTATTACATTCTGGGCAAGAAGTATAGAATA
AAATATTTTCTGGAGCAGTTTTATGGTGAAAGAATAAGAAACAGTTGGAAACAGCATTAAAAAACCCATTTCAATTTAGT
ATTCTATGCATGAAGAAATTTAGGCATTAGGACTGGTCTTCTTCAGTGAAAAGCCTTAGTTTTGATCAAATCTCTGTCTA
CAGCTGTAAGTTGTTAGTATTTCAAGTAATATTTTACCGCAATAATTTCTAGAAACTACGGAGCTTTAGCCAGTGCTTTC
AATAAGATTTGAAATAGGTGGCTACCTCGGTACAACATCCACCGGAGAGCGACTAGATTGCGACTTAGCGTGGCGACTGC
GACTTGAGCGCGGTGAAATATACCCGGCGACTCGCAACAGAATAGCCGCCGGAGCTCCGGCAGGTGCCAGGTGTTATTGA
AAGCACTGTTTAGCACAAGACACGTCAAAAAGAAAAGAACAGGAGACATAGGCATGCTCATTACTGATACTACACTGACT
GTTACAGCTAACATGCGTGTAATTTTTTGCCTCTTTTTGCTTTATTGGTGTGGTGGTTAAAGCACATGTTCAGAAAATGA
AATGACTCACCTTTGTTGGGAAATAGCTGCTGCTACGGAATTCCTTCAGTGTAACTGCCAGGAAAAAGGTTCCGAGAGAT
AGGATGACCGACAAGAAAAACACATTAGAGTGACATGCCTCACCCACAAGCTGACCACCATACCTTACACAATCTCCGAT
TGGATAGTCCAGTGGTGTTATGTTGAAAAAAGATGAGCTGTCTGCCACGTGGTGACCGGAAGAATCTTCTTCTCCCGGAT
AGCATAAACAACTGTGACTAGTTATGTAACCTTTACTCACTGGGCTATCATCCAATACATGGAACAACTTCTTGAAACCC
TCAACAATGAAGATCAATGCAATTAGGGTGGCAAATGACTCTTCTGTGAAACGGGTGAAGTAACGCACAAGGGCACTGGC
ATCTGTTACCACAAGGACAAAACAGTACAGCATCGTCCATATACCAATCCATAAACGGAATGGCATGTAGTCAAGGCCAA
ATTTATCACAGAATGTATACAGAATTTCTTCAAACACTAACATAGGCCCAGTTGCACCCAGAATAATAAGTGGTTGACCA
CCAAATAAGGAGAAAAGGACTCCACCAATTCCACCAGCAAGAATTTGCTCCATAGCAGCCTAAAATTTAGGAAGAGGAGA
GAGAGTTCTGGATTAGTTGCCTGAATAATTCCTGACTGCAAAAAATTGGAAATACGCCTSTGGAGCAGAACTTTGAAGAG
ATGTGATATGTACAAGTGGCTACAAGAAAAATTTGAAAAACAGGAGGAAATATAAAACTTATACAGGAAACAATGCAATG
CCCTGCCCTCTGAAGCAAACCTGTCTGTCTCCATCTGGACAATAATTATATACATAGCATTTTAAGAAAGGTAACCTTTC
TTAATTTAATAGTAATAATAAGCTTACTTTGTTTTAACAACAGCAATCAGCTACTACACTTTTTTCAGAAGATTGATGGA
AACGAGCTAAATTGACCAGGTTTTATGCCAAAACATTAAAAGTAACAACTAAAAACGAGCATATAGCAGCAAGAAAGGAG
AATTGTCAGTCATATCTTACCATGTTCTTCCCTGTCTTTGTCCCCATCAAACCCCCAAATGTAATAATTGGTGTGAGACA
AGCAAAGTAAATGAAGATAATTGATGCAATACACTGAGGGTTCAGAGCATCTTTAAAATCACTCCAGTACCAGGGAGCAC
GACGTCGGATGTCCGCAATGAGACCACCACATAACCTGTCAGAGAGTAAAAATAGATGACTCCCAGTTATCACACAAAAA
TACTGCGAAAACATTGGAGATTTTGGTTTATTTAACAGTAAAGTTTTWAGTCTATATTTTGATCTTAGAATTAACTCAGT
TGACCTTTACTTGATTGTAGATCCTTCTGTTTGCGGTTTAATTACCAAACCATCAAGCAAAACATAGTGCATCAAATTCT
ATATAGGTTTCTTTTCATGCCATCCATCATCTCCTTACCTTCCTGTTTTCTGAAGTTGTTCTTCTCTGTCTCCTGCRGGA
TCATGTTCCTCCATTTCTCCACCACCTCCAGACTTCACAACCTCAAGACGATTTCCCTGCGCTGGAACAGCATCTGGAGG
CTCAATGCGAATGGAGGGATCCCATTCACCAGGTGGCAACACTGTTACCTGAATTCAAATGACACAGAAGTACTCTCTTG
GATACAAAATCAAAGGATTAAAGTTATCACTGTGAACAAGACTATAATGGACTATAAAAGCTATGATTTTTTTTTATGCT
AGCTGTGTGGCAAGCTTGAAATGTTATATATGGCCATAAGTTGTTACAAAGCATATTAATTCACAGGAACAGAGACACCC
TGAAATTCTTCAAATCAAGATGTTTGCTACTGGGCAAGTAATGTTAGTGTGTAAGGAAGTAGAAATATTCATGTAACAAA
TATGTTGTTTGCCCTATTTTATTCATATAATCAGAATGAAGTCAGAGAAGATCACCACATTTATACATCAATTCAGATTC
ATTCCTTCTTTGGCATTTTTAAGAAACAAGGGAACAAATCCTCTAAGAAGCATGCCTTTTCCACCTCACCATCCCATCAG
TACATTGCCAGTCTAGTCAGGTAGAAATATCAACCAATGCCAGAAGAGATATTGATAGAAAAGAGCACAATGATTAAAAG
AATGGAAAAGTTCATGGCTAACTGTTCGGAAATTGGATGATGAAGATATTGTTATTAAATTACCTGCATTAAAAATTCAT
CAATTCCAGCCAGCAAATCCTCACGACATTGTGCTTTGTAGGCAACCTCATGAAAAACCTAAAAAATAGACAAAGAAATT
TAAATTTAACAAAATGCTTACTTCCAGATGGCTTTAATTGCTCTTTTCTTGTTTAAAGCATACCTCATTTAAAGAATGGT
TTGGTATCAAGAAAAGAGAAAAATATGATCCAAGCTAAATCAGATCTAATCACACCTGAATTCATTTCTAGCTAAACCAT
GTACACACACCCCAATCAAATTAGCCAAAGAAAAAAATACAGAGCCATGGTTTTCTCTTTCAATTCACCATTGTCTGAAA
AATGTTTAGTGTTCTGGTCAGATTTAGTATCATAAGCAAAAATTTTGGACACACATCTCACACTAATAACTGGCTAAGAA

AGAAAAGTATCTGAAGTTAGCAACATTTGTAGCAAGAATTTCACACTATTTTGGCATTTTCTGAACCCTTTCACCCTAGG
GGTGACTAACATCTAATTTCTCACTAGGGTGCCCCTTCTGAATCAAACATTAGGCCCTTGAGAATTAAGGAAAATGATCA
TAAACTTAAAAAGCTCCCAATTCTTAACTAAATTCTCCTTGGGTAAAGAACAGTATGGAGAAGAGATAATCTGGTGCTAG
GCTGTAAAGTTTATTAATCTAGCTTTACAAGTAGATCAGAATGTGAACAACGATACCTCATCAGACATCAGTGTCCCAAT
AGCACGTCCAACTTCCATGTACCTTCCTGGTGTACCAGATGGTCCTAACATGAGAAATATAAACTTTGATGGCACAGGAA
CTTCAGTGAGGTCACCTAAGAGTGATGCATTCTCCAAACGTACAAATGCTGTCACTGGCTTCTTGATGATATCCATCTCA
CCAACCAAGACATTGGAAGCTTTAGCGCCCTGAGGAATCTTTTTCATAAAGTTGGCATCAAACTGAAACAGAAATTTTCT
RATTACCTCGATGATAACCCAAAATTTTTAAGTGTACCACCTGTTGATTGACACAACAAGGATATCTTAGGCAAAAAATA
TCAATTTTTGCATAATCCCGCAGCGCTCAGATAATCTTTGATTACTATTCATGTAGAACCAGAGAAGAAGAATGTTTTTT
TAACCTTTTAACTCCCATGAGTGACCAAGACAGAATTTCTCCCTACAATATCAATACAATATCAAGCAGAGAAAAGACAA
AAATGGCAATTCAGGGATTATTGGTTGATCCAATACCAAATTGTCCAAACTAACATCATAAGAATGGCAGATGGTATGGA
GAATTACAAATGAGATCTTGGGAGCGAAGGGTTGGTGCTTCAACTGATTTTTGGTTCCAAAAGTCTAAGCAGACAGTACA
GTGGTTGTCCCTAACCTTTTTCAGAATTGCTACCTGGTTATGTTTAAAATGTAGCTTTCCAGAGTTTTAAATGTGTAGAA
CAGCTCAGAAAAAGTAATTTGGTAATATCAACTAAGTTGAAATTATAAATTGGCCACCATAAAGAGTTAAAAAGCTGACA
TTTTGAGCGTTAGCCCTTCATCAGAGTGAATTGGTGCTTCAACCGATTTTTGGTTCCAAAAGTCTATGCAGACAGTCAAG
TGGTTGTCCCTAAACCCTTTTCAAAATTGCTACCTGGTTATGTTTAGACCATTYGCTCTGACAAAGGGCTAACGCTTGAA
ACATCAGCTTTTTAACTCTTTACAGTGGCCAATTTACAATTTCAACTTAGTTGATATCACCAAATTACCTTGTTACCCCC
ACTGACGCAGCACCACAGTTTCTTTAGAACTTACCCCCTTTATTCATTTAGCTCAGAAACAGGTTACACTACCCAGCTCT
AAGCAAGTAATGGAATGTGCCAGGAATGAAGAAATGCATTACCAAACAAACTTGCTATCAGAGTAAAGAAAATGTGGATT
TCCTTGGTTTTTAATTGAGTGCAACAAATAAGAAAAGGGGTAAACTGACCGCTTCTAAGCAAGTCTCAGAGTCTGTCTGG
TCAAGACCTGCGTTACCATTAACACCACTCTCTTTCCCTTTTCCCACCAAGTCTGCAGCTGATGGYAAATGTGGGAACTC
TCCATCATTTGTGTCTACCTCGTTCACCACCTCTAAATTCTTTGGAGCAGTTGTCTTCTGGGAAGAATCAGATCTTTTGA
ACATGTCAGTGAAGCCAGCGGTCGAGCTTTTCTTTGCAAATGATCCAAAGCTACTCATTGGGTTGGAGCTCTTCTTCTTT
TGTGTTTTGTTTCCCTGGTGACGCTTCTTCTTTAAGATTGCATCACGTACTTGCTGCCTGTTGTCCTGATCCAATTGATT
TTTATTCACCAGATCATCAAGGACAAGATCTTAAAGGAGAAAGATTATCTAATTTCAAAACAGTGTTTACTGTAAYAAAA
TTAATTTAACAAGGTACAGTATTAACCGTTTAACCTCAAGAGTGATAAGCAGCTAACTTCTCCCTGCCAAGTCCTACATT
TAGGTCACAAGAATAACGGACATGATCGCCAGAAATTTTTCTTTAGTTGGCAATCACGTCCGTTATTCTCGTGACCTAAA
TGTATGACTTAGCAGGGAGARGTTAGCTGCTTATCACTCTTAGACATTAAACGGTTAATACTGTACCTTGTTAAATTATT
TTTACTACAGTAAACACTGAAATATGTAGAGAAAACTGTATGGAGAATAGGAATACTACTGGTGTCACTTGCTGGCCAGT
GCTACAAGGTGAAAATGAATACAGTACAACTGCAGCATCATTAGCAAGGCAGCAGTTGCTAGAACAGTACAGTGGAAAAC
TGAGGTTATAATTCAGAAATGCTAATGCAGTTACCAAGCATAGGAAATCCCTTTCAGGTTAAAACTGGCTTAGTTTGTTA
TCAAGATGATGCAAAGTCAAACAGTAAGACTRACACTAGGTGGTATTTCTTTTTTTTGAGGGGGGGGGGTTATCTGGCAA
GTGGAAATGTATGTGGGCAAAGACAATTCAAACAACACTTATTTTGGTTAGCCCAGAAGAGAGAGAGAGACATTCATTAC
AATTATAAAAACGACAGTAAATGATTTGCTTATTAAAAGCTTTGAAGAGATGCTTACCAGCAATTTGAGATAAATTGGTT
GCATTCATGTCCAAAAGTACTGTTCCACGTAAAAGTGCACTTCTCAACTCAAACAGACTGTAAAGTGAAAGGGTACCAAC
ATGCGGCTTACTCCAACGATTTCCACCTTCTTCTACATCCTCCTCAAATTTTAACCACCGAGCCATTTCCCTCCACTCTT
CATTACCATCTTTATCACAATGATATTCCAGCAATTCTGAGAACATTTTGTGTTGCTCTTGATCAGCTTCTTCTCCACCA
AGAATGAAGCGCACTTTGTCTGTTGGCAATGATGGTGTCCCTGCATCTAAAAACAASCACAATTTGTTCAAACTGCACTG
CATAAGAGTAGAGATTGATGGATGAAATAATTCAAATACTGTTTAGTTGTAATTATTTGAGAATATTTACAAAAAAAGCT
TAGTTGGGGGAGAACTCACAACTACATTCTGTCAGGCTTTCATGGATTTCACCTKTTTAAATCATTTTGAGTGACCCTG
TGAGATCACTAGGGTAAAATAACTCTTTAACTCCGATGAGAGATCAAGACAGAATTTCTCCTTACAATAACAATACAGAA
TGGAGCAGACATTGGCAATCATTATGAGAATAATAATAAATATCAATCAGGGGATTATTATTGGATTTAACACCAAGTTC
TCTAAACTAACATCATTTAAGAATTGTATGGCAGAAAGTAGAAAGAATCCCTCAGGAAATCTTGTGCGTGAAAGGATTCA
CATGATAGGRAAGTTTCTGAGTATCAGTGACCCATGTTCTTGTCTGTTCTAGATGTTTAGAAATCAACACCAGATTCTAG
TCAAAACAACACCCCTCTACCTAAAGCTTCCATCTCAACAAACTGAAATTGGTGCTTTGAGAATAACTGCACAATTGAGG
TTTAGTAAATCATAGTAGTAACAGGATTTTCATAACAACAAGCAGCTTTCCATTTTCCTAACCTATATACTTGTAAGTAC
AAGCCAATAATTATTGTGTCAAGTTTGATTTTCAGCTACAACAATTATAATTAATGCATCCATGATCTGCTTATTAAATG
AAAGCATCTTCAATACCCTGTCCAGTCAGCTGCTGCTGCAAATAATTATAGATTTCCCCACTTTTGCTTCCTCAACACTT
AAGAAACATTATTTCAACTCAACACCTATTAATTTTCTTTTTGGAGCTTTTAGGGTTTTCATTGGGAAACATGGTAGCAG
GACAGTTGTGTGATTTGTACTGGAAAAATCTTCTCCAGTTCTGAATATCAAATGGCTTAAGCCAATTAACATTTAGCTGA
GGAAATCTGCATAGCTGTCAGAGAATTATCTGGTCTTTTTATACCTGATGGCTCTTTTCTCAGCTACAGATGCACATATA
AAATCTGAACACAATTATATTATGAAAATTCCCTGTGACAAGTAAGTCACAGTGGGCACATATACTTAATATGTATCCAT
TAAACATACATACCCATCTGTGCAGTACCATAAGAAAGTGAAAATTAGGCACAGCCTAAGACAATAATGTTACCAGTTTG
TTTTTGGCTGAAATATCTGGTCTGAAACCATTTTTTTCTGTTTATTACGTTATTGTTGTGGTATGTGCCATGTTCTTCCT
TGTTTTAAGTACAACATGTAAGAGAGATTTTCAAACCAGTAGAGCATCCTTTTGGCAGTTGATTTCTCAAGAATTCCAAG
TGATTTTAGGCAGTAATAGGAGGAATACTGATGGAACATTTTTACAGATTACCACCAGGATGTGAAGAATGGAGCATGCT
CATATGCTAGGACTAATGCGTGTGTCAAAACATGCAATTGTGTTTTTTTTTCCTTTGAATATCAATAACTTAGATTAAGA
TCAAGAATTTAAACTTACCAAGAGATTATCTCAAGACGGTTCAATTAGAAAGAATAAGAGCTTAAGCACTATAAATAACC
TGTTTAGGGTTGCAGTCCTGTAAAATTGTAAATTCATTTCAGTGGAAGAATTGCTGATGTTTATTATAGGCTAAACAAAA
TCTCTTTTGTCAACTGAAATACGCATTTAATACACATCAACATCCTACCCCATTTGGAGATTTTAAAACAACACAAGGAA

ACAAAATTTTATGGAAAGTGGGTGTTTGCACAAATTCCAGCCAAATATTGCTGACAGAATGAGGCTAAAAATGTACTGAC
AGAAATAGAAGCAGACCAAAATTTTTAATGTCACATTGAAAATTGTGGGTCTTTGAGATGCGGTTAGTCGTCAATTGAAA
ACAATGCAGTAATTTATTCCAGTCAATTAACTTATGACCAAAATCATCCCACTGTTAAAAACATCACTGCACATACACAG
CCTGGGCATAACTGGTATTTTAAAATTACAATGAAAAGTTATTACTGCTAATGGAAGTTAACCATTTGAGGGCCAAGTAA
TTTTAACAATGACGGATTATTCAAATGACCTGTCATAAGGTGATGGGGAGGTAAACTAAATTGATTACAGCTAGAAGCTC
TTTGAGTCAAACAAAAATTGTACGGCAATGTTAAAAAAAAAAATAACTTTAGACTCTGTCATGTTTCTCAAATACTAATCA
GTCTAAAGACTTCCTCAATAGGTTATCATCTGGAAATTTTTGTTAAAATTCGCTGAAAAATCAAGGATTTATGAATATTA
CACTTTAACACATACCAGTATTGAACTAAAAAAAAATTGGTTCCATCGGGTTTCAAAAATACACGAGCACATTTCTCAAG
CCTTCTATGCAGTGATATTGGTGAGTGGAGACTTCAATACAGACAACATTAATCATCAGTATTTGGTTTTTAAACTGCTC
TCGAAAATACTACGAAATAGGACGAGTACTTGACCAAGGTGAATCGTATATATACATACAGGTATATATATATATATTGAAG
CACATGCCTCAGTTAATGGAACTTGAGTTGATAGCAAAATCGTCGTTAAAAAAGTACGATATTCCGAGCCAGAAAAAATT
ACAATATGAATTTGCACGCAGGATTCACGGCACGAATACATATTTTGGATTGACGATTTCTGCCAGCTCGCACGAAACAT
AAAACCTTCACAATTCTTTGGCTTATCGATCGTTGCGTAATTTAGATCGATCACTACATGCAAGTTGCCAGAGTAAAAAT
AACAACAGCTTTAAAGCTCGTGACACGAGGATGATAAAAACACTCTGTTATAATAATCAAAACTTTTTGTAACGCTACTA
CAAATTTCTCTGAATAATACTCTTTTCGCACCTTTAACAGAAAACAAGCTTCAAGGAACTCACGCTTGTACGATATAAAC
ACTGCTCGCTTAAAAGGTTTTCGCTTTTCTTTTCAACCCTGTTCGACCCCAAAGCAGTGAGCTATCCCCCGACAAATAAA
AAAACAAAGTCGGGTACATTTTGCATTACGTAATGTAGTAAAAAGAACTCTAAAACAAAAAAATATCTACCTGACGAGAG
TTCATCTTTGGATTCGAGTAAGGACCTGTGTCTGCGTCGGTGATGATGGCGCTGATGGTGGTGGTGTTTCCTTTGTCGCA
GAGGAATATGAACTCCTACATAAACCTGACGATGATCTGTAATGAAAATCAACATAATTTAGATGTGCGATCAACAAGTA
ATAAAAGTACTCTTGCATGTAAAAATTTCTGTGCGCGGCTAGCGTGCGCGTTTCCGGCAAAAAAAATTCGATTATTCGCG
CGTACTTTGATAGTCGAGTTCTCCAAAGTAGCCATTTCTACTGGATATTCCTGCGTCAACGGGTCCGGGAATACTATCGT
AACTCTAGGAGAAAGAACGAAAGTTATAATTTCTGCGTGATCACAAAATGGGCGATCACAAAAAAAATTCTGGGCTATTC
ACCGGTTGCCGAATTTCCATGATTCCA
>Contig_6772708 1199 12925
GTTTTTTTTTTGTCTTAAAATGTCAGTTTTGAAAGAGTTACAAGCTGCCTGGCAGCAAGTCGATGGTCATGCATGTACTT
CCTCGAGTATACAAAGTTCTTTCGGCCTTGCACTTTCCTGAAACACAACTTTTACGGCAGTCTTAGTGGTTTTGATAAAA
AGGTTAAATCTATAAAATATATAAAACAACATGCAAAGACCTTTCAATCAACCTAAAGGTGTTGGAAAAGAGGACGAAAA
TGGTTCTGCCACAGGAAGGAGTGTAGACGGCTTTGATATTGCTAATATCGACGAAAGGAATCTAAACAACAGCCAAGGAG
ACGTGACGCCGGCAATGGAAATGTTGAAGTTCACAGACTGTGACGACGATGAAAGTGAAATAGAAACGGTTGAGATAATT
AATTTGAATTCTTGTGGACAAGGGTTTGACAACGAGACAGTGGATGAATCATATTTAGTTGAAGGTAAGAAGGCTTTTTT
TAAAATTGCCGTTAACGTAATGTTCAGTCGATGAGCCAAAAATGTCTGTCACGACTTGCATGTTTCTCTGCACGAGGTAC
GTACGTGTTAGGGCCGGCGAGGTTACCAAACTTTCCCGACAAATTGAATAAACATCCTCCATCAGTGAGAGCAGGTTGTT
TTTGAGATTGAAAAATTTTGTTCCTAGGGTTATCCAGTTTTTTTTGCTCTTTGTAAAGATTCTGTGACAGGAACGTGATG
GTCATGCATGGATCAAAGTCACATTTATAATTTAGTACCTTCAGCAATGTGATCTCTATCACACACCGAGTTTGCTTGGC
TGGGTCTTGATTAGGATGTTTCTTTGCAAGGGGTGTTAGTGTCGGCGAGGTTAACAAACTCTCCTCGCTGAATATACATT
CTCAGTGATAGCAAGTTTTTTTCAAATTGAATACACTGTCATTAGGTGTTCAGGGCCCAACACTAACGGTAGCCAACTAG
CCACATAAGCCACAGACTAGCAGAAATTTAGTTTTGGCAAGTGGTTTTCGATTTCCATTAGTTATTTTGGCTAGTTAACA
AACTGAATTTATTGTAACATCTAAGTTATCACTAGCCAATTGGCTAGTAAGTTGAAAAGTTAGTATTGAGCCCTGGGGTT
CTTAATTTTTGTTTTGGATATGAGATACATGTGACAATAATGTGATGGTCTTGCCTAGATCATGGTCATATTTATATCAT
ACCTTCAGTAATGTGATCTTTATCAAGCAACAAGCTTGATTTGATTAACTGGCCCTAATCACATCTAAACTACCAAAAGG
GCTTCATAAATCTACTCCAAGGGTCTCGTCAGCAAGTGACCTAACTCCTCTACCTAAAGATGCTACCTTTACAACACTTC
ACTGATGGTCAACCATCCAAAGGTTATTGCAATAATGGCATTGGTAAAAAGCCACGACACTTATTGACAGGAATAGCAAA
TATTTACTTTCACTTGATAAGCTACAAGTACTACTTCACATAGCTTTAGAGAGTGGCAAAGGGTGGGTCCTAATCCTGTT
TCAAGCACAAATTTTGGAAAAATTGAAGTGTCATGCATACTTTAAACAGCATTTCACACATCACATATTTAAGAGGGAAA
ACTTCAAATTATTTATACTGTGATGTGCATTCCTGTTCAGAAGCCTAAATGCAATATATGCCTTTGTGTAAAGGCATTGT
TTTGTTCAGGATTTCATCTACTTGGGTAGCACAGTACACTTTTAAAAACTTCTGACCAAACAGTATATCGAAGAACCATT
ACCTCCTCAAACAGAAAATAAAACTGTGGACATTGATACCCTCATAATTAACAAAGTCAAAATTACAATTGAAATGTTTG
TTTTACTGGTTCGGTAAACTTAAGTAGTAAGGTGTAAACCATTCAGCCTAGTTTCCATAGCTAGATTTTTTTACCCACTC
ATTTAATTCTTGATACAGTTAGACCTGTATTAATTGGTCACTCATTGGAAATAGCAGAGTGACTGCTTAATCCAGTTTGA
TTGCATAATATGCAATACTTAGGGATATTAATAAAAATGATACACAATGCCTCTTAATACTGTAATTGACCATTTAGTGT
ACAGAATTTTGCTCCAAAATACTACTTACTTCAATTTTGGGAGAGACACACAGATTAATAATTACTTCAGGGGGTTATTT
GAGTGGTTTGGCTTCAAGTAACCATTCAATAAAGGTGGAAGATAATACAAACAGACCATTAGAACTTAGCAAAGGGTGAT
TGCTATTGCTTAATGGAGGTGGCCATTTAATAGAGTTGAAAATTACTTTGATGAAGGGGAAACAATTTTAGGACTGCGGC
GACAAACTGCTTATCTGCAGGTGATGGCTTAATACATTGCCGCTTAGTACAGGTGCAACTGTCTTCTGATGTGTATAAGC
ACTTTTTCAGTTGATGTCTACTGATTGGATTCATTTTTAGTTGTTCACTTGCTGAATTTTTCCCTTAGAAAAAAAATTAC
CTGCAACATTTATATTCCCCATACATTTTGTAAAATGCCGAAAAGACAAATTTGTTTAACAATCAAGAGCTTCTTTAGTT
GATCATCCCTTTATTCTTGTGACCTTAATGTTTGATTCAGGGATGATAATTATAAGGAGAAATTAGATGCTGGTCTGTGA
GGGGTCGAAGGGTTTAGAAAAAATAATGTTAACAATGCAGTACTTGATGAGAATGTGTTAATCTTTTATTTATAAATATG
TTAAGATTCTCTGATTTAATGGTCAGCAGTGTCTGAGAGTTGGCCCTGTGCATATTGTTTAAATTTGCTTACTTGGTTAA
AAAAGATATCTATTTCAAGGAATTTGTTTTCTTTTAATTCAGAAGATGGTGTTCCTGGTACTTCTCTAATGCTGTCTAGC

CACCAGGTGAATACTAAATTTAATTTAAGTAGATACTTTTTTCAAGATGAATAATTATTCACAGTTACTGTTGTTAGTCA
ATTATAATTATATATTTTGACTGTTAAACCTTCAGAAGCGATTAACTTACATGTATGTCTTCTCCTTTTGATATCCATAC
ACTATCCAGCAAACTTGTCTGGCAGATTTTTTATTTTGATCTACCGTAACTCAGAATTCACATCACTAATTTACAAGAA
AATGTGTAGCTGCTAGAGGGTAGAATTAATAATCAGATCTTGGAAGTTGGTGGGTTAATTTTGAATGTTGGTGGTCTGTT
ACACAACACCACAGGCATGATTAATACTAGCATTGAATGATTATGCACATTGATGATCACCATAATGGTAAGTTTGGAAA
ATAGTAATTACAACAAGATGTAAATATGTGGAAAAAATAGTTGGAAGTGGTTGAACACTTTTGTTGGTGGGGAAGATAGG
TGGAAACATGTTTTTTCAATGGTCGCCTTTGAACATCTGGTGTATAATGAGCCAATGAGATGCTAAACCTTAACATTGAC
AACCACACAATGCTATTAGGACCAAATTTAAGGTGGACTAGTGTTGGATAGCTAGCCTTGAGATGGCATACACTATAAGT
TTAAACAGTATCTTAAATGTCTTGTATTTCTTTTTAGCTTCTTGTACTATGTAATTTCATTTCTTTCAATTTGCTGCATT
GCAGAAACTCCCTTCAAAAAACTTCTGCGCTGAAATCAGAGCAGCCAAGGATGTTGAAACTTTCCTTGAACGACCAATAT
TACTTCTTGGACTGCAGGAGACAAGTCTGCAGGGAATTATTGATGCAATGTTGAAGAAGCTGATAACTGTAATTGGTGGA
GAGGACATTGATTTTGAAGAAGCTAGACTGGCATTTTTCACCCATGACTCTGGTTAGGATAAGCTTTTCGTAACCTTTAT
GGCTCATTTAATAAAACAAACAAACATGCCTTAAGCACTTTAAAGAACAGTTTTACCCTGACTGGGAATACAACGACCCA
TACCAGGACTACAACATACGTTAGCGAAGCGACTTCAAAATCTAAAAGCACGTGGGAATAAGTCACATGATGTTATGCAA
TGAAACAACCAGAAACGAAAATAACAAAACGGAATTAAAATGGTAGCTAAACTTTTAGCTTTGGGTTGGCTTCTAACCTT
GAGAAATATTGAGTACTTCGCAATCTTGATTCTATCCCATTCTTCAATTAACTCTTAACTCCCAGAAGTGTTTCATCTTT
CTGTAATCTCCATACATTGTCCGCTAAAGAGGTAATGAGGATACTCCAACAGGTAGAACTTGTTATCTTGATCTAACACC
AGATTCTTTTACTATTTAACAAGGGAATGTGTTACAGATGGATCCTATATCATGTGAGTTAAAGGGTTAAGAAATACATT
CTGTAATTAAAAGTCACCTAATCTGAGCTTGTCTCTCAAATTACGTGATTCGATCCAACCACAAAGTGACAAAAAAATTC
TGACCATGTTTGTCTCTGAAACTTTAGAGATTGAATGAGATATTGCACTTTGATAATATTGTGTACTAATTAAATTTTGG
TTAAATGTTTCTCTTTTTCATGTAGTTCACTGCTTGTCTAAAATAATCCAAGGAACAGAAATATCTGAAGGAGGAGGCTG
GGAAGAAACTCAGAATTGGCTTATTGCACTGTAAGTCATTATGCACAAACATAAACCATAAATTAATGCACTGGAACTCA
GACTTCCTCaTCCTCGGAGACCCAGGGGCTGTGGGTCGATTTCAAGTTAGGGCATGAAGAGCCCCTGGGGACATTGTCTT
ACAAGACWAGTTCCAAACRGTCGCAGTCGTTCTGCCATCTGATTGGTGCCAGAAAATCTTTGTGTTTTTCTGCCCAATCA
GAATGAAGTGTGCTTTAGAGTCCTTTCGTGTGTTTCAACACGGACATATACTAGTAGCTCAGTTGCTCACTATGTTTGTT
TGGACTGTGCGACGGAGGTTTTCGTGAGGTGAATTGTTTCAGTCTCAGTACACAATGTAAGAGAAATTGATCAGATTGTC
CGCGAAAATCTTACCGGAAATTATACTGAATACTTTCGCAGGTATCACTAGCGAACGTGTTTTAAAGTTTGCAAGGTTTG
TATGAAACACGATGGTTGCAGTCATTGATATGTCGGTTTAAACGTTAAACAAGATTTAAGCTTAAATCTTTCTAGATTTT
TTGCATAATTTGCTGTGACTGAAACTTTGCCAATCCGTGAAGTTATTTCATCGCACGGGCCACGCAGGTATGTCGAGCAA
CTGGTACTTGCGCAACTTTCTGTGCCTGTCATAATTAAATATCATCGCTTGAAAAAAAAGAAAAGCTCAGCATGGAAACT
TTTGCTTATTAGATGTGGGTAGATGACCCTGTGCTACTAAGCTGACATTTGCAATAAAGCAAACCCGCCCTTTCTATCCA
ATATTTCTTGGTCTTTGGTTTTGTTTTTCTCTTTGAAAACAGCATTGGATGGATTCCCAATGATCAAAGGTATTTAACTT
GGTCTTCAACAATCGACAGAAGAGGTGAAATGACAATGACCAAGGGACTAGAAGAGAATCTGTGGCCTTCGTAGCTCAAG
AATTTCAACACTCTAGACAGCAGTTGAAAGATTAAACTCTTTCCAAACTCCTTCGGTAACTGCCCGAAAACATCTCTNNN
NNNNNNNNNNNNNNNNNNNNNNNNNNNNNNNNNNNNNNNNNNNNNNNNNNNNNNNNNNNNNNNNNNNNNNNNNNNNNNNN
NNNNNNNNNNNNNNACTTGCTGGAGGTCGTCCACGAACGTAACGTTCTCTGCAACCTTGGCCGTATTGTGAATGACTTTA
CTACTAAGATTAAATTTTGGCGCCCACAGAAAGCAGAAAGCTACGCAGGCTCGACATATCTGTGTAGCCCGTGCAACGAA
ATAACTTCACTGGATTGGCAAAGTTTAGTCACAGCAAATTATGCAAAAAAAATTGAAAGATTTAAGCTTTTAAATCTCG
TTTAACGTTTAAAGCGACACATCGATGACTGCAACCATCAGGTTGTATACAAACCTCGCAAACTTTAAAACACTTTCTCT
AGTGATACCTGGGAAAGTATTCAGTATAATTTCCGGTAAGATTTTCGCAGACAATCTGATCGATTTCTTTTACATTGTGT
GCTGAGACTGAAACAATTCACCTCAAGCAAATCTCTGTTGCACGGTCCAAAGAAACATAGTGAGCAACTGAGCTACTAGT
ATATGTCCATGTTAAAACACACGAAAGGACTCTAAAGCACACTTCACTCTGATTGGGCAGAAAAACACAAAGATTTTCTG
GCACCAATCAGATGGCAGAATGACTGCTATCATTTGGAACTAGTCTTGTAAGACAATGTCCCCAGGGGCTCTTCATGCCC
TAACTTGAAATCGACCCACAGCCCCTGGGTNNNNNNNNNNNNNNNNNNNNNNNNNNNNNNNNNNNNNNNNNNNNNNNNNN
GTCTTGTAAGACAATGTCCCCAGGGGCTCTTCATGCCCTAACTTGAAATCGACCCACAGCCCCTGGGTCTCCGAGGATGA
GACTTCCTAAGAAGTAACATTTTACCACCATCTTTCTTATTTGTTTGCTTGATCTTGATTTGTGGAAAAGGATGTTGATT
ATTATGCAACTTGTGACCTTGTAATTCTGAGCCCAGTCCATGTCTCTCCAATGTGATCTACTTCTCAGCATTATCTGTAC
AAAATGCAAGCAACTTGTAACTTTAAATAGTTTGCAGTCAAAGGGTCTAGATGTCTTGGTGACTGAAATGAAAAGATTTG
GCGGCAAACTCCAAGAAGTTTCTAACCTTGGATTTCCCAAAGCCCATCTCTTGTCTTTTGTTCGTCATCAAGTTAAAAAC
CTTGCAAACTGTAATTCTTTGGGTCAATGCATGTGGTGCAATTTTGATACTTTAGGTTAATAGTCTTTTGACTAGCAACT
AACCTCCCGGTTAGCTCAATTGGTTAGAGCGCTGGACTCTTGTGCGGGAGGTCAAGGTCGATGGTTCGAGCCCCAGCCAG
ACCAACACTCAAGGTCTTACAATAACTGAGGAGAATGCGCGCTACCTTTCTATAATATCAAGAAATGGATAGACATTCTA
GTCTTCTCGGATAAGGACGAATAAACTGTAGGTCCCGTCTCCTGGATCTAGTATTTAGTTAATTTGGTTAGGCAGGGGAC
GTTAAAGAACCCATTCACAAGATTACGGTGATGTGGTCTGGCCTGGTTACTGGGAATTGCGGGGAGGGGGCATCTTCAAA
TAAGTTTCCTTCAATTGTTTGTAAACTGCTGATGCGGTCCGGCCAAAGTCCCCCGAAAAGTGTTGTGATGCGTATTTGAT
CATATCCGCATCTTAATTTGTGATATATAAATATTATATTATTATTATTATTATTAATTTCTCCTTAAAATATCACCCAC
AAATCACACATTAAGGTCACAAGAATGAAGAAAAATGATCACCAACTTAAGAAACTCCAAATTATCAAACAAATTCTTTG
TCACCATCTAAGGAAATGTGTAGAGAGAAGTGTCTCCAAAGAGTTGGGAGAATAGCTCCCAGTGTTGTGATCTGGTCTTA
TTGTTTATAGAGGCCTCCTCTTAAACCAGCCTTAAAGGATAAGTGGACCAGCCTGTCTAAATAATTCAAGAGAGTGAAAT
AAATAAATAATTTTTTGGTCTTTTATTGTCTGCAGGGGAGAACTACCATCTGTACAGCAAAGCCACGTGGCCTTTGCACA

ACTGAGACATCCTGTAAACTTGGGAAGAACACTCGAAGAGACTCATTTGGTTGTCTTGGTGCTTGCTCCTAGTAAAGCTG
TAAGTTACCCCTCTTAATTAAAGGCAAGCGTGGCAAAGTTAGATGATAATTTATTTCTCTAATTAGTTGCTTAAGTCTTG
GCAAGAATAACTTATCACAGGTGAGGGCTGTGAACAAGTGAGGTATTGTTCTTGCCACTCAAACATAACATTCAATCGTA
TCTTTTTGCCACAGCATAATATCATCTATATAATTTAATTTGTTTATTGGTGAGAAGATGGCACGAAGTCATCAAGGAGT
GTAGGACATGGTTGAGTAGTATGTAAAAATGCAATGTCAGAAAATGGGATTGTAATTAATGAATAAATGAAATTGAATAA
CTATTTTGTTAGTTTAATTTATACTCCATTACTAGCTCTCCTATTGTAAAAAAAAAAACCTGATTTGGGAATAGTTTCTT
CTTGAAAGTTTTCCATTGTGATTTTTCTTACTATTTTTATGCTATTCAGAAAAGCACAAAGAACTCACTGGAAACAGGAA
GAACTTTTGCAACATTATTGTCTGACATAGAGATAAGACAGCTGCTCATTGAAGCAGAAACAGAAGAGGAATTCAAGGTT
TGTTCAAGTTATGGTCATGAAAATGGACTTCACTTTATAGTGATAGCTTATCCTTTGTTTATTGTGAACAATTCAAACTA
CAGCATGTATTGTTTTAATGTATGGTTTTCATTTACAGAAAGTACTGTCAGATCGCAAAGGGATGCTATCTTCACAATTA
TTGCCAAAACGCAAATCTACCATAAGGCAGTTTGGTTCCCAACCTAAGTCCTCCTTTATCCATCAAAGAGATGATGAAAA
TGAAAAGGTATACTGTTAACCAAGATGCCGACAACTTTTCTTTTTTTTTTTTTTGTACTCTAGTGTAGAGCATTATTTTA
TATTTTTGTGTCACTTAACCCTTTTTTACTCCCAAGATCTTATGGGTAATTATTTGTACCATTTGCTGTACAGTTCTTGG
GTAAAGGGTAAAGGTTCATTTCACAAATAAATACATTGAGATACAATGGCAGCGAGTAATAGAATGCCAGTACCNNNNNN
NNNNNNNNNNNNNNNNNNNNNNNNNNNNNNNNNNNNNNNNNNNNNNNNNNNNNNNNNNNNNNNNNNNNNNNNNNNNNNNN
NNNNNNNNNNGTACAGGCCTGAATTTTTTTCAGGTCTGATTTTCACTATTGCTTAAATAGTGCTTTACTGCGAAGGATCG
TGCACATACGTAAGTACAGATCGAATGGAAGTGCAATAGAATAAGACTAATTCACTTGTTGGGAGTCTCGCCCTCTTAAG
CTGCACCAAAAAATACAAACGTTTAGAGGCCTTGTTTATAACCACACTAACATGCTCGTTCCATGACAGGTCATCACTTA
TGGTGACCCCAAGGAGTTTTGCAGACTTTATGGTCTCCACACCTTTCCCATTAACTATAATTGGAGGAAACCCCTGCTTT
TGTTTACTGAAAGTAATTCAAAGCTCTTTACATTTATCATTGTTCAGTTGTACTCTATTTTTTTAGAGGACCATTCCATT
ACACGGTCAGCTATCCTTTGCGAAATACTTGCCTTTCCTTTGATGACAACTTCTGAGGTCGTAGTGTCATCGACATATTT
CCACATTTTGGCATTTATGCCACTACTTAATTCAAGATCATTAATAAGCACAAGAAATAACCACGGCCCTAGCTTTGTTC
CTTGTGGGACACTGGACGGAACTGACTCCCACTTGGAAAAACAACCCTTTGCAAGTTTAATTCACTGAAAACGGCCCGAT
AAAAAAATCTGTGATCCAATTAATTATCTGTTTGGGTATCTCCAACATGGTCAGCTTCCTAACTAAAATGTCATGATCTA
TAAGATCAAAAGCCTTCCTATAATCGTATAGTGTGGCTCTCACGGTGGTGCCATTTCTGTCAGCTGCTTGTGCCCATTGG
TGGTTCTTCTAATATAAGTTCTGATAATTTGATGTTGGATGATTCCCAGAGTGATAATTTTTGCTATTCTCATCACTTAT
CTGCTTGATATTGCAGTGATGTTGTAAGGAGAAATTCTATCTTGGTCCTTCAGTTGCATGGACAATGCTTTATATTCTGG
AAATACATATATCTTATTAACCAAGTGCGAGGGCTGTACTGGGAGAATATTGGTCTGAGATCTTGACAGTACGGACCTCG
GTCCGTACAAAAAAGACCGAGGGCCGATATTCTTCCAGTACAGTCCCCAGCAAGCTTGGTTAATAAGAGATTTATTATAC
GACTGAAGTTAAAAGGGACACAAGTGCGGTATCATTTGGGACCATTCGGGACAGTACCCAGTCCAATACTGAGAGAATAT
TGACCGGCAATAGAGCATTTTGATTGGTTGATTTTAGTTATTGGCCCTCTGACTGCCCGAGTCGTATAATTAATAATACT
TACCTTATCCGTCAATTTGACAGAGATCAGAGTTGTTCTCATTTGGAAGGGGGGTCATACGTGACTTTCGAAGGAGATGG
CCACATTATTTATCTGATTTTAAAGATGGTAAGTTGATGTGAATGTGCCAAATTCATTACTACAGAAACTTGGAGTATTT
TTCCTAGATACTTTCTTGTATCTTTTCATCTGTATGGCTTTTTTCTCCAAATTTTTCAGAAAATAATGCAACAGGTCTGA
AGAACACATTTCTGTTAAAACTGTTATTGATTAAATCAATGATAACGTGACCGTAAATTAATCAAACTAAATGTTGTTTT
AATTATTTTTGGTCTTATTGTTGTGTTATTTAATGAGTTATTTTTTAAGTGGCTTTCTTAAACTTAATTAGATTTTCAAT
TTTTGAGAATGGTTTCAAAGCTTACCCTTACGACTTCCGTCAGTCCATTTTAGAATTATTTAATTTTCTATTTTTTAATT
TTTTGATTGAATAGGAATAAGAGGTCATCGGACCATTCCTAAAGTGATCTCCACAACACTTTTCTTATACTTTGCCTGTA
TTCTGCCATCAATTGCATTTGGTGTTCTCAACAGCCGCAACACTAGTGGAAAAATTGGTAAGTGGGCTAGGTTTGTAGAG
GCTGATAATGNNNNNNNNNNNNNNNNNAATGGATGGAAATGTTATGATAATTAAGACTCGTTATTTAAATAGTTTGGTTTT
TGGATGGCTGGAATTTGTTAGCATTAATAGTGGATTTTTACAAACCCTGCAATTCATTTGGTACTTCATGAAATTTATAC
TGTCAGAAATGACACAAGTTTTGAATTTATTGCATATATTGTTGTATTGAGTCCATAATAACCAATCAGTGTCACATCAT
CAACAAGTTTAATTGAATTTTAACATGCAGAAGCATAGAGAATTTGCTACAATGCATTACAAAATTTTCCATGAGGCTTG
TAGCCTAAAGTTTGACCCACATCAGTCAGCTTACACTTTGTTCTCATTGACTCTGATATTTAAAGCTCACTTTTTTTAAT
GATGACATAGTTGTTGACTGCTCCACTCATCAATCTCTTGTAGATGTTCTGAAAGTTATCATTTCACAATCTGTGGGAGG
AATTTTGTTTGCTCTGTTTGGAGGACAGCCTTTAATTGTTTTACTTACCACAGCTCCACTAGCACTTTACATCAAAGGTG
ATGTATAGATGTTGGCATGGTAAACTAATTATTTAACTTCAATTGGTAAACTAAATTGTAACAAGGTTTAAGCAGAAATG
AATTACTTGAGATAAAAATCCCAATTTAAAATTGATAGTATAATTTGCAAGCCTAGTGTTGCCACTTACAGTTAAACTAT
ACATGTCAATAATTAATGTTTTACATTAACTTCTTTTTTCATCCTTAATCAAATGATATTTGTTAATCTTTTAGTTATTT
ACAACATTGCGGCAGACTTTGATTTGGATTTCTTTGCCCTATATTCTTGCACTGGCCTTTGGAATTCATTCTTCCTGTTT
ATCTACTCTACATTTGGTCTTTCCCAGATAATGAAGTGGTCAACAA
>6791424 14047 18299
CCTGATTGGCAATATAAACATGAGTAGAACAGGAAAAACCATCTTAAGATATGGCAATGGGGCAAATCCAAATCCACATA
GAATACCAAGCTGTAAGAACTGCAGAGCAGCGTACAGGTGCATCTTTCTCTGAGGCACATGTCGAATGTAGTGGTTTGGT
GGATAGGAAGCCTATAAGATTTAATATCTTTTAATAAATCTAATATTTTCAGATCTTTTAGAAATGAATTCAAGTATTAA
AAACTTACTCATCATCAAGAGAGGATTGTCATAAATTGCAATAACCAAATTTGATTTCTTATGACATAGGAGTTAAGTTA
ATGAAATAATGTAAATTTGTGTCCCAATCAACATGGAGTTGAACCATAAGATCAAGAGAGGAAAAACATAATGCTCTTTC
AGAATTAATACAAAATTTGATTCATATGAAAAGAGACAGTATTAGCACACACAGGGTTGTGCTCTTCAGAGAATTACCTA
GACACTCTAAATCTGCAAAATCCAGGGCGCAGTGAAAAATTGCACTTTCAATAACAAAGTGGCACAAAGTGTCCCACAGG
CATATCATGTAACTCGTCTGTGACACCTAAACTCTGTTAAGGCACAACCCTGTGACAAAAGAGTATGATTTCTACCAACC

TGTTCTGTGACCAACAGCAATGCTCTTTCAAACATCTGGTTATGATATAAGGAAGTCATGGCCATAAAAAGAAACAGGCC
ATCAAGGACAGGTGTGGGGATTAGAGTGAGAGGAATGGGTAACATTAACAGTGATAATGCAATGAGAGTGCTGGAGAGAA
ACCCAGTCAGTCTTGTTTCTCTAACTTTGACAATGCTGCAAAAGGAAAAAGAATGTATGGACAATCGTCAATTCTTATTC
GTTTTTATTCTCAAATAGCCTTCAGTTACTTAACCAAGGAAAAGCATAAATATTTGTAATCCACAGATAGATTGCAATTC
AGTTGAAGAACTTTTCAAAGTTATGACTAACAAAAAATTTAAGGGCAAATGAAGTCAACTGGCTTTTGAAGTTCAGAGAG
TGGCTGCTATGCAAAATAACTGTTAGGTATTCACATTTCCCTCTGCTCCTTTTTCACATTTTCATGCCATAGTACAAGTG
CACTACCATAATTTACATAATTCATGCACTTTTCTGACAACCTTTTAACTGACAACTGAATAAAGAATTCTTGCTATGTA
CACTGGAGTTATCAAACTCACATTTCAAACACATGCCTTCTGTCTACTCTCTCTTCAACATCTGCCAGTGCCCTGACATG
AAATGGCGAGTGAGGTAGGGCAGCATGTACCCATGGCAGGCCAAAAATGGATAGACAGCCATTGACTACAGCTACAACAA
GTAAATCCCAATGATATGCACTGCCTTTCTTCAGTCTAATAAAAAAATAAGTTAAATTTGATGACTCCTCAGCACTGCTT
GGTAGATAGTAAAAATGACTGGGTTAAAAATAAGCAATTAAATCATGTATTGAAAATCCAGCTGAGATCAATACTATCAT
GCTTTGCTATTCTTAATAGTCTTAATAAAAAATCTGAGGCTTTGTAAGGCTCATCAACTATCTGGTAATGCAATCCTGTG
TACTCTTTTAGTTATAAATGCCTGCTGTTGGCTTGTAGGTCATTAGAGTGAAGCCCTGTCATCAATGTCTCATGGACACT
TGCACCTTTTTTCGAGTAAAAGGTTTAGTGAACACACTAACTTTAACCAAATGAAAAACAAATTAAATAATGAGAGCAT
TGCCATGATAGTGGGCAGAAAAGAAAAAAATGTGGAATGGACTACAGCCTAATCATAATGTAGGATCTCTTTATCAATGCT
ATGTACCTGTTTCTAGGATTATTAACCATGGCACTTGAAATATTCTAATCCATGAAAAACAACAGAGAAAGGGAGAACCC
TAACCCAAGTCCTGCAAACACTGCACCCCAAGTGAGCTTGGGCAATGAAGCAACAGTAAATGAAAATGTGCTGCTTGATT
CAAACTTGTAATCTGTGAAAAGTCATGAAGAATAAGAGAGTTACCACCAAACTAATTAGATATTGGGTAGTCTTTCTTTT
TTATTTCAAAACTATGAACTACCAAAGTAAGGTTGTTTTACGACAAGTTTTTCACTATATTACAATTGTTATGACATGA
TGATAATAATGTGATCTTTAAGTAGAATTCCAGAACCTGATAGTTATTCTTCACTATCACTACATCAGTTCTTTAGAGTA
GAATGAGTAAATATCAAAGAAATAATAACACTTCCTGCTCTTATTGTGCAGTACGTACTATCAATATCTCGCACAAAAAA
TGAAGCAATAAAAGACATGACAAGTACAGCCACCACAAGTGCATAATCAGCCAGTGCCTCTCTCTTTCCAGCATTGAGAA
AGGGGCTGAAAATTGAATTAGCAAGTCCATCAATTAATAATTGAACATATTTAAAACAAAAGCCACATTTAACTGCCTTA
CAGAAACAATTTCATTCTTGGTAAAGAATGCTAAAGCTACAAAAATTGTATCTAAGTTGCATCATGCAGTGCTTTGCTAA
AAAGTTCTAGTTGCTAAACTTTTGTCTTGTTATAAAACAGCAAATCATTCTCATGATAGAAGGACATTTCAGCTTTACTC
TCGTGCAAATTAATGTTTAAAAAATTACCTTATTGTTACTAACCTCTTGCAAAAATTGTAGAGTGCTACACCAATCCAAA
CTGTTCCTAGGCTTGTAACAGCATAGGAGAGACCTCCCTCTCTGCTACAAACTGATACATTTGGGACCTGATCATGAGTG
AAGTTATTAGCAATCCCCTGGCCAACAACACAAGATGATGAGTTCCAGCAGGAACTTAAACTTGTACAGTGAGGACTTGA
GTATTCTGTTGAGAAGATTTAAGTGAAAGTTACAACTTACCAAACAACTCCCATAATATTCCTACTAAAAAAAACTAGCA
TATGAAAAAAGACGATTATGACAACACCATATTGGTCATTCATTCATTATTACTTCACTGTCAAAAACTCTGTAACCCTT
AAAAACAATAAGCATTTAATTTCACCCAACAGTGTCACCCCTGAATCCAACACTCAAAATAAAGGAAATGATCTTAAACC
CAAGAAGCTCTTGATTGTTAGACAAATCTTCCCAGTAAGTACATTGGGGAATGAAAAGAGAAGAAGCAGTATAGAGAATA
ATCATACTGATGTTAGGATGTAACTTAGGGTTAATTGTGCTATAGTCACATAGTTTACAAATACACAAACACACACACGT
ACACACCTACATAACATCGGTTAAACATCAGGCAAGCATATTGAAATCAATTTTGGCCGGCAGCATGCCAATTTCATTCA
CAAAATTTCTTAGGAAAACTTTGGAAACAGCCCTTTCAGGCAAAATGTATAAAGAAAAATTGAAACATTAGGGAAAAAAA
ACAAGGAATCCTTTTGTCTTACCCTTAGGTCATACACTAAATCAAAATAGTGATATTATTTCGAAATACCATNNNNNNNN
NNNNNNNNNNNNNNNNNNNNNNNNNNNNNNNNNNNNNNNNNTCCAACACTCAAAATAAAGGAAATGATCTTAAACCCAA
GAAGCTCTTGATTGTTAGACAAATCTTCCCAGTAAGTACATTGGGGAATGAAAAGAGAAGAAGCAGTATAGAGAATAATC
ATACTGATGTTAGGATGTAACTTAGGGTTAATTGTGCTATAGTCACATAGTTTACAAATACACAAACACACACACRTACA
CACCTACATAACATCRGTTAAACATCAGGCAAGCATATTGAAATCAATTTTGGCCGGCAGCATGCCAATTTCATTCACAA
AATTTCTTAGGAAAACTTTGGAAACAGCCCTTTCAGGCAAAATGYATAAAGAAAAATTGRAACATTAGGGaAAAAAAAAC
AAGGAATCCTTTTGTCTTACCCTTAGGTCATACACTAAATCAAAatagtgatattattTCGAAATACCATAACATAAGGG
TTTGTGTTTTGTGCATTCAACTCTTTTAAATCAACTTGACCTGATACCCTGGAAATGCACCACCTTGCCATTTTTGCATG
TGTAGACATTTAACTGCTGTGTTCCCAGAGACATTCTCACCAATCCAGAATGATGAACAATAACTTGTACTTTTTTTAGA
GTAATTATTATCAGTACCTTTGATGATGCTACTGATGGCATCTACTGTAAATGCAAGTGAGATGAACAAAGCAAAAATTT
CCTCTGTCGATC
>scaffold_7 1026729 1045347
GCTTTATCTTTGATTCAAGGGTGACACTGTATGGAGAAATTAGAAGCCAGTCACTCCTAGTTATAATTGACTGGTTCTAT
CCTGTGTGCAAACCTTCCATTTTCCTTTTAAATCAACGTCCGAGTTCAAGCGAAATTATAAACCCGCTGGAGCTAAATTT
TCGAGCTTTTGTTTCAATAAATTCATATGACTGCTTACCACGCGAGTGAAAATGTGAAAAAAGAATATATCTCGTAAAAT
GAGTAGAACTTTAAACTCAGRAAAAAAAAAAAACCGTCTTATGTGAGAGGTTTTGATATCTCAGCTAGATGGCAAATTCAT
TCATTTCACTGGAAATATTCTGAGAAATAAAAAATCGGACACTCTTCGTTTGTTATGATTGAAATCGTCTTCTGGGTATCT
ACATGTTGCAAAATAGTAAATGAGTTAAAATCCCTTGTGTGGGATTTCTTATGTTCTTCGATTAGCTTCACAAGTCATTT
CAAATCGTCGAGTGTAATTCATTCTTCTACCTGACGAAAGAAAAAAGAAATAAACAGACAAAGACTGCATGTGGCTACGA
GTATAGCAAAACTTTTGTAGCATTGAAAAGAACTGAAGGTTTTTATAATGAGGTAGTGTTGCAAAATATTAGTCTCACAT
GATAATATTTACAATGACTATAATGATAACAATAATAACACAATAACAATTCAAAGGGATTGGCTCGAACGCCTCATGGT
TCCCTCATAGTTTCCCTGGGCTCCAACAATGATTATCTAAGATGCTTCTTTTAATTTCGCCTGTTAACTGGACACAATTT
TTTCATTTTATTGCAAATCCATTTTATGAGACTTTTGTATTGGGAGTCGATCCGCCGATCCACCGTTCTCCCCGTTCTAT
CGTTCTGTTGAATAACTCGAGGTATCATGTTTCGCAGTTTGTTCACTTCGAAAATTTAGCGGCCCTTTTCGGCAAAAACG
CACGTGGGCATGCACTTAGGCACGTGCGTGTGCATGCACGTGCGCGCACACGCACACACGTAGGTACGCACGTCCGCACG

CAAGTGCGAGGCGAGTGCTAGGCGTGGGGCGGACTCCACTGGATTGATAGCTCTTGATTTCTAATTTGTTTCCTTTGGTG
CGTAGGCATGTTCCCGCGCACGCATGTACCCTACGCACATACGCACATACGCACATACGTGCATACGCACATATGTCATG
ACCAAAGGAAACAAAAATTTATCAATCAAGAGCTATTGATTCAATGGAGTCCACCCCACGCCTTGCACTCACCTCGCACT
TGTCTCCACACTCCTGAAAATAAAAAAACGGGAGAAACTGACGCTGGTCCAGCAAGCTAGAATTACTTCACCCTGATAAA
ACCTGGAGAACTTTCAGCAGAAATGAGACACTAGGCTACAATTTGATTTTAAAAATTTAATTTATTTACTTATTTAAAAT
ACGTTTTACAAACCTGTTGATTAGAAGGTCCAGCCCCCAATGCTGATTCAGTGTCGCTCATCACAGGAGACGAATAGGAA
GCATCCTACAGCCACAAAAACAAAAACAAAAACACAAACAAAGGAAAGAAGTCTTAGAGCATAATGCACCATAGACTCAC
CCTTTATTGACGTTTTGCATTACATTTACAGAGGTATTACACTCAACTTTTCGAAATAGGAAACATTCGCACAAAAACTT
AAGGAGAGCTATTCTGGTAAAATACATGGAAGATTATTTAAAGCCACCATTATATACAGGAATTTGGGACCCTCGCAGTC
AAAGAGGAGAGCACAAAACATCATGATACTCTTTTTCTGACCTTTTTACTCTCGTGAGTGACCAAGACAGAGTTTCTCCT
CGAATTATTAATACAAAATCAAGCAGACGAGTGATGAGAATTAAGAAAAGTGTATATTAGGGGATTATTAGTTGATCCAA
TACCTAATACCTCGAACTAACATCACAAGAATTGTATTGTAGAAAGTAAAGAGAATTACCAATGAGATCTTTCGCGTGAA
AGGGTTAAATTCAAATCCCTAACCTCACCTCAGAGTGAATTCCAACAGCGTGTGTCACTGCGTCATGTACAGACACAAAC
AAATGTTCTGTTCCTATTCTTGCTGTAAAGCCAGCTTTCTCTAACATTTCTCGTATGTGATCTATAACAAATAACACAAA
GACATACATTTTCCCGCCACTTTTATAAAATTCAAGACTTCTTTACATATTTCTTCGTTTCACTTTTCACTATTCTACGC
CCAAGGTTTATACAAAATTCACCGAGGCCTCAGCGGAATAATAGCTGTCAAGGGTTTGCTCCCAAAATCGGAACTTCTCC
GGCCTATCATCGCGAAACAATGTAATGCTGGGAATTTACGTCAGTTACAACTTCCTGATAAAGATCTCACCTTCGAAACA
AGCATCTGTAGAAGCTGAGAGTAGCTCTAAATTTCCATACTTTTTCGAAAATTAGTTTGAAAATTTGCCTGCTTTTCTTT
CTTTTTTATTTTTCGGCCACATAATAACGGAATTGACAAAAATTGTGAACAGTAAGTAATTCTTAACAACAACTTACATC
GACAGTGCGCCAAATAGAATTTTACTCCAATCTTTTCATATTCTACACCAAGCTGAAATGAAAAGGAAAACATAATTGGT
TTCGCATCAAATAGTGACGAAGAGTACAGTCCTGGCTGGGTCTTTTGTTGTGTTCCTGGAAAAGGCAATTTACTTTTAAA
GGCTCTAACCACCCAGGACCGGGTTGTTCAAAGGTTCGTTAACCTAACTGTGTGACATCCATCACACCGTAATCTCGTGT
GTGGGAAATTAACGTCTCCTACCCCAACCCTGCAGGAGACGGGACCTCCATTTATTCGTCCCTATCCGAGAAGACTAGAA
TATCTAACCATTTCCTAATGTTATAGAAAGGTAGCAAATTCTTCTCAATTATTTTAAGACCCTGAGTGTTGGTCCGGCCG
GGGCTCGAACCCTCGACCTCCCGCACAACAGTCCAGCGCTCTATCCATTGAGCTAACCGGGAGGCAGTTAGTTACCCTTG
CAAACAAACCCACCACTTGTTCAAAGCCAACAACGAACACTTAAGGCCAGGGCCGGGCTGTTCAAATCAGAGTTAAGATA
ACCCAGGGTTTGTGTGAAATCTGATTTCAGATCTGAAAACTTTAAGGAAACTTCAATAGCATTCTTTTTAAGGATAAGTT
GATGGTTGAATGCTCTAAAAGTAATAAGGTAAAATTACACCAAACAGGCTTCTGAACAAGGAATAAACCCGGATTCAAAT
TTAACCTTGGGCTAGCGCTAATCGAATAAAGGGGGTAAGTTTCCAAAGAAACTGTGGTGCTACGTCGGTGGGGGTAACAA
GGTAATTTGGTAATTTCAGCTAAGTTGAAATTGTAAGTTGGCCACCGTGAAGAGTTAAAAAGCTGACGTTTCGAGCGTTA
GCCTTCGTCATTCGCTCTGACGAAGGGCTAACGCTCGAAACGTCAGCTTTTTCACTCTTCACGGTGGTCAATTTACATTT
TCAACTTAGTTGATACTACCAAATTACCTTAGCGCTAATCGGCCTTGGAACAATTGCGCCCAAGGTCACAAATGCTGTGA
CAACAACTTCCCACCTCATGAGACAAACGATTGAACGTTCATTTTTCCATGTAACCACAATGGAACAACATCCTCAACAA
CAATTTCCTACCTGCAACAGAGTGTTCACACCTTGGGTGTCAATAAAATTGAAAGTGCTAGCGTCAATTATGATGGCATG
AGTAGGGATTGGATCGTATCCTGAGGGACTCCGCTGATTACCAAGATCATTAAGCGAAACCTCCACATCCTGAAAATGAA
AAAAATATGATTAAAGATAACATTCTGTAGATTTAAATATCAATATTTTCCGCCAGTGCCTGTCAAACAATTCTCATAAT
TTTTGCTCTTCCTCCCAGCGAATGAAGAGGGGCTAACGCCGATAAACATTAGCTTGAGAAACCCTCTACGGTGGCAAATT
TACATTACAAACTTCATCAATAAAACCACATACAGCCCACAGTTTCTTTAGAAACTTGCCCCCTTCATCAATCCACTCTT
TAACCCCTAAGTGTCTGGCTTCTGATTTCTCCTTACAAAATCACCCTTAGACCAAATGTAAAGGTCATGAGAATAAACGA
AAAGATCACCACTATAAAAGCTCTGATTGACAAACAAATTCTCCTTGTTAGCACCATAGAAAATGAGAAGAGAATGGTGA
GGAGAATAGAAATACCAATGTTAGGGAGTACAGGGTTAACTTTAACACCCCTCCCCCCCCCCCCAAGTACTTTTCTCACA
TAAAATTCTCTGGTTTTTCCTTTGAGTTCCCATTGACTCCTCAGAATTTCTCTCTTTTCTCTGACTCCTGATTGTCATTA
TTTTGGTTCTGGGTGAACCCTTTAAACCCTAAATATGACCAGCATCTAAAGTTTAACCTAACAGCAACACAGCTGAGTCA
TTCATTAAGATCGTGAGAATACAGGAAATGATTGACAACCAAGAGAACTTTAATTGGTAGACAAATTCTCCTTCCCAGTA
CCAAAAGAAATGTACGGAGAAGAGTATGGAGAATACGTATGATGCACGATCCTCGCAGTAGAGAGCGCTATTTACGCAAT
AGCGAAAATCAGACCTGAAAAAAAATCAGGCCTGTACGGGATTCGAACCACTGACCTCTGCGATACTAGTGCAGCGCTCT
ACCAATTGAGCTAACAAGCCAACTGGGAGCAGGCCGTTAAGTTGATTCGTAATAAACCCATGATGATAAATCAGATATGA
TGAATATATGAAGTTCATATATTTTGAACTGCGGTTATTGATACGTATGATGCACGATCCTCGCAGTAGAGACCGCTATT
TATGCAATAGCGAAAATCAGACCTGAAAAAAATCAGGCCTGTACGGGATTCGAACCACTTGACCGCTGCGATACTAGTGC
AGCGCTCTACCAATTGAGCCAACAAGCCAACTTTTATTTATATTTTTTTTTCAGGTCTGATTTTCGCTATTGCATAAAT
AGCGCTCTCTACTGCGAGGATCGTGCATCTTACGTATCTATAACCGCAGTTCAAAATATATAAACTTCTTACATTCATCG
TATCAGTATGGAGAACACAGATACTGATGCTGGGTTTAAACACCACTCAGACAAACTCAGCAATAAAACCCTGTTCTCAC
ACACAACTGGGAGCCATGCCAAGCCTACCATGCAGCCCGACCTTTATTATATCAACACAATGAACCATTAGGGTTACAAC
ACTAACAAAACAGCCGAAAATGAGCCTAGATACAAAAGATTGACATGCACGAATAACTGGAACTAAGGCACTACTTGAAA
TCCTATATCCCCAAAAACATATGGCGGGTAGGTGGGTGGGTGGGGAAGGTCCGGCAATGGCAGAAGGCAACAAAACAAAT
GAACCTTGAAATGCCATGTGACCTGGAGACACCCCACACACTCGAACCCATTCTCCCAGCTGCGTGTGAGAAAAACGTTT
TCCGCTTCTTTCGTCCCTCAGCCAGCGGAAAACCATACATATAAAGTATGCATATTATGTGTATGTAAAGATCACTAAAA
CTCTACCCCATTCTCCACAGCCCTGTTTATTGAAACAGCAACAGTCCTTGTTATTTCTATTGCCGGGCCCCCAGTATCCT
CCTCACGGTGTCTATAGCGCACTGCTGAGCTACCGAGGCGGCTGCGTTTGTTTGGATTCTGTGGATCAACTCCAGTCATT
TCTATCAAAGTGCTCCGAAAGTATTCTGAATTGGCGTAAAACATAGCAGACTCAAATCTGAAAATTTTTATTCCAGGAAT

TGGCTGTGCCTGCGGAAATAAACATGAAAGTCAATAATCACTTAACAATAAATCTCTTGGACCCCATCTAACCTGATGAG
TGGAAAAACAGACTTCCCTGAAAAATTTTCACTTTACGACTCCATGTTTATCTCCCACCCCCTACCCCTCGAAAAACACA
CAAATTCAACTGTACAGCAGTCAGTCTYCAACTTACTGAAGGTATCCTTTGGATGTCTCTGTAAATTCCAGTGTTGGGTA
TCTGACCAAGAAGGGAAGCAGGAGGTCTGGAAATTAAAAAAAGAAAAAACATGAAACAAAAACAAAAACAATGCAATGAA
TTATGACTAACTTAAAACTTTTCAGTCATGGAAAGAATGGAACGCCCACCACTGGGTAAGCTGAAAATCTAAACTATACC
TCACAAAATTAGGGGAAATTTTGGGTTAGGGGAGGGGTACGTACAAAGTTGCTTGAATAACTGATAATGTCAATAATAAA
TAATAAATAAATAAATAATTTAGCAATGGACATTCCACTGGGTGGCTCCTCTTGTCCACTGTTTCCAGATCGAATTGGAA
TTTGGAATGCTGTTTTTTTTTTTTTTGCAGAGGGAGGAAAACCGGAAGACCTGGAGAAAAACCCTCGGAGCAAGGACGAG
AACCAACAACAAACTCAACCCACATGTGATGCCAGGTCCAGGAATCGAACCCGGGCCACAGTGGTGGGAGGCAAGTGCTC
TCACCACTGCGTCATCCCTGCTCCTCAAAATTGGCCACTATATAGAGTTTCTTCGRCTGACCCTTTTGAGTGTTAGCCAA
CTGTCAGAGCAAATCGAGACGTCAGCTTTACAAACTCTTTATGGTAGTCAATTAACATCGTCAACTCAGTTGATAAAACA
AAATTATCTTGTGAAAGTGTAGTGTGATCGTCCGGGTAAGTGTAGTCTTGAGAAGGACTTTTGCTGATGGTGGTGACTGA
TGTTTTGACAACCTGTGCGGAAGTCATCATCAGAGTCAAGTGAATAACAACTATTCACGATGCAGTGCCACATTTTTTTT
TTTAGAAAATTACACCCCCTTTATTCACATATTAACAAATGAAGGTGATTTCTGGGTATTGTGTGCTATTGGTTAGACAT
TCTAGTCTTCTCAGATAGGGATGAATAAATAGAGGTCCTGTCTCCTGCAGGGTTGGGGTAGGAGACGTTAATTTCCCGCA
CACGAGATTACTGTGTGATGGATGTCCTCCCCTGGGGTGGGGTGGGTGGGAATAGTAGAGGGCCACTAGGGAGAATACTT
TCAAAAGTAATTGGTGTCACCCTCTTAAAATAAAGGTTATTATTATTATTGGCTTTTGTACACAGTCAAGTAACAAAAGT
TAGTTATTCTGGGTTATTAGAATATTATTAGTATTATAATAATATTATAATAATTATATTATAATATTATTAATATTATA
ATATTAATATTACTATTATTAATATTATTAGTTATTCTGACCTGGATGACTTCCAGATTACCACTAACAATGCCATGATG
ACACCAACTCCTAATCCAATGTCAATACCAAGCAGTACCACTCCTAACCAAGCAGCAAACCAAACAACCTAAAGGGAGAC
AATTATTGAGAGAAAGCAACCATCTTCATGACTATGAAACAGTTAATTGTATACTTGAAGATCTGTTTTACTGCTTAAAT
TTGCAAAAGTTAACAGTAKACCTACAAACATGAACGCTGAGAATAACTGAATATCAATTATTGAAAAATCATATTTATCA
TTAAATTAATTATTAATTGTCATCTATTAATTAAAAATCAAAGAGTAATTATTAACAATCTCATTAAAAATTGAAATTAA
AAGAAAAACTATATTTATTTTACAATTATTATAACAGTAATGTTGGTCATACGATGATAATGATTAAAATGAAGAATGAC
AATTACAATACTCAGAAATATTTTAATCTTCATCATTTTTAAAGAACAACAAGGATTTCAGTAAACTTACAGCATCAGGC
TTACACATTCTCCACAACTGTACCAGTCTGCTAAACTGTCGAAACAACCCCTTTAGTGCAACAACTACTACAGCAGCCAG
TATCGCCTGCCAAGTGAGAAAACAACAAAGAAAAGAAAAACAAATTGTTTAGTATTCTACAACATTTTCCACAAAGTGTC
AAGTGAGGGTAGAGAGGGACACAGGAGGATGTAAGCAAAGGTGTCTCAGCAGTGAGAAGGCTTGTGCTTCCAAAGCTGTG
ACCTGGGTTTGATTCTAAATTCCAGTGCCATCACATGACTGTACATGTATGTTGTTATTCCTTGTCCATGCTCTTAAGAC
TTTTCTTCTTATCCCCTGTTCTCTCCTCACACCCTCCATCTCTCCCTCAAGACTAAATACCCAGCACGGAGCTTGGTGAG
GATGAGGCCCAGAGTAAYAGGCATCATCACCTTGGAAACCAGGAACACCCAACATTCTAATAATATTATGCAACAAACAA
CTAAAATTTGACATGATCATAGTCAATGCACAATCAGTATCATGGCATTACAATAGAGCAAATCTGAGCACATTTTTCAG
ACTGTGAGTGCACTGCAGGCAGGGTAAACTGACCTTTGGCAAGTGATAGAAAAGAGGTGCTATAAAAAGCAACACCATGA
TAATGACTACAACAACTGGGATGCTGCAGAGCTGCAAAAAAAAAAAAAATTCTATACATGAAATATGTCACTTAGTCTGTT
ACATGTAGTTTCTAGCAAATCTAATGTACCAATCACTTTTTACAGACAGCAATTCAAAAGTTATCATCCACCATGTCCTT
GTCCCCTAACTGCATAGAGTGTTTCTTTGGGGGAGTATTTTGTAAGACATATTGCCAAATAATGGCTGACTACAATAGAA
ACATAGTAACCCTGTATGTTTAGCTTTTAATTTGTTAGTACTTAAATTGGAATAAACGACTCAGGCAAGAAAGTTAATGA
ATTGGCAAACTGAAGAAGGCTATAACAAATTCATTTATTTTAAGCTTCTAAAACTGGCCTATACAAGGTTGTCCCTAAGC
ACCAACTGCTGGCGAAATTCACCATACGAAAAAGTGGCAGTCACTGGCTGGACTTTTGGCCAGTGATTGCTTGGTAAAGC
ACAATGGGCACTTCTGAGGTTACACACTAACAATCATTCGCCACCTTTCTGCCATCTCACTTGAAATTTCAGCCAGCAGG
GTAATTTCTTAGCTACAACTCTACTTACAATGATTGTACATTGTATTCACGACTCACCTGTGTTCTTGCAATGCTCTCTT
GAATCACTGACCTGGAAAGTGAACCACTCACAGGGAAACAAGAAAAAAAGGAACCAAGTACATTTGCAGATCCACAAGCA
ATCAGTTCCTGCAAACAAACAAAAATTTACTTAATATGTATCAGTGATCTGTTAACTAAAACATTTCAAGATTACCCCAA
ATCAGATTAAGTCAGATGAACCAACAAAYACCATTTTGTATCTACCAACCTGATTGGAATCAATTACATAATTATTCCTC
TTGGCAAATGTTTTGGCCAAAGATATGTTTGTAGCAAATATAACAATGGAGATCACTATGGCATCTGTCACGATATTGCC
CATTAGCATGAAAGACGGAGCAGATGGTACTCGTAACCTGGGTGAGTAATTAAACATTTTGGTGAAATCATTTTTGGTAT
CATTAATTTGCAAAATAATTCAAAATGTATCAAATGTATCAAAACATATTAGTGAGAATGAAAGTATATTATATCAATTG
ATCAGTGTCAAACATGTCTATAATCCAAGATATTTTTCTCATTTATTTGTTTGTTTCAGGTATACATACAATGTACAAAG
TAAAATGAACTATGACAAAGATAGAGATGATAAGACTGTAAGCAAGACAAAACACATTAATTCAATTCTACAGAGTGTAG
GGTACAAGTACATGTATGTGCAAGGGAAGTTATTGTAAAGAGTTAGTTTATAATTAACACAGTTACAATTTACACTTCAA
TCATATGACTGTATTTCAGTGGCTCTTACCCCTTAGGAATGTCATGCAGAACTGAAACTTGAAATTTTTCATTTAATCCA
ACAAAATAGGACACCACTGTCCCGAGAACAACCTACAACCAACAACATAATTACACATCAGATGAATCAATCTTTCAGGG
TTTCTGACAATACAGAAAAAGCTTTCAGTGAATACTGCAGAGTTCCAGAACTTCATCCAAGATGAGAAAAACAGCTTCAG
TAGATAAATTTATATACAGTTCTTGAATTAGGGACAGTTGTACYACTGATATGTAAATTCACGTATAGGGAAGCTACTAA
CAGCAAAGTCCAAGTTTATTTCACACTATTCAAATTAATCTCGGCTGGGGGAATTATGTGCTGATGCAAAAGCACATAAT
TTTAGCTCTAATAATATACATGAAAAAATTTCTCAGTTCTGATTGGTTAAGATGAATGCAGTTTTCAGTTAATTCAGTGC
AGAAGAGAGTTAATTGAGTGCAGAAGAGAGTTAATTCAGTGCAAAGAAAGAAACAAACCAGACATTCTGATTGGACAGAA
TGATTGGTCAATGTCAATGTCAAGGTCAATGTCACTTGCAGGATTTGAATAAATTATTTTTGCACGTGATGCACGCGCTT
TTCTTCTGCGTAGTTATGAGAAGCTACCTTGTATTTTTTCATGTACAGTATATTTTTAATAGATAATCACATGATTTTCT
CATGCAATTTGGAATAAATAAGCACTTGTAAATTTTTNNNNNNNNNNNNNNNNNNNNNNNNNNNNNNNNNNNNNNNNNNN

NNNNNNNNNNNNNNNNNNNNNNNNNNNNNNNNNNNNNNNNNNNNNNNNNNNNNNNNNNNNNNNNNNNNNNNNNNNNNNNN
NNNNNNNNNNNNNNNNNNNNNNNNNNNNNNNNNNNNNNNNNNNNNNNNNNNNNNNNNNNNNNNNNNNNNNNNNNNNNNNN
NNNNNNNNNNNNNNNNNNNNNNNNNNNNNNNNNNNNNNNNNNNNNNNNNNNNNNNNNNNNNNNNNNNNNNNNNNNNNNNN
NNNNNNNNNNNNNNNNNNNNNNNNNNNNNNNNNNNNNNNNNNNNNNNNNNNNNNNNNNNNNNNNNNNNNNNNNNNNNNNN
NNNNNNNNNNNNNNNNNNNNNNNNNNNNNNNNNNNNNNNNNNNNNNNNNNNNNNNNNNNNNNNNNNNNNNNNNNNNNNNN
NNNNNNNNNNNNNNNNNNNNNNNNNNNNNNNNNNNNNNNNNNNNNNNNNNNNNNNNNNNNNNNNNNNNNNNNNNNNNNNN
NNNNNNNNNNNNNNNNNNNNNNNNNNNNNNNNNNNNNNNNNNNNNNNNNNNNNNNNNNNNNNNNNNNNNNNNNNNNNNNN
NNNNNNNNNNNNNNNNNNNNNNNNNNNNNNNNNNNNNNNNNNNNNNNNNNNNNNNNNNNNNNNNNNNNNNNNNNNNNNNN
NNNNNNNNNNNNNNNNNNNNNNNNNNNNNNNNNNNNNNNNNNNNNNNNNNNNNNNNNNNNNNNNNNNNNNNNNNNNNNNN
NNNNNNNNNNNNNNNNNNNNNNNNNNNNNNNNNNNNNNNNNNNNNNNNNNNNNNNNNNNNNNNNNNNNNNNNNNNNNNNN
NNNNNNNNNNNNNNNNNNNNNNNNNNNNNNNNNNNNNNNNNNNNNNNNNNNNNNNNNNNNNNNNNNNNNNNNNNNNNNNN
NNNNNNNNNNNNNNNNNNNNNNNNNNNNNNNNNNNNNNNNNNNNNNNNNNNNNNNNNNNNNNNNNNNNNNNNNNNNNNNN
NNNNNNNNNNNNNNNNNNNNNNNNNNNNNNNNNNNNNNNNNNNNNNNNNNNNNNNNNNNNNNNNNNNNNNNNNNNNNNNN
NNNNNNNNNNNNNNNNNNNNNNNNNNNNNNNNNNNNNNNNNNNNNNNNNNNNNNNNNNNNNNNNNNNNNNNNNNNNNNNN
NNNNNNNNNNNNNNNNNNNNNNNNNNNNNNNNNNNNNNNNNNNNNNNNNNNNNNNNNNNNNNNNNNNNNNNNNNNNNNNN
NNNNNNNNNNNNNNNNNNNNNNNNNNNNNNNNNNNNNNNNNNNNNNNNNNNNNNNNNNNNNNNNNNNNNNNNNNNNNNNN
NNNNNNNNNNNNNNNNNNNNNNATTATTAAAAACTAGATCATTGGATAATGCAATTCAAGACTTTTTATTGGCTTAGCCA
TTATGGTATATCAGCCAATATACCATAATCTACAAATATGGCAAGCGTACGTGTCAGCTTAAAATTAAAAGGGAGCTAAA
AATTTAATAAAATTTAATAAAATTTAATAACTTAATTTAACGTTTTTAATAAAACAATTATTCCACTCGCTCTTGTTGGA
TATGAGATGACTATAGCCAACTCGGCACTACGCGCCTCGTTGGCTATCTATCATCTCATATCCAACGCGCGCTCGTGGAA
TAATTGTTAAATAACTAGGTTTTCTTAACATGGACTTACAGATAAATTTAAAATAATTATTAGTATGTAAAATTCAATAC
TTTTAATATTGCACAACTTGCATAATTTGCTAACTTTGGTGCATAATCACAGAAAAAATGGTGCACAATTTCTCATAAAA
TAGTGCATAATTCCCCCAGCCCTGATTAATACAGTATAAGATGACTTATATAACTAGGTGAAGAAGATAATGAGAAGTAG
ATATCATGCAGGTTTGTATATGTGTGCACAGCGACCAAAGTGGCCGTAGCTTTCATGTTGGTCACTGTCCCATTTAAGTA
ATAGAGAGGTCCAAGATGCAAGTGTGAGGTAATAATAAATTAGGGTATTAAGAGAATAGTTCTTAATATAAATATATAGA
ATGAATACTAGACTTATTGAAACAGCACGATAATAATTATACCACATTTAGAATTTATAATAAAAAAGAAAAATAGGAGA
AAAAAAAGAAAAGAAAAAAGAAAAGAAAAGAAAAGAAAAAGAAGAAAAGAAGGAGAGTAAAAATAATCTAATTATATATA
CTGCTGCTGCATTGTTCAAAGAAGACGAGAAGCTAAAGAGAATAGGGTATGAGCTGTTTCCGTTATCATTGGCTTTATAA
TAGAAAGGTTTTATAATTTCTTTTAAAGGCAAAGCTATTAAGACGGTTTATTCGTCCTTATCCGAGAAGACTAGTCTAAG
AATATCTATCCATTTCTTGTTATAGAAAGGTAGCAAATTCTCCTCAGTTATTTTAAGACCATGAGTGTTGGTCTGACCGG
GCCTTGAACCCTCGACCTCCTGCACAAAACAATCCGGCGCACTAACCAATTGAGCTATATTCTGCGCAATTTTTTTTTC
AGGAGAATTTGGTGTCAGATCAAGATAACAAAATCTACTTGATAATTTTGTATACACTCATTACCTGTTTACTGGATAAT
GCATGGATACTATTGGGAGAAGATACACTCACTCACCACTATTAATTCAGCTGGTATAGGAAATTTCATTTTGTGCTTCA
GCTTCTCACTGATGTACTTCAGAGCAATCAGGATCAGGATACAGACAACCCCAGTGATAACTGATCCTGGTACAGAAAGA
GTAATATTCTTGAACATAAATAAATAGGTCTGAAACAAAAAAAAAATAAGATATCAGAAAAACAAGCAAACAAAAAATCA
AACTAGTAAATCCTACAGGTGCAGCAATCAATGATCTTGTTACACATTCCCTTCCACTGAATATTTTCACATTCAAATGA
AAAAGTAATGAGGGTTATCACAGACTTATTTTGTCAAGTAATTGGGTGATAAGAATAGCAAAATAAGCCTCAAGTTTCCA
AAATTACATGTATTAAAGTCATTAAGGAAAGCAAAGAAAAATATATTCAAACAAAACGAACAAGAGAGTTAGTATTAAAA
GGGAGTCTTCTTTTGAGCACTTTTCTACCATGTTGCTCCTGCTCAGTTCCTTTCAAGGGCATAGTAATGTTATAAACTAT
TGTTACCAGGTCCTTGCACAGATAAAACATTACCTACTACAAACATAAATGTGATGAAGATGACTCCTCAGTGCTAGTCA
GCTGAATGGATATCTTAACCAACCTTACAGTTTCTCAGTTACATTGTAAATTTATGTATTAATTACTTCACTGAACAATA
ATGAAATCTCTATGTTGGTTCACACCTCCTCCCTCCAAAGGTGAAAAATATATTTAACAAATGCTCAAGATGAATACAAA
CCTTAACTACAGCGAATGCTCCAGAATATCGTGGAACCTCTAATCCCAGGATGTGTTTCACCTGACTGGTGAAAACCAGA
ATTGCTGCACCAGTTGTGAAACCGCTAATGATAGGATCAGATAAATAAACTGCCACAAAACCAAGCTTGAATAGACCCAT
TAAAAGCTGCAAAGAAAATCAATCAAATAAAACTGGACTTGCCAAATGTTAATTCGACAACTTATTGACTTAGGTCCTAG
AGATTTTGCACAGCAAAGCTATAATACACTTTGCCAGAAGTGATGAGGAATGCTAAATCTTAAAATAATTTCACACAAAC
ATTGAAACCAATATCATCATTTAGATCTGAAATAACTTGACTTTGAATAATTTTAAGGTAATTTATACTTTTTCTACATT
TTTATTGTTCTGTCTTTAATTGTACTTTTATTGTATTTTGCTTGAAGTGGTGTATCTGGGACTAGTTGTTTAATAAAAAA
AAGTGACCTGTAATTAAAGACTTAAGAGAACATAAAAAGAACATCAAACTAATTTATTAGTAAATAAACCCCTTTAGGTG
GCTGGTATGTCAGCTAATAATGTCCATGCTTGAGAGATTGTCCAAAAAATAAATAATTGGCCAAGAAGCAAAGCTTCACG
GGAAATGTAGATTATTGAGGACAATCCCTCAGCCAAGGAAATTAATAGCAGACATACCAGCCAGCCAGGAAGGAGTTTAT
TTCCTTCATAACTCTTTCATTCAATTTCATTCTATGCAAAAGTGTCCTGTGATTTTACACCACATGACTTAAACAAGCCA
ATAAAATCACACAAAAGTTTATAGGTGGGTTAAAACTATCACTAACAAACAAATCAAAATAAACCATCTGTTCAGAGGGA
TCCCACCTGTAGAAGACCAACAAGCATTGAAAGTGCCACGGCAACTTCTAACTTCTTTTCATCCAGCAAGTCCTGTTCAT
CCAGCGAACTGCCATCAGACTCATGCTTATAAATTGAATTCTGTAATACTACACCAGTTGAGCCACCAGGGGGCTGGTTG
GTAGTAGCTGGTGCTGTTGGCGTGGGTGACAAAGAATGCTCTACTTCTCTGTCAACAACGTGACCAACCATTAGACAAAC
CACAGCAAATGTTCCTATAATTAAATTATCATGATTTAGATTCTAATTTCAAAACTATCAACATTATTTTGTAAAATTAT
ATTTAATTAATTAAAGTTCCTAATAAGCATTTGAGAAAAGAAAAGGTTTTGATAAGTTTTTAAAATTGGGAATCTTTTAC
ATTCTTCATTTGGATCTATACACCTGTTATACAGAACCTTTCAATCACAATGAATATTTGATAACAGAAAATTGACCACT
AGGATACATATCAAACCTTATGTCTTACATGCTCATTTAGTGTTTACAGACTTTGCACAGAGATGTCAGTTTTTGTTCTT

TCGAAATTTTGAAATCATTTAAATTAACACTTCTTGTGCTTACAATTAGTATTTTACCTAAAGATATATGCCTGGATGTC
CCCATTGCCATATAAACAAGAATAGGAACAAAAGCTGTGTACAGTCCTGTAACAGCTGGAAGTGATGCTAGCAATGCATA
TGCCAGACCTGAAACATGACAACAAGATCAGACAGATGCAATATTTATCATATTCATTTGGGATTTTTTGTGGGCATGAC
TATTCTGTAACATGCTGCTTTTTTAGAAGGAGAGAAATGTTTCAGCTAATTACTTCTGAAAGTTCAAAGGTGAATACAAG
TTCAGTAAATCAYACAAATCAGCTATTGATTGTTTAACTCCACAATAAACAAAGAGAGTTGAAATGTTTCCACTATCTGG
TTGTTTTGGAGAGATAGTATGAAGATTTTTTAGTAGAAGAAAATTCAAAGTCAAGGCATGTCATCTGAAAATGTAATCAG
GGAAAAGTGAAACTCTCATTTCTTAATGCACTTTCTAATTAACCTTTTGATTCCTGGAAATTATCAAGATGTAGCTTTAA
ATCCTTACAAAATCYATACATTATTCAGCAAAGGAGGTAATGAGAATATTCAAATTTATCAGGTATAACTTGTTATCTTG
ATCTAGGGCTCGACACTAACTTTTCAATTTACTAGCCAAGTGGCTAGTGACAACTTAGATATCACGACAATTTCAGTTTG
TTTGCTAGCCAAACTGACAGGCGGAAATAAAAAACCATTCACCAAAACTGAATTTCTACTAGTCTGTGGCTAGTTGGCTA
CTGTTATAAGTGTCAAGCCCTGTTGATCTCACATGAAATTTTGCCAACTAATTCACAAGGAAATATGTAGCAGCAAGAGG
GGAGAATTAGCAATCAGATCATGGGAGTTAGAGGGTAAACTAAGGTTCCAASTCACAATTGCCACTAAAAGGAGTGTCTG
TTGAAGGCCGAATTAATTTTATTTAAAGAGATTACTTGATAGAATTAATTTGCAACTTGCTTTTGTTAGTGTCTACTGAA
TACATATTCATTATCAACCAAGCCTGTTCGGTCAAGATGGCTGGATATTGGTCGAGTTTCTTTTTTTTGCATTTCTATGG
ACTGAGACGAAAGTCTCAAAAAAAAAAAAAAAACAAGGCTAATATCCAGCCATCTTGACCAAACAAGCTTGGTTAATAAA
GGAATTATTATATTGTAAAAAAGATTTGGCTATTTTAAGAATTAAGAAATGACTTGTTTACTTTGAGAGCCAGGAAAGAA
AGCCARCTGTGTTTGTAGCACAACAAACTCACAAGTCCTTTCTGTTTTCTTTGCTCAGACTATCTTCTGCTGCTTTTTGC
ATTTCCATCGCCAAAATTGTTCAAGAATTACAATCGTTGTTCTAATTTTATTTTTTTCATGCGCAAGATCAATGCAGGCA
ATCCCAAGCAGGCAAGAAAGGCCCATCTTGCCTGCTTGAGTAGCCAATCAGAACACAGGATTTGCTTCATCTTATCAATT
GGGCACTGGGAGTGATGTTAATATAGGGTGTTACATGTATGGGGTTGGCCTAGTACTANNNNNNNNNNNNNNNNNNNNNN
NNNNNNNNNNNNNNNNNNNNNNNNNNNNNNNNNNNNNNNNNNNNNNNNNNNNNNNNNNNNNNNNNNNNNNNNNNNNNNNN
NNNNNNNNNNNNNNNNNNNNNNNNNNNNNNNNNNNNNNNNNNNNNNNNNNNNNNNNNNNNNNNNNNNNNNNNNNNNNNNN
NNNNNNNNNNNNNNNNNNNNNNNNNNNNNNNNNNNNNNNNNNNNNNNNNNNNNNNNNNNNNNNNNNNNNNNNNNNNNNNN
NNNNNNNNNNNNNNNNNNNNNNNNNNNNNNNNNNNNNNNNNNNNNNNNNNNNNNNNNNNNNNNNNNNNNNNNNNNNNNNN
NNNNNNNNNNNNNNNNNNNNNNNNNNNNNNNNNNNNNNNNNNNNNNNNNNNNNNNNNNNNNNNNNNNNNNNNNNNNNNNN
NNNNNNNNNNNNNNNNNNNNNNNNNNNNNGTGCTCTACCAATTGAGCTAACAAGCCAACTGGGAGCAGGCCGTTAAGTTG
ATTCGTAATAAACCTGTGATGATAAATCAGATACAATGAAATATATGAAGTTCATATATTTTGAACTGCGGTTGTAGATA
CGTATGATGCACGATCCTCGCAGTAGAGATCGTTATATGTATGTCATCCCCAATAGTCACAACATGGGGGGAAGAGTGCA
TTCATTCTTTCGCAGGTGCAGGTAGAGACAGTAGGRGTAGCAGTTGTTGCGATTAATAGTCTCCTGACCAAAAATATAAC
AGAGATTTCCATATAATCCCATAATTCTAGAACAAAGCCGTAGTCCCGAGGGTAATCTATTGYTTTCGCCGTTGTTAKAT
ATCTTTATCCAAGAACTGTACGAARCTGTGTGGAAATCTCGCCAAAATTTACCAATTATTTGTTGAAAATTATTACTAAT
TGGCTACTTTTTTAAGGATATTTAATGCTGTTTGCAGWTTACTCTYCTTACCTTGAGGAATATGCATGATCGCCACGGTT
ATCCCTCCTGCTATATCGTGCGCTAAGTCACGCCGAACATTATASGCTGGTAGCCATTCCACTATGGGAAACCATGATTG
TAAGATTTCTTTACACGTACATCTAGATGGCAGGAAATTTCGCCTTAATGTTTTTGCAAAGGAAGAGGGTTCAAATTCAG
AGCTCGCTGTGTATTCCTCTTGATTGATAAACGACTCAGTGAATTCTTGTTCATTGTAAACATGTCGAGACAAATTGATT
CTGTATCGTCTTCGGCGTTGACGAGTTATCAACGGCGGACATTCCGATTCTCCTAAATCCTTCATAGTCCTTGATGTGTG
ACTTTCAGTTSACTTTTCATAAATTTTAAAGATTTTGTTTCAAAACAACTCGCTGATGATCGACAGGTTAAACAGCTGAC
GAAATATCGCGAGCCGGTGAGGTAAGCTCTCGCATCCTGTTTTCTCTGCACGAATATCCTTGCACGATTTCACTTGTTCC
CACAGCATTTTTACGTTCCACACATTTGTCTTCTGTATACTTAAGGCGAGTGTTCCTAACTGGTTAGTTTACAGCATCTT
CGAGCCAAAGAAGTCGAAGGAACATATTAGCTGATCATGAAACTGAAGGAAACGTAAA
>scaffold_7 1047191 1058109
GGGCACCCCCCCCCCTCTTATTTTGGGTAAAAAATTAAACGCAGAGGGAAGAAAAGCCGGCAGCTAAACACACACAAACG
AAAACACGCCCTCTCCCCCCCCCTCCAGCTCAAGCTCTGGATCCGCCACTGATAGCCCTTTATGTACTTTTCAAATTAAG
TACAAATGATTATTTTATCGAAAAATTAGAATATTTTGGAACACATTCACTCCATGAATCGTAATATTATATATATGTAT
GTATGTATGTGAATTTCGCTTTCTACAAAATCCCTCTGACCTTAAAAAAAACCAAAATTATTCGTTAAGTAATGTGGAAA
TTGGGAGGCTAAGAGCGCGATTCYCTCCCATTCCGAAATATCCACCAAAAATTTGAATTTTTCAGTGTGTAAACCTTAAT
TATTTTTTTGGGAGACACTCATTTAGAAAGAAAGAAAAAAAACTCAGCCAGGATCAGCATTAAAAACATGTCTCCTTGTC
TACGTCTTCTCCCCAAATCTGCATGGACAACAAAATAAGGACATATTTATCTCTGTATGTAACCATGATGACTGTTTACC
CTTATTGCAATGTGTAAAGTAACTATGCATAACAATGAGTTAATTGTGTACTGTATGTGTCTTAGAATGCACTCTCCCGC
ATATTTCGCCACAATATGCCTCAGTGTGTTGTCATCAATCAAAAACAATGATCCATATTAACAATGAGCYATATTAATAC
CTGTATTTTAACTGTCAAAAAGACCGGCAAAACTGGCCAAATATARGAAAACACATTGCAAAAACTCTGGAAGATCAGAA
CGAAAGGTGACAGTTTCCAAAGTCAGGAACACCACAGTAATCATGGTAACACTTCTTGTATCTTGCTCTTTGTTGTCAAG
GCAAAGGCATGCACATCAGTTTTTGTTTAGCCGTCAGGTGATTGTGAACTTACCGCGTGGTCACGTGATCGTATGGCAGA
TACAACAGCGTCATGAATGGATGGAAACATCGCTTGTTGTGGAATGGCCTGTGTGGAGGCCTCCCCAGAGGAGCTTGTGA
ACTTCTCAATGAGGAGTGCTGTGGAGAGACGAAATCTCTTAGGCCAGAGAGTTGGACATAGCCCTTAATAGGTTTAAATA
TTTTTATCGTGAATTCCACCCAAACCCTGATTTAAAACTTAAAAAAGGGAATACTCACAAGAACAACCAGCCAGGTATAT
GTGAATTCCAGCTTTCTTGAACTCTGATAGAAGCTAGAGGATAAAGTAAGATTTCTCGAATAAGTGTTAAAAAACTGATG
AATACAGGTAGGGTCAKAAGTAGTCACTGTTGTCATAGCGATCATGGCCGGCGTAAAAAGTGACGTTTTATTGAGGCAGT
TTTTTATGCGCATATCCAAGTAGTTATTGAAGGGTATTCTGACACCGGTGGTCGTCCTTCGCTGGACGGAAAAGATTTTA
TTTATTTTTCATCGTGGAAGGAGATTTTTGTTGAAGATATCCGTGGGGGAGAAGAGTTGAATGAGTCTGTGGGCTAATTT

TTCACCGCCTTGCCCTCCGGTGGGGTTTACAGAAAATGTAATCGTACAATTAACAGGAGAAAAAGGAAAGTGCAGCCGAC
GCACTAAGGAAGAGTATGAAAACAATTGACATTTGCTATGACACGTCTTTGCGTCTGCAAAAGTTGCGAACGAGTCTGCT
ATCGTCGTACTGGGAGGTGATTAAGAATAGTATGAAAACAATTGACACGTGCTATAAACACGTCTTTGCGTCTGCAAAAG
TTGCGAACGAGTCTGCTATCGTCGTACTGGGAGGTGATTAAGAATAGTATGAAAACAATTGACACGTGCTATAAACACGT
CTTTGCGTCTGCAAAAGTAGCCAACGGGTCTGCTGTTGCCATACTGGGAGGTGATTAGCGTTAATCACCGAGTTTTATTT
GACTGACACTTATTCACCTGATAAGGTATTGATGCATTTTTTGATTGAGTCACTTTAGGGCATTTTTCGMTTGAGCGGGG
TAAATCAAAACCCGAAGTAAACAGGCCAATAAGAACAAAGATGAATCTTATTGTCAACAAGTAACAGTTGGCGAAATCGC
CACTAGTTCTTGTTTTGCACCTGACAGGCCTCGTTCCCAGGGTCTCTCATCTTTCCGCTCCAGGGGGCGRGAATATGAGA
GAGCCTGGGAACGAAGTTGTGCATCTGATTGGATGAGAGGGTGACACGAAGCATTCCCTCCCAGGGTACTTTTAATTCCC
TTTTGAAGATTGCTCAGTTAGCGAAGTAAAATTATAGCCAATCAAAACCTATATTAGGCGCCAGTCATTATTTTATTGCC
GGTGTGTGGAGGATTTTAGGGGATCACATAGTTTTTAGAGGGAACAGATGGGGGACCAGTCGTTGCCAACAGAGCGTTAA
CGGGAGAGGGAAATATAGAAATGACGTGCGAATTGTAAGAGTACCGCAAAGACTTATGGGGTAAACTTAAATCTATGCTA
AGTACTTACGGATGGAAGAACATGAAGACCAACAGAATCGATGTAGGTAAAGCTGCTGCAATCAATGACGACAGTGTGAA
TACGGTTTTTCGGCTCATCAACTTTAACAGCAGTTGTTGTTGAAGTGCCTCCTTTTTCCTGAGGTTTCGGTTTCTATTTA
GGAATTGGACAACGATTACATTTTTCAGCAGTCGAGACGATTACGGGCATGTTACTACTTCAAACCAAATTGCGAATTAA
TTTTCCTGTCATGACCTCATGAGAAGAAGAACCTCGAGAGAAATTAACGGTTTTGGTGCTAAACGTAATCACCCTAGAGC
TATGCCGGCATCACAGARGAGCAAACAGAAGTTTAAGAAAAGTTTTGTCAACATTCTTGATCTCAGAGATGTGGCATCTA
TACTGTACCATTGGCGGTGACACTGTTATTGTTCCAGTTGTTGAGCTCTAAAGATCTTACCCTGCGAGCAGAGCCTTTTC
TTCTTTTCTTCTTCTTATCTTCTTTTAAACCCTCCAAGGGAAAAGGCTCTGCCGACATCCTGTTGTCGCTTTGAAATGCC
GAAAGCCCAAAACTCCGAGCTACTCAGCCGGTTTTGCCTCCAGTCAAACCGGTTTTGGTGCGCACGCCTTTGTTTTTCAT
GTACACAGATAACCAAATCTGCGCCCACAATAGTTCAAGGGGGCCTRGAAAAGGATCAGTTTCTAGGCAACCGCCGYGCA
TGTTCAAACGAGCAACAACAGGAKGTMGGCAGAGCCTTTTTCGCTTTTCGATTCGAGGACGGGTTAAAAAGGCTTTKCTC
GCAGGGTATAAAGATCTTAATGCTATTCTAATGAAACCACCAAWGACAAGAGCATTGATGTCTATCGCRGCKCCATTATT
ATTCTGGATCAATTTCAGTATCTGAGCAGYAGCGCACCTACCCTTCCCTAGGCKCCGTTACCAGCCGTTGTTCGGGAACC
TGAGSCCGCGYTACTCCGGAAAAGACCGGACCTGAGAGCGGCGGAGATCGAGTAACCCTTCGCCTAAGCCAGCATTAACC
CGAACTTGTTATTAGTTAACTGTTGTCGGGTCAGGGGAGGGGTAAGTGCACAGTTTTTCAGACACTGGCTGCCATTGATC
CATTGTTCCCCACGAACAAGGAGGAGGAACGCATCCATACTTGAACAATCGATATTACGCCATTTTCGGCTTACTGCTGT
GAACACAATGTAAGAGCCAAGTTACTAATTCTAACTTACAGAACCAGGGTTCCCTCTGTTACTGTTTCCTTTGTCTTCAT
TCCGGAGAAGATCATTGTTTTCCTCGTCGTTTTTCTGTTTGACCTCTTCTCCCGCTTTCTGTTCAGCACTGGGATCTAAT
CCAGTGGTACTAACCAGCGCGCTTCTGAAGCGCTCCGCGTTACCAAAGTAAAGTGGGGACTCAAGTCTCAGTACTTTCAC
TCCAGGGACCTCGAAGGCCTTTAAAAAATGTCACATGGAAACGTTATTGTTCTGTGACGCGATCGAAGTCGGATATAACA
TTTCACACAAGTTTTGGCTTATCAACTTGAATTTAACATTGATAACTCATATCCTTGGAACGCGAACTTACGACAAATGA
ATAATAATAATAGATAATTTRGCAATGGACATTCCACTGGGTGGCTCTTCTTGTCCACTGTTTCCAGATCGAATTGGAAT
TTGGAATGCTGGTTTTTGCGGAGGGAGGAAAACCGGAGCAAGGACGAGAACCAACAACAAACTCAACCCACATGTAACGC
CAGGTCCGGGAATCAAACCCGGGCCACAGTGGTGAGAAGCGAGTGCTCTCACCACTGCGCCATCCCTGCTCCCCTACAGA
AAGCAATCAGGAGAACAAAATAATTATGATAAATTACTTTCACAATTGAACAATAAATGTACCGTGGGACATTGTTTGAC
GTCACGATATAACTCAGTTGCTGTTACTCTGCCAAGGATGGTGTAGCTGGACCTAAAATGAAGATGTTTAGGAAAAAAAC
AGTTCAAATCAGAGGGGGGATGTAGAGTCTGAAGGAATGAGGGACAAAGGGTCGAAAATTGCCACAGGGTCAATGTATTT
CGAAGTACTTAAAGTTTCTGGATCAAAACCCACAACTGGTTTAGGATATCCCCAAGGGTATATTTGTTTGTACTCTGCAG
AGTAATGTTCTTAAACTTAGATCTRCTGCCACTGATTTATAAATAGAATATAAAATGTTCTAGATTTCTAAGCATATCTA
TACAGGAATTCATGACTAATAGCAGGCTTAGAGCTGTCAATGAAGCTGCGTTGCAGACCAACAAATGACCACTACATATA
CTTAGTGGTTTGGCCAGCCGAAATATAGTGCTCCGTCTTAAAACTTGTCTACGTTGTATCGGTCTTGCCTCTATATTTAC
AAAGGTAAAGTATTTTTTACCTGGAAAGGGTGAGAACGACTGCAAATAACGCGCAGATCACTCCTACACCCAATCCTTTA
TCAACTCCCAGCAGAATGACACCAAAGCAAGAGAAAAACCATACAAACTGTAAGAGTTGWAAACAAAACAAAATATAAAT
CTTTTATACCACCTTTTGAAAAATCTTAAATAAATTCACATGCAAAAAGTGTTTCACTATTTGTGGATTGATTCGGATG
AATAATTGAATTGTAGCTTATAGCTCAAATATCAATGTCCGAACGGTTTTTTTTTGTTTGTTTTTTTTTTGTTTTTTTGC
AGTCTTTGGGCCCAACTTCACTTAGAAAAGATAGTTTTATGTCGTATTTTGACCTCATRATACGGCGGCTCAGAAAAGCG
ATATCAGCGGTTATTTTAGCTCACTATTACGTCACTGATGGGGAAGGAAGATAATAATCAGAAATTCGGTATGGTGATGG
AGTTATCGAAGTTGCGTGGCTTGAAATGGCGATCAAATTCGTTAATCCTTTGAATGTAAAAATGACTAGCATCTAATTTC
TCTTTACAAAAACGCCCCTAAATCACACATCATGGTCAGGAGAAGAAAGGAAATGATCACCAACTAAAGATGCTCATGAT
TGTTTCACAAATTCTCCTTGTCAGCACCTTAGGAAATGCACAGAGGACAGTATGGAGAATATGCATACTGATGTTAGGGT
GTAAAGGATTAATAGAGAACTGTCTCTACTTATTTCACTGTTTCTTACCGCATCAGGTTTGTAAATTCCCCACAAAGCCC
GTAGCCTTGAAAATTGTTTGAGTAGGCCAATGAGATTGGCGATCACAACTGCAGCTAGGATAGCCTGGATTAAAGCAAAC
ATAACACAATGTCTTCAAAAGTAATAATAACAAAGAGTGACTTTGTCCTGTCTAAGGCCTGACAAAGCAAACGCTGGGGT
CAGAACAATAATTTTTTTAAATGTATAATTCACCTTTGGGAGATGGAAGAAAAGTGGAGCAATGAACAGCAGAACCATAA
GGACCAAGGCGACCACAACCACGCTGCACAGCTGTGARATAATTAAACAAAAGAAATTTATCAATCCGTGGAGAATCCTT
TTACACCTGCGCAGAACAYTTCTTTTCAGGATAGCTCTAAACCGGTACAATATGAAGAAAAACTGACTCTATACTTCTTA
AAACATTTAGTATGATAAACGGAATGACACAAGTTTGCAACACGACCGTTGTTACACTTATCGAGAGGTGGCCATTCAAG
TTCAAGAAATAATGTGTAAAATTTGGCGGAGTTTGCTTACCTGAGTGTTTGCAAGGTTTTCCTGTACCGCTGTACGCGCT
AAAGCGTTGCAGATCGGGAAACACGAAAAAAAAAGAGCCAGCRAGGTTTCCTGCTCCATATGCAATCAGCTCCTGAAATGA

GAAGAGTGGAGMAAAAACAGCAATATGATTACATCGATCTCTGTTTCGCTTAACAAGAGCTTCCCAGCCCAGCTGGATTG
ATAAGGATCAACGCACACTAAACTGGATGTTCCCTTTTTCCGGTCGCCACCCGCGTCTCAGAGAAGTCAGTTCCATACCG
GAAGTTGAAATGCAGTTATGTTTAAGGACGCTTCTACAGAAAAAATGAACTGAATATTTTACGCTGCAACTTTTTTTGCT
AACGTTTACCTCGTTACTGYGGTCGAGGTAAGGCATTCCATCAACAGAATAAAGCCACGACAAATTAGTTGTTGAGTAGG
ATAAGGATTATGTTAGCTTACAAGTTACAGGTATCATTCCAACGTAATCTAGCTGTTTCAGCAGCATGGAATGATAAGGA
ATSTTTGTATTCCCCCCCAGTTAAAATCAATTGTTCTGTAGACTATAGTTAATCATCTTAAGTTGCAAGTCCATCATGGG
GGAAGGGGGGAGTACCTCCCTATTTCGTCACGTTTCACTGACAGTTTACCAGCACCCATTTACACTCCTGGCAAGGCGCG
TGGGAGGCAATGTGAAAAACAAGTATCTTCCCAAGAACCCAACACCGTGCACGAACCACTCCGTCGAACCACGACGCCAC
TACGTATCCCACGAATTCCTCTTCGGTTTTAATTGTACTTTATGTATAAAGCTTGATAAACAAACCTGGTTCGGGTCGAC
TGAGTAACCACGCTTCTTAGCAAACATCTTGGAAATTGAAATGTTTGTGGCAAATATCACCACTGCTATGACAAAGGCGT
CTGAGAAAATGTTCGAAATCCGGGATAAAGATGGTACACTCAAAGGAGGAAGCCTGAAACCGAATYAAACGACATCGTCA
ACTTCGCTACAAAGAGAAAACACAGAGAGAAGAAAGACACTAAGAGATAATTAAACAAAACTGGTGAGCTAGGGAGCTGA
TGAAGAGAAAATTTCTTCTTGGCAATTCGATATAAACTTGGGACAAACGTTTGCATAAAGAAACGTGGTTTATTAAAAGA
CAATTATGAACTTTTCCTYGCGGACACAGAGCTACTAAAATCTAAATAAATGATCTCAGACTTTGAGTTAACGCTATAGT
AAGTTACAACCTAAATGTGTGTGTGTGTCACACAAAGGAAACTGCTCCTGCTATATGTCCACAGACAAATAGACGCRGTT
ACGTGTGTCTGGCGTACCGTTAGGCATATTACATACCCCTTTGGTATATCTCCTATGATTTGTGTACCATAATTTAAACT
GATTTTCGCACCATATGATATAAGAGTTCCGACAACAACCTGGTCAGATAGAAAAGGAGACAAACGTAAGCACAAGCCGA
CCAACAACACAACAGTATTATGAAGGTAAACGTAGGAAAGCGGAAACGCAGAAAGCGAAAATCAGTCTAATTGTTTTAGT
TCATAAACAATGCACGTGCGAGGTAGTTTTATTATAATACTCCCCCTTCCACACCCCTGCGAGTCTCTTTATTCAAGTCG
TTTCCTAAAACTACGAGAGTCTTCACGAACAATCGTATTTCGTGAAATATCCGCAAAATTTATGAAATTCACTAACTCGT
TACTCTTGAGCACCCTTGTTCCAGAAGTACTTTCCTGTTAGTCAAGCCTTCGATCAAAGGTAAAACCGAACAAGAAAAAA
ACAGAACTTCTAGGGTTTCTCTGATTGTTTTAACTGTTTGGTTGCCAAATTAATAAGTAAAGAATGGCAGAAATGATTAA
ATTTCGAAAAAAGAGAGAGGAAAATACCTTCTAGGTCCCAGTTTTCTCGTTCTGAGTAAAACTAGCTACGTTGTTTCGAG
CCTCTCGTGAGGAAATCTTCATAAAAAGGATGGAAAATGGCCATGACATTGTTTCACAGGCACGGCTTCTATCTYACAAA
GTAACCGGATCAAATAAATGTTCTTACCACAAGAAGTTCAGCAGGGATGGGAACTTTCATTTTCTGCTTGTATCTCTCGT
TCACTTGTTTCAATCCAATTAACACAATAAGACAGAGCACGCCCGTGATAATGGCTCCAACGTTCGCTGAGGGAATGTTT
TTCAGCATATGAATAAAGATCTGAAAGCAGTTGTCATTATCATTAGTTTGAATCATTGAGGAAGTTAGATCTAACACGAC
GTCTCAATTGGTTGGGCATCTGTGTGTCAAGGTTGTAAAATTCATCTTGAAGGAAATCGTATACTCAAATTTAGGAGAGC
ACGAGATGGAAGAAAATATATACGCGAAATCTAATKATTTAATAGTGAGGAGAAACGATATAAAGAAACTCAGGCCTAAA
GGAGATTCGAGCTTTTAAGCAACCCTGAGGGATATGAATTTACAGCACTCTTGGTAAAATATAAGGTACACATTCCAACC
ATTTGGTCCGAAATTCGATACGGATCAAGCGCGCTAGTAATTTTTTTTCAAACTTCAAGATTTCTTTACCATGAATTTGG
CAGATAGTTCGAAAGATCAGTGGGTTCATTGCATAGTTACCTTTACTGCGGCAAACGCTCCGCTGATCTGGGGAACTTTC
ACTCCCAGTATATGCTTCAATTGGCTGTTGACAACGAGCACCGCTGACCCTGTGGTGAAACCACTGATCAATGGTTCTGA
CAAGAACGTCGCCAGAAATCCCATCTTCACTGCTCCCATGATAATCTATCGTGAAAAACATTCTTTTGTTAACTACTATT
CATGTTTTGAGGCGAGGCGCAGTGGCCTCATGTTTAGTGCGCTCGACTCCGGATCACGTGGTCCTGGATGGAGTTCCGGC
CGCGGCCGGGGACATTGTGTTGTGTTCTTGGACAAGACACTTTACTCTCACACTGCCTCTCTTCATCCAATGGGGGTGTG
GGGGAGGGAGAGTAGAAATACTCCGAGTCGCTTCATCCTATGGAAACCGGAGTTAAGCACTRGCCTGATKGGCCACTTGG
CTCGTAAACAAACTTTCATTYACATTTGATAGCAAGAAAATGGTTTATCATTTTACCTGGATGATACCAACGAGTAAGGA
GAGCGAAAGGGCGACTTCCATTTTGGCCGACATTTCCGGTGTCCATAACGCGGTGCCTTCTGTCGGGGAAGGACTGGACA
TCGAGTCATTGCCCGGTGTAGGAGTKGGCGCTAATGGCATGGAACCCACTTCACGCTCAACAACTTGTGCCACCATCAAA
CATATGACTGCGAAACTACCTGTCACGTGAAAACAACAACCATTCAGTAAACTGATGACTTGTCTCACCGTTGGTGAGAC
AAGTCATCAGACAAGTCATTAGATACCGTTGGTCGATCATTTAATGCATGAAATAAGCATTGAATGATAACATCATTCCT
TCAAGCTACAGGAATATAAAAGAAAAAAAATAAATCAAAAGAAAAAAAAAGGAATGAAAAGAAAAAATATTTTCATCGTA
CCTACAGACAAGTGCCGCGATGTGCCCATGATCATATAAACCAATACTGGGATAATGGCGGTGTAGAGTCCCGTGACCGG
CGGAAGGGAAGCCAGCATGGCGAATGCGAGTCCTAACAGAGAAAATATGTCATTAGAAAAAGACATGTGACAGAAAAAAT
TTGCGTTACTGGCCCCCCAGTGATCCACATCACTCGTATTCCTCTTGAAGCGCACGAACCTCTTCCGGCCGGTAATTCGC
GACAAAGTTCTTCCGTTGATGRAACGCTCTAGGTCCGCTCTTTCCTGCAGGTGCTGGTCCCCGTAAATAAAAGACGGAAA
ATAATACAACTGAATACAAATGCTTTCAAATCTTAATAACTTCTAAACAATAGGCACCGACTCCAGCAGTGAGACTCAAA
ACCTCACAGAGGTTTCATCGAGTGTTATTGTTATTTTAACGGTTACGTAATTTACCGCCGTAACAATCCTACACCCCATA
CCTCTAAAAAAAAAAAAAACAGTTAAGGAATCGTATGCGATGATGGTCAAATATTCTTACGAGCCTACCCAAATGAACAA
CTAAAGCCAAGTTATTCAGCTTAGCGCAGTTATGTGTGTGTTAGCCGGTAATTCCTCGCTACTGCTGGGTTCAAACCAGT
TTTGTCAGTGTTTGGCCCCAGAACGCATCACGTAGAAATCATTTACAGCTGTCCTTAAACTATATATTAGAACGCGTTGG
CTCATCGGTCGGACGATGCAAAGACAAGGAGTCAGACAATCCGTATAAAATATTCCCATACCTTGAGGGACATGCATAAC
TGCTACTGTAACTCCTCCATTTAAATCAGGAAACAAATCTTCTTTCACGTTGTATTTCGGTAACCAGCGAATAATCGGCA
TGAGCCCAGCTAGAAAAGAGATCAAACCCTTCCACGAACACGAACAGGGAAAGAGCTTTGGATGAACAACGCGCCAGTAA
TAGTTCTTCTTTGCTGAGTCGTCCTTGGTACTCAATGGAAAGCGATCGCGTTTTGTTGCAGTGAAAAAGTTTTCATCGTA
AACATTTCTGGCAATATCTATTTCAGGGCTTTGGCGGTGAATTGACCGCTCTCCGGKTGAAGATTCCATTTTATTCAAAT
CTTCAGAGGAGAGTTTTACCRTCCAATTCTTATTCAAC
>scaffold_34 279234 286052

TCATGTGAAAACGCCGTGTTCAAAGTTGTATCGCTTCTTTAAATCAACACATTTGCTACACCTTTCWGTTTCCATCACAT
TGACTTTTTTTTATGGATTGACGTTATAATTCAAATAGCAGACCTTAAAAAGTRTACTGACCAATCTCAGAAAAGGCGTT
CGGGTCGAGAAAAAAGTTCGAATTTTGGAGAAAYGCGGTTAGTTACACATTCCTGTTACGTAACTTTTCGCTCTTTTAAG
ACACGGAAGACAGTAACAAACAAAGAAGCATTATTTAGAATAAGTTAACGAATTAATTAACTGATTTAAAAAGGGGGTCA
CATCGAGAAGACAGTGGTATGATCCAATTACGTAAGGGAGAGGCACGCGCCAAAAAGAGAAATGTAGTTTGATGGGGAAA
AAAAAGATCAGCTGATTTCCGCCTGTCTTCACGTGAAAAAGGATGWTTTTCAAACTGCCTTTATTATAGTGGACTCTTAT
CRGTGATTACTGTAAGCAACAATTTCAGTCTGTAACGTTCGTCAGCTTTGGTGCGGTGAAATGGCGTCGGAAGAAACTAG
CAAAAAGTTGCAGACTTTCTCGGTGAGTACTAAGGATAAAAACGCCCACGATCTATTCTGAGGGTGACTTCACCTCATCG
TAGAATTTAAAAGAATTTAGTAGAATGAAAGTTTTCTTTTCAGGGAGRAAAAGCGTTCTCGTTAAATGCTCGTGTAGAAT
TATGCGATGCCGCTTTAAATTGATGATGGAAGACGAATATTATCGATTTCCACTCGTTTTTTTCCTTTGCTCGTTGAATT
CTGAACAAATTGAACTCATCTCCTATCTAACTCTTGGATTTTAATGTATGCAAACATTGTCTCGCTGATTGTTTAGTTAC
CGCAGGCAACCATGTGAATACATGAATAGCTCAATTGGCCATGATTGATTGGTGGTTTTTTGAAATGACCTGCTTGCCGT
ACGCTAAAACAAAATGCAGAAATCAAAACTTATCGAACCTCAGGCGATTGCCGCGTCATTTCACTGTAACCAGTTCAGAA
TTCTGCCGATGCCGTTCAGCATAACCTCGGTTCGAACGCCCGTCAACGTGTCATTTTAACATGAGTTGCTCTGCTTTACA
GGGGAAATRCAGGGAATTCTGTTCTCCGGGACCGGTGAAGGTGTTTGTGAAGAAGCGCTTTCCAATCGCGTCTTGGCTGC
CAGAATATAATCTCAGGAAGCTACAGTGCGACATGATTGCCGGCTTAACTGTCGGTCTTATGGTTGTTCCTCAAGGTCTA
GCCTACGCTCAACTGGCCGGACTGCCACAACAATACGGACTATATTCGGCGTTCATGGGTTGTTTCCTCTACTGTATTCT
GGGTACGAGTAAAGATATTACTCTAGGTCCCACTGCGATTATGTCTTTAGTGGTATCTGCGTACGGAAACCCGAAATAC
CTCATTATGTTGTCGCATTAACTTTATATACTGGAATAATCTTACTCGCTATGGGCTTTCTTCGGTTGGGATTCGTTGTA
AATTTTATTTCTATTCCAATCGTGAGCGGGTTTACCTCCGCAGCGACAATAATCATCGCCTTTAGTCAGCTGAAAGATTT
GTTTGGATTACAAAAAATTCCTCGTAAATTCGCCCAGAACGTGTACTTCACCTTCAAGAATATCGGTCAGACAAACAAAT
GGGACCTAACATTGGGGTTAWTATGTATAATAATTCTAATAACCCTGAGGAAGGTTGGACGACTCGAATGGGTGAAGCGC
AAGGACTCGAGCGACTCCAGATGGTTGAAGGCAGCAAAGAAGACTGTTTGGCTCATTTCAATCAGTAGAAATGCTCTCAT
AATTTTGATCGCTGCAGTGGTTTCTTCGTCTCTTTATCAGCATGGCCATAAAGATATTTTTACTTTGCCATCGCGTATTA
AACCGGGTCTTCCACTTATGCAGGTAATTAAATGTTTACTTGATATTTTTCATGATCGTTTGAAGCGACACCTCATCAWT
GTAATTGCTCTACCGGCCAAGCACATAATTCGCTTCAGAAAAAAACGTTACAATTTGTGGTTTTCGCAGGGAAAAAGCGAA
TTTCATTTAACTCTCTATGGATGACTAAGAATCCTCAAATTAGAACGTCATTTCGAATTGTAAGTTTTCCCGTGACTGAT
TTTTTGAGGGGTTGAAGCACTTACGCCATCGGTCAAATTTTTAAATAGAACGTAAGCAAAGCCTTTTTCGCGGCCTCTGA
TTGGGCAAGAGCTCAGGAATTCTTTCAAGCGCGCGAAAAATCCCTTGGAAACGTTAGCGATCAACGTGCTTGAGCGTATT
TTTTCGCTCAGAAAATGATCATTTCACATGATCTTGATTTGTTTCGGCAAACTAWACATTTAAGCTACTTGTAAAATAGA
CTTGAAAATATGTTATTTTGGTCCCTAAAGGGTACTTCAAAATTATTTCCCCTAAACTTTGAGTTTGCCTGGCAACTCAT
TCTAATCTTTGGCACAATTACTTTTCTCAATTAAAAACATAGTGAAGTGCAAGCAAATACCCTTAAAGTAGTTATGTAAA
GTAATGAGTTATGCTAATTTTTTTTTTAAACAATTTATTGTTTTCCACCATCAACCACAGATTCTAAAGATTTGAAAATT
TTGACCAGGCAAACATCTGTCAATGAATGTTTTGTTTTATTAAAAAGTTACTTATTTTCATGGATTTTGCATTTAGAATA
ACAGAATACAATAACTGATGGAATAACCTCTTAGAAGTATTTATTCAAAAAGTATTTGAAAACAATTTTTATTTTAAAGA
AATCTCTGGCACATAGCCTTTGATTTTACATCTTGAAATTTGWGGATTATGAAGCTTAGTGCATGAAGTTTAGTGTGTAT
GTGTGCATGTGTTTTAGGTGTTTATTTTTATAGTTTTTGTTTTCTAATATTGCAACACATATGTACCAAATTGTACTTAT
TGTTAGTCAAAAGCAATGTAACTCTACATCCCCCATGGTTAGATTGTTCTCAGGGGCGACTTGTTCAAAACTTGGTTAAG
TTAACCAAGGGTTGGTGCAAAATCTGATTTCAGATGAGAAAGGTTTAAAAGAAAATTCCATTTAACTCTTTTTGTCCATA
ATTTGATAATTGCACACTCAGAAAACACTCAGCTACAACAAGTTAAAAAAGGATGGAGATAAAAGATCCTTCAGTTGGCA
ATGTGTAAAGGGCTAACTAACAGCTCATGCAAAAGTGAAAACAGCATTTTACTAATTTTTTTCCACCAGAGTGGGTTAGA
GGGTGTGAATGTGGGAGAATCCATAGGGAGGACGGGGGTATAGAAGGGGAAAGGGTCAGGGGGTGTGAATGAGAGGGTGC
AAGGGGGTGAGTGTGTTAGTGTTGGAAAATTGATGGGAATGGAAGGAGTATGGGAGAGGAAAAGGTAAAAGTAAAGTGTT
TTTACAAGCCTAGTGGCCCATTAGGCTGGTGCTTAACTCCGGTTCCTGTAGCATGAAGCAACTAAGAGTATTTGAGGGTT
ACCCCCAGCATTAAATTGCAAGTACCCATTTAAACACCTGGGTGAAGAGAGGCACTGTGAGAGTAAAGTGTCTTGCCTAA
GAACACAACACAATGTCCCGACTGGGGCTCGAATCCAGACCACTCGATCCGAAGTCGAGCGCACCAACCATGAGGCCACC
GCACCTCCAATGGGAGAGGCATAAAAAATGAGACAATTGAGTTAAAGGTGCGAGGGGAATAGAACAGGATTTGGGAACTA
TCCAAATAATTAAATAGGAGARGGGGTGGCCAGATGGATGGCAAACAGAATTCAATGGGGCTGTGTTGAGCCCTGGCCAT
TGCCACTGAACTCTMTTCTTGGGCAAGAAACTTTCTGCCTGCAGTGCTGTACCCTGGACTTTTCTTTCTTTACACTYTAC
AGGTGCCTGCTTTGAGCTTTCAAGTTGGCAATGCTACAAGAAGCACCTTGGAGGTGTTTAAGGATCTTGGACCTGGTCTG
GCTGTGGTTCCCCTTATTGGCTTTCTCGAGAGCATTGCAATTGCCAAGGCCTTTGCACGTAAAAATCGCTACACTGTTGA
TGCCAGCCAGGAATTGATTGCCCTTGGTGTTGCCAATTGTCTCAGCTCATTTGTATCCTCCTATCCAGTGACTGGAAGCT
TCTCTAGGACTGCTGTGAATGCACAAAGTGGAGTGGCAACACCTGCAGGAGGTTAGTTTTCCAATGGAACACTTGTACCC
ATTTTTCTGTGTTTTGTTTTTTTTTGTTTTTTTTTTTGTTAGAGTAAGCTGCAGGTTTCAGTTGGTCCCGCCCCTGCAGT
AAATCATTATCCATCTCTTTCATTAAGACTGTAACTTTCAATCAAAGACTTGGCCCAAACAATCAATCAACTTGGCATTA
CTTYTGGTTCAGTATTTCCAGGACAATCCTGTGCACATTTGTTAAGGATTTTCTTGATATCTTTTCATTTTTAAATGGCA
TTCCACAGGATTTGGGAACTATCCAAATAATTAAGTAGGAGAAGGGGTGGCCAGGTGGATGGCAAACAGAATTCAATGGG
GCTGTGTTAAGCCCTGGCCATTGCCACTGAACTCTATTCTTGGGCAAGAAACTTTCTGCCTGCAGTGCTCTACCCTGGAG
TTTAAATAAAAAAAAAAAAAAAATGAAAGTCTACACTTCCCCCCCTTCCCCCATCTGGATGGAGTACCCATCCACTGCGGG
TTACCCCAACTGCATTTTGTCTTGTTTCCTTTAGTTTGTCAGAACCCACAAATACTCTCAGGTGGTGGAGAGACGCAGTG

CCTGGCCAAAGAACACAGGGCAATGACCTAGCCAGTTCTCCAACTCTGATCTCTCACTTCTAGTTCCAGCATGCTAACTG
TTAGGCCAAGGGGGAACAAGGTGAAATGCTTAGTGCCCTATTCACCAAGAGTACCAATATTCCCAGTATACTTGTGCCTM
AAAAACTGGGATATGCCCTGGCAGCTATATTCTAGTTAGTCTTGTATACAGGCTGAAATTGATGTCTTTAATTTTATACA
TTTGTTTTTTTTTTTTTTTTAGGAATTTTCACAGGTGCTATTGTTCTCTTAGCCCTTGGACTCCTGACAGAYTCCTTTAA
GTATATTCCAAAAGCATCTCTTGCAGCACTGATCATGTCCTCRGTGATAACCATGATTGAATATCACATTGTGCCCAACA
TCTGGAAAGTGCGGCGCATAGACTTGGTGCCTTTGGCTATCACCTTCTTTGGCTGCTTCTATGACATTGAAGTTGGAATT
TTGGCAGGTATTGCAGTTGCCTTRTGCATTCTCCTGTATAATGTGGTGTGGCCCCCCATTACAAGAATAGCTAGAGGAGA
CTATGTGCTTCTAAAAATCAAYGGAAATCTCAACTATCCTGGCRTTGAACATCTGACCAATGAGATTCAAGAAGTCRCAG
CKATGGAACCTTCCCCTCCTGGTATTGTAATCGATTTTTCTCTGGTCACAAGCATTGACTTCACAGTCACTCAAGCACTT
CTGACRATCCTGGAAGATATGGAAAACAAGAGAATTCCTATATTCTTCTCTGGTGTTCAGGACARTGTGCGAAATATGAT
GATGAACTCTGGGATTGATTCAGGCATTATTAATCAAGGCACACAAGCTGTCATAGACTCAATTAATTCTTTGGAAATAG
TKGAACAAAATTGAATCAATATTAAAGCATCAACAGTGAATAGGGTAGTAAAGTTTCAGGAAACTATTTTGGTTACAGAT
TAGGCAAATGAAACAGTAAACTTTGATGATGACAATGACAATGAGAACATTTTATAGGGAAAGCTGTTAGGATTATCCAT
CCACAAAAAATACCACTAAAATTACCAATACATTTACTGGGTGAAATTTTTCTGTGCTACACACAATTTGAAAGTTTGCA
GCATTGTAAGGAGATATTTTCAAACTATAGCATACTTTCTTTTTCTGTTGTTGKACCTCAAATTYTTCCYACTGCAGGC
AAATTACTCAAAYGTTAATTCATGTATTAGTCCTTTTTTTTTTTGCCTTTCCAAGAGAATCCAACTACAAGTCTTGTATA
GAGCAAACTCCATTGTGTATGTAGGTYACATTGTGTCTGAAAGGACTGCAAGAGTWTARGGGATACTGTAGACCTTCACA
GGTGTCCTTTKCCCAAACATCTTTCAATGCACTATTTGAAGGATAACCACCCTTACCTACATATCAATGGAAAGGTTATG
AAAWGCACTATTTGGCAAAAATTCTTTTGTGCAACCAAAATCAGGAGATGTTTAGGTAATTGAAATTCAAGGACCAGGAT
ATTTTGCCACAAAAAACTCTTGGGGAGTCATTTATATTGACATGTTATAGTTGGCTATTCACACCTTGGTGGGCGAAAGA
ATTAATTTGGAAAATATTGTGTCTGTTTATAACTTGTGTGTGTTTCAATATTGTACCAAGTTTTTCTTTGAAACTATAAT
TAAAATAATAAGGCTTAGTTTGACATGAAAAAAAATTGAAAAAATTGAAAAAACTTGGCCTCCTTAGGTCTACTGTATCC
TTAAAAAGTTCAATATTTGTATTCATTTTAGAAAAGTAAAGGTTCCATGATCATAGTTAACCATTATGTAGGTCAATCAG
AGTGATATACTAAGATTAATGCTAAATTTCATTAGATTAACACAGAAAATCATTCTATATTATTTAAAGACAATTTACAT
TGTGATTTTGCTATTAATTCCATGCATTAGTTATTTATTCATTGTGCTTAGATTCAGAATTTCTCTTCAAGTGTAAGTTA
AAACAAGCACCCCAGACA

Table S5 : qPCR primer list

Target	Forward primer	Reverse primer
36B4	ATGCAGCAAATCCGTCAGTCTCT	TGGCCTTTCTTATCATGGTGTTCT
SLC4α	AACGGGAAAATTTTGTGGTGGACT	TAGGAGCGAGGCAAGCGAAATACA
SLC4β	CCAATGTTGCGTTCGGTAGTCTTT	GCCCTGTAGCGCCAATAATAATCA
SLC4γ	CTTGGAGGGCTGTTTCTTGAT	TATTTTCCGCTCCCTACCCTGTTC
SLC4δ	TCTTGCTGGTGGAATTGGTGGAGT	TAAACGGAATGGCATGTAGTCAAG
SLC4ε	GGCTGCCTACCCACCAAACCA	GGGGCAAATCCAAATCCACATAG
SLC26α	GCGAAATTTCCTGCCATCTA	TCGCCACGGTTATCCCTCCTGCTA
SLC26β	CATCGCGGCACTTGTCTGTAG	GTCCATAACGCGGTGCCTTCTGTC
SLC26γ	GATTGCCGCGTCATTTCACTG	ATTCCCTGCATTTCCCCTGTAA
